# Maternal undernutrition inhibits fetal rumen development: novel miRNA-736-mediated dual targeting of E2F2 and MYBL2 in sheep

**DOI:** 10.1186/s40104-025-01321-7

**Published:** 2026-01-06

**Authors:** Peng Jiao, Yun Xu, Yamei Gu, Baoyuan Li, Huizhen Lu, Caiyun Fan, Wen Zhu, Jianbo Cheng, Shengyong Mao, Mianqun Zhang, Yanfeng Xue

**Affiliations:** 1 College of Animal Science and Technology, Anhui Agricultural University, Hefei, 230036 China; 2 Biotechnology Center, Anhui Agricultural University, Hefei, 230036 China; 3 College of Plant Protection, Anhui Agricultural University, Hefei, 230036 China; 4 College of Animal Science and Technology, Nanjing Agricultural University, Nanjing, 210095 China

**Keywords:** E2F2, Fetal rumen development, Maternal undernutrition, MYBL2, Novel miRNA-736

## Abstract

**Background:**

Undernutrition disrupts pregnant ewe’s metabolic homeostasis and severely inhibits fetal growth and development. In this study, undernourished and nutrition-recovery pregnant sheep models and rumen epithelial cells were utilized to investigate the mechanisms behind undernutrition-induced disruptions in male fetal rumen metabolism and development.

**Results:**

Maternal undernutrition significantly reduced male fetal rumen weight and papilla length, width and surface area. Maternal undernutrition extremely suppressed nutrient metabolism and energy production in male fetal rumen via JAK3/STAT3 signaling to inhibit cell cycle progression and male fetal rumen development, while maternal nutritional recovery partially restored metabolic inhibition but failed to alleviate male fetal rumen development. Meanwhile, 64 differentially expressed miRNAs (DEMs) were identified in male fetal rumen between undernourished ewes and controls. Novel miR-736 was overexpressed both in male fetal rumen of undernourished and nutrition-recovery models. E2F transcription factor 2 (*E2F2*) and MYB proto-oncogene like 2 (*MYBL2*) were the intersection of male fetal rumen differentially expressed genes (DEGs) and DEMs target genes integrated analysis and were predicted as novel miR-736 target genes. Further, we confirmed that novel miR-736 targeted and downregulated *E2F2* and *MYBL2* expression levels. Silencing *E2F2* and *MYBL2* promoted apoptosis and inhibited S-phase entry in rumen epithelial cells.

**Conclusions:**

In summary, maternal undernutrition disrupted male fetal rumen metabolism and elevated novel miR-736, which targeted and downregulated *E2F2* and *MYBL2* to inhibit cell cycle progression and promote apoptosis, finally inhibited male fetal rumen development. This study provides new insights into the epigenetic mechanisms underlying maternal undernutrition-induced male fetal rumen developmental deficits.

**Graphical Abstract:**

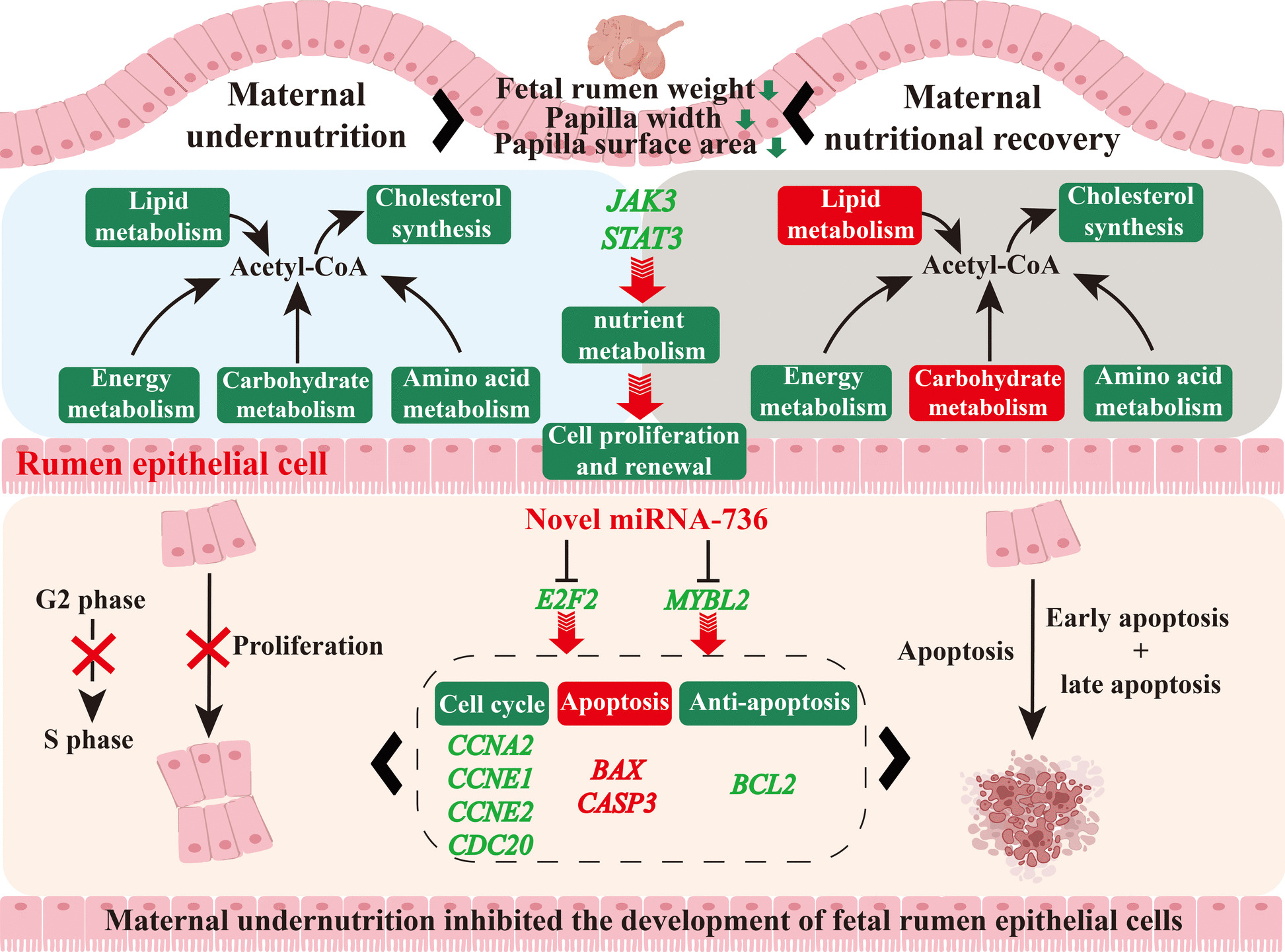

**Supplementary Information:**

The online version contains supplementary material available at 10.1186/s40104-025-01321-7.

## Introduction

In ruminant production system, many pregnant animals are subjected to undernutrition, a condition that may be exacerbated by seasonal fluctuations of feed availability or by cost control-driven insufficient feed supply or low-quality rations [1]. Due to the multi-fetus characteristic of Hu-sheep and the rapid fetal growth and development during late gestation, pregnant ewes are prone to undernutrition which ultimately inhibits fetal development [2]. Rumen, as a critical digestive organ in ruminant, requires early and optimal development to ensure both animal health and productivity [3]. The transitional period (pre-rumination) in young ruminants represents a sensitive window for influencing the development of rumen wall [4]. A large body of researches have shown that varying kinds of nutritional regulation promote rumen development of lambs [5–7]. The enhancement of rumen wall morphology and physiological function through early-stage nutritional strategies holds significant potential for supporting the lifelong health and productivity of ruminants. However, the effect of maternal nutritional status or nutritional regulation on the morphology and development of fetal rumen before fetal birth remains underexplored. The loss of physical function caused by undernutrition in ruminants may be compensated by subsequent nutritional recovery [8]. Metabolic disorders and pregnancy toxemia resulting from malnutrition during late pregnancy can be effectively ameliorated through nutritional recovery [9, 10]. However, whether the effects of maternal undernutrition on fetal rumen development can be alleviated by maternal nutritional recovery has not yet been studied.

Ruminal papilla are basic structures of ruminal epithelium and associated with great contact with chyme, enhancing the digestion and absorption of nutrients [11]. Our previous study has shown that undernutrition significantly decreases maternal rumen weight and the length, width, and surface area of rumen papilla [12]. Meanwhile, maternal undernutrition seriously affects fetal weight and fetal liver development [2]. However, the effects of maternal undernutrition on fetal rumen development and the potential regulatory mechanisms remain poorly understood. Undernutrition of pregnant ewes has been found to inhibit the metabolism of carbohydrates, amino acids, and energy in the rumen and to suppress DNA replication and cell cycle in rumen epithelium through JAK3/STAT2 signaling pathway, ultimately leading to the narrower, shorter, and small rumen papilla [12]. Therefore, it is supposed that maternal undernutrition may affect nutrient metabolism and cell cycle in fetal rumen to inhibit its development via some key signaling pathways. As known, miRNAs are key regulators of various biological processes, and fetal plasticity during pregnancy is regulated by miRNAs [13, 14]. miR-21-3p has been identified in sheep rumen where it regulates amino acid metabolism by targeting *ATP1A2* [15], while miR-148a-3p inhibits the proliferation of rumen epithelial cells by targeting *QKI5* [16]. However, whether maternal undernutrition can induce differentially expressed miRNAs (DEMs) in fetal rumen and further regulate the growth and development of rumen epithelial cells remains unclear.

This study postulated that maternal undernutrition during late gestation might inhibit male fetal rumen development through regulating miRNA expression to disrupt cell cycle and rumen nutrient metabolism, and maternal nutritional recovery after undernutrition could only partially restore the nutrient metabolism homeostasis and male fetal rumen development. Undernourished and nutrition-recovery pregnant sheep model were established using dietary nutrition control. Both in vivo animal models and in vitro sheep primary rumen epithelial cells were integrated to investigate the molecular mechanism of how maternal nutrition affects male fetal rumen development. This study would provide theoretical foundations for the development of molecular therapeutic strategies to promote male fetal rumen development.

## Methods

### Animal experimental design and sample collection

The animal experiments were approved by the Institutional Animal Care and Use Committee of Anhui Agricultural University (AHAUXMSQ2024073). The diet formula and nutrient composition for both undernourished and nutrition-recovery ewe models were detailed in Table 1. In this study, the metabolic energy and crude protein level of the total mixed ration were 11.25 MJ/kg and 12.60%, respectively. The farm was located in Hefei (31°57′20" N, 117°03′26" E, at an altitude of 49.8 m) with an annual average temperature of 16.1 °C. The ewes were housed in individual pens, fed twice daily, and had free access to water. In addition, all ewes’ body weights were measured using a calibrated electronic scale with an accuracy of 0.01 kg.

**Table 1:** Ingredients and nutrient composition of the total mixed ration, % (dry matter basis)

Item	Content
Ingredient composition	
Whole corn silage	43.04
Peanut straw	31.07
Maize	17.79
Bean pulp	4.04
Soybean meal	2.70
Premix^a^	1.36
Total	100.00
Nutrient composition	
Metabolizable energy^b^, MJ/kg	11.25
Crude protein	12.60
Ether extract	3.20
Neutral detergent fiber	40.19
Acid detergent fiber	34.20
Crude ash	5.23
Calcium	0.61
Phosphorus	0.31

^a^Provided the following per kilogram premix: vitamin A 150,000 IU, vitamin D_3_ 25,000 IU, vitamin E 1,000 mg, Zn 2,548 mg, Cu 370 mg, Fe 1,500 mg, Mn 637 mg, I 35 mg, Se 11 mg, lysine 1.5%

^b^The metabolizable energy was estimated

Undernourished ewe model: Twenty ewes (body weight 54.41 ± 2.48 kg, 2–3 fetuses, pregnancy for 108 d) were housed in individual pens and ad libitum fed for a 7-d adaptation period. This period served to determine their voluntary feed intake and to ensure that the dietary nutritional level met the requirements of pregnant ewes carrying multiple fetuses. Subsequently, these ewes were randomly assigned to the CON group (*n* = 10, fed the baseline feed intake) or the UN group (*n* = 10, restricted to 30% of the baseline feed intake) on d 115 of gestation for 15 d treatment. According to the nutrient requirements of meat-type sheep and goat (NY/T 816–2021) [17], the metabolic energy and crude protein requirements of ewes carrying multiple fetuses during late gestation were approximately 19.8 MJ/d and 252.5 g/d, respectively. Considering the feed intake was 2.06 kg/d (dry matter) for ewes in the CON group, the metabolic energy and crude protein intakes were 23.2 MJ/d and 259.6 g/d, respectively, which could meet the nutritional requirements. Blood samples (10 mL/ewe) were collected from the jugular vein using 18G needles and vacutainers before morning feeding on d 0 and d 15 of the formal experiment period [10, 18]. On d 130 of gestation, all ewes were humanely slaughtered using electrical stunning followed by exsanguination at 4 h after morning feeding [19]. Male and female fetuses were carefully dissected out and their rumen tissues (CON: *n* = 10, UN: *n* = 10) were weighted and then were rapidly collected from each fetus, snap-frozen in liquid nitrogen, and stored at −80 °C.

Nutrition-recovery ewe model: Twenty four ewes (body weight 54.64 ± 1.69 kg, 2–3 fetuses, pregnancy for 108 d) were similarly housed in individual pens and ad libitum fed for a 7-d adaptation period. Ewes were then randomly assigned to the CON group (*n* = 12, fed the baseline feed intake for 15 d followed by ad libitum feeding for another 15 d) or the REC group (*n* = 12, feed intake restricted to 30% of baseline for 15 d followed by ad libitum feeding for another 15 d) on d 115 of gestation for 30 d treatment. Due the predelivery of 3 pregnant ewes in the nutritional recovery period, they were removed from the research subjects. Blood samples (10 mL/ewe) were collected from the jugular vein using 18G needles and vacutainers before morning feeding on d 0, d 15, and d 30 of the formal experiment period in nutritional recovery ewe models [10, 18]. On d 145 of gestation, all ewes were humanely slaughtered using electrical stunning followed by exsanguination at 4 h after morning feeding. Male and female fetuses were carefully dissected out and their rumen tissues (CON: *n* = 9, REC: *n* = 9) were weighted and then were rapidly collected from each fetus, snap-frozen in liquid nitrogen, and stored at −80 °C.

### Measurement of ewes’ blood biochemical indicators

Levels of *β*-hydroxybutyrate (BHBA) in maternal serum were measured using the Free Style Optium Blood Glucose and Ketone Monitoring System (Abbott Diabetes Care Ltd, Oxon, UK). Then, glucose (Cat. F006-1-1), non-esterified fatty acids (NEFAs, Cat. A042-2-1), triglyceride (TG, Cat. A110-1-1), and total cholesterol (TC, Cat. A111-1-1) levels in maternal serum were determined using commercially available kits according to the manufacturer's instructions (Nanjing Jiancheng Bioengineering Institute, Nanjing, China).

### Fetal rumen weight and morphological analysis

Chyme was immediately removed from male and female fetal rumen after slaughter, and the weight of the empty male and female fetal rumen was measured. Male fetal rumen specimens were collected, fixed in 4% paraformaldehyde for 24 h, embedded in paraffin, and sectioned for hematoxylin and eosin staining for morphological observation [12].

### Transcriptome assay of rumen epithelium

Total RNA was extracted from cryopreserved male fetal rumen tissues using TRIzol reagent (Takara Bio, Otsu, Japan). RNA quality was verified by spectrophotometric analysis (NanoDrop ND-1000) with A_260_/A_230_ and A_260_/A_280_ ratios between 1.80–2.10, and RNA integrity was confirmed using an Agilent 2100 Bioanalyzer. A total of 20 RNA samples were randomly selected for transcriptome analysis, with 10 samples from undernourished model (five CON and five UN) and another 10 samples from nutritional recover model (five CON and five REC). The mRNA samples were purified using the magnetic bead method, fragmented, and reversely transcribed into cDNA, which were then ligated to sequencing adapters and amplified. Fragments with appropriate length were selected to construct cDNA library using NEBNext^®^ Ultra™ RNA Library Preparation Kit and sequenced on an Illumina NovaSeq 6000 (Biomarker, Beijing, China). Clean reads were aligned to the Sheep Reference Genome 3.0 using HiSAT2, and gene expression was quantified using FPKM method. DEGs were identified with DESeq2 (v.1.6.3) based on *P* < 0.01 and fold change (FC) > 1.5 or < 0.67. Principal component analysis (PCA) and partial least squares discriminant analysis (PLS-DA) were performed using SIMCA v.14.1 (Umetrics, Umea, Sweden). Data analyses including clustering heatmaps, KOG functional classification, and GSEA analysis were conducted using the Biomarker platform (www.biocloud.net).

### miRNA-Seq library construction, sequencing, and data analysis

Small RNA library was constructed using 1.5 µg of total RNA with a NextFlex Small RNA Sequencing Kit (Illumina, San Diego, CA, USA). Briefly, small RNAs (18–30 nucleotides) were size-selected via gel electrophoresis, followed by sequential ligation of 3' and 5' adapters. After adapter ligation, reverse transcription and PCR amplification were performed with 12 cycles to enrich the library. Library quality was assessed using an Agilent Bioanalyzer 2100, and the quantification was performed by Qubit 3.0 Fluorometer (Thermo Fisher Scientific). The library was subsequently sequenced on the Illumina HiSeq 6000 platform using single-end sequencing. Raw sequencing reads underwent quality control using FastQC to remove low-quality reads, adapter sequences, and potential contaminants. Clean reads were aligned to the Sheep Reference Genome 3.0 using Bowtie2, allowing for the identification of known miRNAs and the discovery of novel miRNAs. The analysis of miRNA expression levels and the identification of DEMs were conducted using DESeq2 (v.1.6.3) with the thresholds of *P* < 0.05 and FC > 1.5 or < 0.67. Additionally, potential target genes of DEMs were predicted using miRanda software. KEGG pathway enrichment analysis, network map of miRNA target genes, and Venn diagram analysis were conducted to explore the biological functions and regulatory pathways of these miRNAs in the male fetal rumen development. Subsequently, STRING software was used to analyze the protein–protein interactions (PPIs) of the target genes (*E2F2* and *MYBL2*) and to perform Gene Ontology (GO) pathway enrichment analysis.

### Cell culture assay

Primary rumen epithelial cells were cultured in DMEM/F12 medium (GE Healthcare Life Sciences, Hyclone Laboratories, South Logan, Utah) supplemented with 10% fetal bovine serum (FBS; BI, Israel Beit Haemek Ltd.). Human embryonic kidney (HEK) 293 T cells were cultured in high-glucose DMEM (GE Healthcare Life Sciences, Hyclone Laboratories, South Logan, Utah) containing 5% FBS (BI, Israel Beit Haemek Ltd.). All cell cultures were maintained under standard incubation conditions of 37 °C and 5% CO_2_.

### miRNA mimic, inhibitors, and siRNA

The mimic of miR-736 (double-strand oligonucleotides), mismatched negative control mimic (mimic NC), miR-736 inhibitor (single-strand oligonucleotides), and mismatched negative control inhibitor (inhibitor NC) were synthesized by RIBOBIO Co., Ltd. (Guangzhou, China). These mimics and inhibitors were used to assess the effects of miR-736 overexpression and inhibition on its activity in HEK-293 cells and rumen epithelial cells. The small interference RNA (siRNA) for *E2F2* and *MYBL2* were synthesized by Sangon Biotech (Shanghai, China). The sequences for the si-NC and siRNA primers were provided in Table S1.

### 3’ UTR luciferase reporter assays

Primers targeting the 3' UTR regions of *E2F2* and *MYBL2* were designed using Primer Premier 5.0 (Premier Biosoft, Palo Alto, CA, USA). Detailed primer sequences are provided in Table S2. Then, RNA from rumen epithelial cells were extracted and reversely transcribed into cDNA using Primescript™ RT reagent Kit (TaKaRa, Dalian, China). The 3’ UTR fragments of *E2F2* and *MYBL2* were amplified using the following 20 μL reaction system: 0.8 μL of forward and reverse primers, 1 μL cDNA, 10 μL Taq PCR Master Mix, and 7.4 μL ddH_2_O. The resulting 3’ UTR double-stranded cDNA fragments of *E2F2* and *MYBL2* were enzymatically cleaved and cloned into the *XhoI*-*NotI* sites of the psiCHECK™-2 vector (Promega Corp.), which contained both Renilla and Firefly luciferase reporter genes, forming the wild-type dual luciferase reporter gene recombinant vector. The psiCHECK2-E2F2 and psiCHECK2-MYBL2 recombinant plasmids were extracted using the EndoFree Plasmid Mini Kit (Omega Bio-Tek, Norcross, GA, USA), and the reliability of these recombinant vectors was verified by DNA sequencing.

HEK-293 T cells were seeded into 24-well plates at a density of 1.0 × 10^5^ cells/well. After 24 h, cells were co-transfected with 0.5 μg of either psiCHECK2-E2F2 or psiCHECK2-MYBL2 recombinant plasmid and 0.5 μg of miR-736 mimic (or control NC_mimic) using 2 μL of X-tremeGENE HP DNA transfection reagent (Roche, Penzberg, Germany) following the manufacturer’s protocol (06365752001a.fm). After 48 h, Renilla luciferase activity, normalized to Firefly luciferase activity, was measured using the Dual-Luciferase^®^ Reporter Assay System (Promega, Madison, WI, USA) according to the manufacturer’s instructions. A decrease of more than 30% in the Renilla to Firefly luciferase ratio in the psiCHECK2 3’ UTR + mimic-transfected group compared to the control group indicated that miRNA interacted with the target gene 3’ UTR.

### Rumen epithelial cell transfection assays

Rumen epithelial cells were plated into 6-well plates and allowed to reach 60%–70% confluence. Transfections were then performed following the previously described protocol [20]. Briefly, rumen epithelial cells were transfected with 5 μL of miR-736 mimic or mimic NC, 10 μL of miR-736 inhibitor or inhibitor NC, and 5 μL of siRNA or si-NC, respectively, when the cells reached 60%–70% confluence. There were three replicates for each group. After 48 h, cells were harvested for RT-qPCR and flow cytometry analysis.

### Real-time quantitative polymerase chain reaction

The mRNA expression level of key genes involved in nutrient metabolism, cell cycle, and apoptosis in both rumen samples and cultured cells were quantitatively measured by RT-qPCR. The primer sequences for the target genes, which were designed using Primer3 software and synthesized by General Biology Co., Ltd. (Chuzhou, China), were provided in Table S3. RT-qPCR was performed in a 20 μL reaction mixture, consisting of 10 μL SYBR Green I Master Mix, 0.8 μL of each primer, 0.4 μL of ROX Reference Dye II (50×), 2 μL of cDNA template, and 6 μL of enzyme-free deionized water. Fluorescence detection was carried out using the ABI 7500 real-time PCR system (Thermo Fisher Scientific), and gene expression was normalized to β-actin using the 2^−ΔΔCt^ method.

### Apoptosis and cell cycle detected by flow cytometry

The Annexin V-FITC/PI Apoptosis and Cell Cycle Detection Kit (No. C1052) from Beyotime Biotechnology (Shanghai, China) was used to assess cell apoptosis and cell cycle progression. This kit utilizes Annexin V-FITC in combination with propidium iodide (PI) staining to distinguish viable cells, early apoptotic cells, and late apoptotic or necrotic cells. In the experiment, cells were first washed with pre-cooled PBS and digested with EDTA-free trypsin to prevent interference with Annexin V binding to phosphatidylserine (PS). The cells were then resuspended in 1 × Binding Buffer, stained with Annexin V-FITC and PI, and incubated at room temperature for 15–20 min in the dark. Apoptotic cells were analyzed by flow cytometry (Beckman Coulter, USA), and data were processed using FlowJo software. For cell cycle analysis, rumen epithelial cells were fixed with 70% ethanol, washed with PBS, and stained with RNase A and PI solution. DNA content was analyzed by flow cytometry (Beckman Coulter, USA), and the cell cycle phases were determined using ModFit software.

### Statistical analysis

In the period of animal experiment design, power calculation showed that a required sample size of 10 ewes in each group could enable the detection of an effect size of 1.7 SD for fetal rumen indicators with 95% power and a type I error of 5% based on the independent sample *t*-test. The RT-qPCR data were analyzed using a two-tailed *t*-test for comparisons between two groups or one-way ANOVA for comparisons among three groups. Pearson’s correlation test was applied to analyze the relationships among rumen weight, length, width, surface area, nutrient metabolism, energy production, and cell cycle related gene expression in male fetal rumen. GraphPad Prism v.9.0 software was used for graphical representations, and all data are presented as the mean ± SEM. Statistical significance was set at *P* < 0.05.

## Results

### Maternal undernutrition induced ewe’s metabolic disorders and male fetus’ maldevelopment

**Fig. 1:**
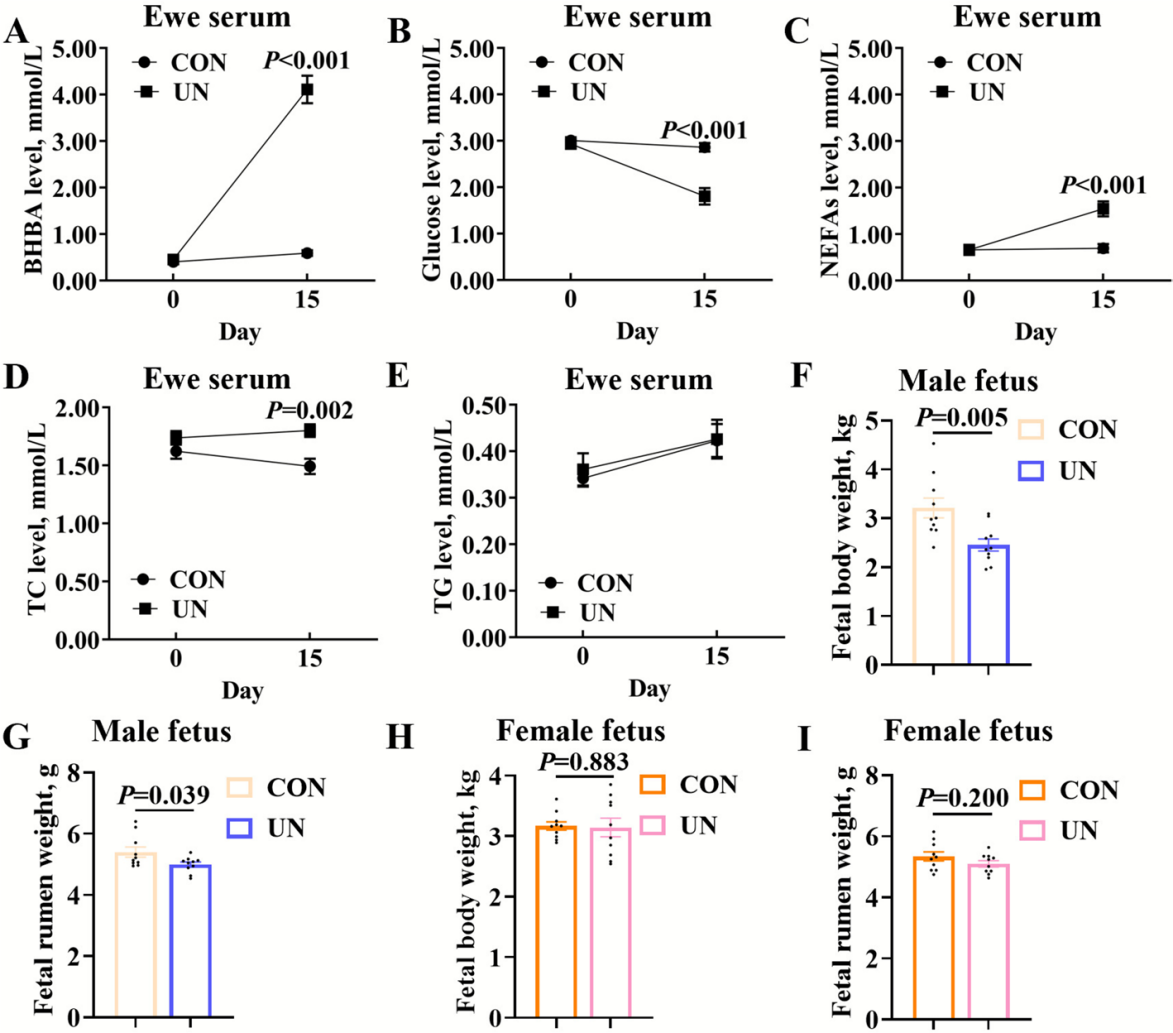
Maternal undernutrition induced ewe’s metabolic disorder and fetus’ maldevelopment. **A** BHBA, **B** glucose, **C** NEFAs, **D** TC, and **E** TG levels in undernourished ewe serum. **F** Male fetal body weight and **G** Male fetal rumen empty weight in undernourished models. **H** Female fetal body weight and **I** Female fetal rumen empty weight in undernourished models. Data were presented as mean ± SEM. Significance was assessed by two-tailed unpaired Student's *t*-test and repeated measures ANOVA

To investigate the effect of maternal undernutrition on fetal rumen development, an undernourished pregnant sheep model was established using 15-d feed restriction. At the experimental outset, no significant difference in initial body weight was observed between the CON and UN groups (*P* = 0.259) (Table 2). After 15 days of treatment, the body weight of ewes in the UN group was significantly lower than that in the CON group (*P* < 0.001), and the average daily gain was also significantly lower than that in the CON group (*P* < 0.001) (Table 2). Then, maternal undernutrition increased BHBA levels (*P* < 0.001) (Fig. 1A) and reduced glucose levels (*P* < 0.001) in ewe’s serum (Fig. 1B). Compared to the control group, maternal blood glucose level decreased significantly and was lower than the normal level (3 mmol/L) while maternal blood BHBA level elevated significantly and was higher than the normal level (0.8 mmol/L) in the feed restriction group [21, 22], which confirmed the successful establishment of undernutrition sheep model. After 15 days of treatment, compared to the CON group, ewe’s serum NEFAs and TC levels were significantly increased in the UN group (Fig. 1C–D) while ewe’s serum TG levels didn’t alter (Fig. 1E). Interestingly, compared to the CON group, male fetal body weight (*P* = 0.005) and rumen tissue weight (*P* = 0.039) were significantly reduced in the UN group (Fig. 1F–G), while female fetal body weight (*P* = 0.883) and rumen tissue weight (*P* = 0.200) were not significantly affected (Fig. 1H–I). Therefore, maternal undernutrition impaired rumen development in male fetuses but not in female.

**Table 2:** Growth performance and daily feed intake of ewes in the CON and UN groups (*n* = 10)^1^

Item^3^	Groups^2^		*P*-value
	CON	UN	
Initial body weight, kg	55.07 ± 0.84	53.74 ± 0.69	0.240
Final body weight, kg	56.83 ± 0.68^a^	44.65 ± 0.80^b^	< 0.001
Daily weight gain, g/d	117.33 ± 28.03^a^	−606.00 ± 50.78^b^	< 0.001
Daily feed intake, kg/d	1.64 ± 0.01^a^	0.52 ± 0.01^b^	< 0.001

^1^The data were expressed as the mean ± SEM

^2^CON, control group; UN, undernutrition group

^3^Initial body weight, body weight measured on d 0; Final body weight, body weight measured on d 15

^a,b^ Means with different letters are significantly different (*P* < 0.05), whereas those sharing a common letter or with no letter do not differ significantly

### Maternal nutrition recovery restored ewe’s metabolic disorders but failed to recover male fetus’ maldevelopment

**Fig. 2:**
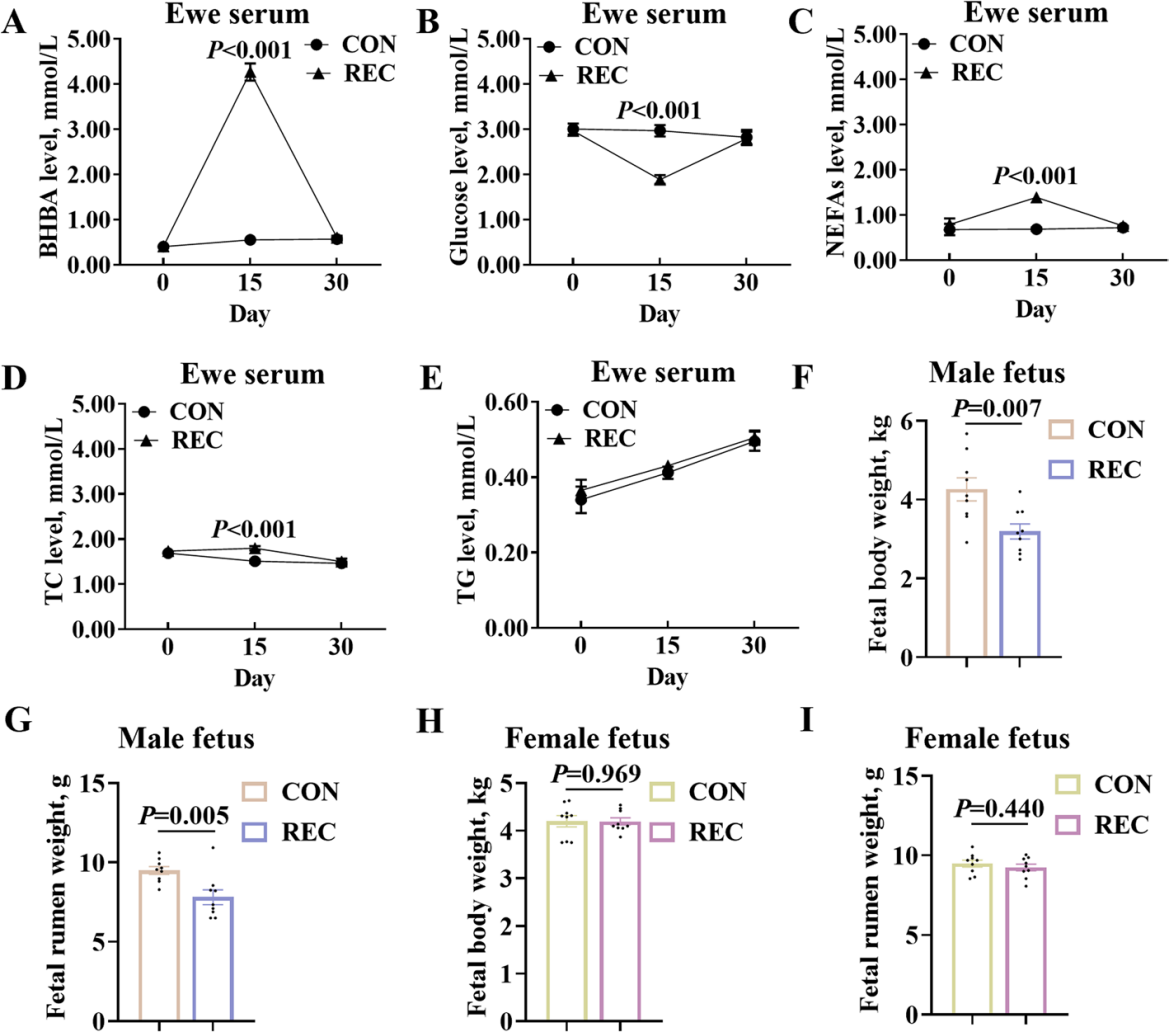
Maternal nutrition recovery restored ewe’s metabolic disorder but failed to recover male fetus’ maldevelopment. **A** BHBA, **B** glucose, **C** NEFAs, **D** TC, and **E** TG levels in nutrition-recovery ewe serum. **F** Male fetal body weight and **G** Male fetal rumen empty weight in nutrition-recovery models. **H** Female fetal body weight and **I** Female fetal rumen empty weight in nutrition-recovery models. Data were presented as mean ± SEM. Significance was assessed by two-tailed unpaired Student’s *t*-test and repeated measures ANOVA

To find out whether maternal nutritional recovery can restore undernutrition-induced ewe’s metabolic disorders and fetal rumen maldevelopment, a nutritional recovery model was established using 15-d feed restriction followed by 15-d ad libitum feeding. At trial initiation, ewe body weights showed no significant difference between the CON and REC groups (*P* = 0.229) (Table 3). At both d 15 and d 30 time points, the body weight and average daily gain of REC group ewes were significantly lower than those in the CON group (*P* < 0.001) (Table 3). From d 15 to d 30, daily weight gain and daily feed intake showed no significant differences between the CON and REC groups (*P* > 0.050) (Table 3). After 30-d treatment, the ewe’s serum BHBA, glucose, NEFAs, TC, and TG levels were no significantly different than that in the CON group (Fig. 2A–E). However, male fetal body weight (*P* = 0.007) and rumen tissue weight (*P* = 0.005) in the REC group were still significantly lighter than the CON group (Fig. 2F–G), while female fetus’ body weight (*P* = 0.969) and rumen tissue weight (*P* = 0.440) in the REC group were no significantly different than that in the CON group (Fig. 2H–I). Consequently, maternal nutritional recovery alleviated the undernutrition-induced ewe’s metabolic disorders, yet failed to ameliorate male fetal rumen maldevelopment, without significant effects on female fetal rumen.

**Table 3:** Growth performance and daily feed intake of ewes in the CON and REC groups (*n* = 12)^1^

Item	Groups^2^		*P*-value
	CON	REC	
d 0 body weight, kg	55.06 ± 0.41	54.22 ± 0.54	0.229
d 15 body weight, kg	56.70 ± 0.50^a^	45.45 ± 0.63^b^	< 0.001
d 30 body weight, kg	58.26 ± 0.63^a^	47.20 ± 0.35^b^	< 0.001
d 0–15 daily weight gain, g/d	109.44 ± 23.73^a^	−584.44 ± 37.95^b^	< 0.001
d 15–30 daily weight gain, g/d	103.89 ± 14.94	116.67 ± 32.24	0.723
d 0–30 daily weight gain, g/d	106.67 ± 18.14^a^	−233.89 ± 11.12^b^	< 0.001
d 0–15 daily feed intake, kg/d	1.63 ± 0.01^a^	0.48 ± 0.01^b^	< 0.001
d 15–30 daily feed intake, kg/d	1.66 ± 0.01	1.61 ± 0.03	0.140
d 0–30 daily feed intake, kg/d	1.65 ± 0.02^a^	0.84 ± 0.02^b^	< 0.001

^1^The data were expressed as the mean ± SEM

^2^CON, control group; REC, nutritional recovery group

^a,b ^Means with different letters are significantly different (*P* < 0.05), whereas those sharing a common letter or with no letter do not differ significantly

### Maternal undernutrition inhibited male fetal rumen development via impeding cell cycle and nutrient metabolism

**Fig. 3:**
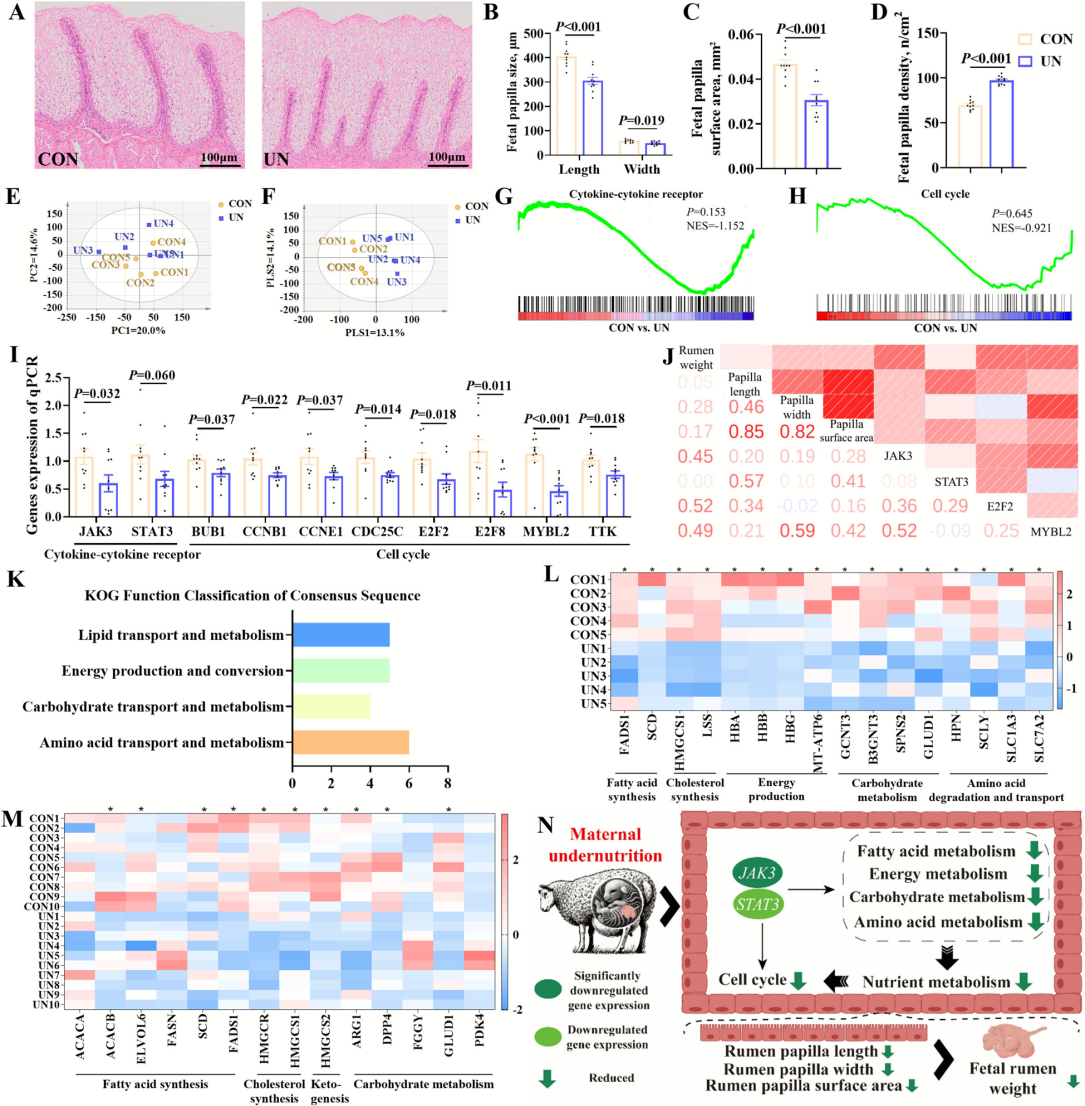
Maternal undernutrition suppressed nutrient metabolism and cell cycle in male fetal rumen to inhibit fetal rumen development. **A** Male fetal rumen hematoxylin–eosin staining sections. **B** Male fetal rumen papilla size. **C** Male fetal rumen papilla surface area. **D** Male fetal rumen papilla number. **E**–**F** PCA and PLS-DA of total gene expression in rumen of the CON and UN groups. PCA score scatter plot (predictive ability parameter (Q2) (cum) = 0.200, goodness-of-fit parameter (R2) (Y) = 0.146). PLS-DA score scatter plot (predictive ability parameter (Q2) (cum) = 0.131, goodness-of-fit parameter (R2) (Y) = 0.141). **G**–**H** GSEA maps of enriched pathways associated with cytokine-cytokine receptor and cell cycle. **I** The expression of genes related to cytokine-cytokine receptor and cell cycle by RT-qPCR. **J** Correlation analysis of rumen phenotypes and gene expression of cytokine-cytokine receptor and cell cycle. **K** KOG functional classification of DEGs in fetal rumen between the CON and UN groups. **L** The expression of DEGs related to nutrient metabolism by RNA-Seq. **M** The expression of genes related to nutrient metabolism by RT-qPCR. **N** Maternal undernutrition inhibited fetal rumen growth and development by reducing nutrient metabolism, cytokines and cytokine receptors, and cell cycle. Rumen weight, papilla size, papilla surface area, papilla density, and RT-qPCR date were analyzed using two-tailed *t*-test (*n* = 10, ^*^P < 0.05)

Maternal undernutrition caused a significant reduction in the length (*P* < 0.001), width (*P* = 0.019), surface area (*P* < 0.001) (Fig. 3A–C), while a significant increase in the density of male fetal rumen papilla was observed (Fig. 3D). Transcriptome analysis revealed the clear distinctions of the general transcriptional profiles of male fetal rumen between the CON and UN groups, as shown in PCA and PLS-DA plots of total genes (Fig. 3E–F). The volcano plot analysis identified 38 upregulated genes, 111 downregulated genes, and 15,771 non-significant genes (Fig. S1A). Further GSEA identified the enrichment of genes involved in cytokine-cytokine receptor interaction (*P* = 0.153, NES = −1.152) and the cell cycle (*P* = 0.645, NES = −0.921) (Fig. 3G and H). RT-qPCR results also demonstrated the downregulation of cytokine-cytokine receptor-related genes such as *JAK3* (*P* = 0.032) and *STAT3* (*P* = 0.060) and cell cycle-related genes including *BUB1* (*P* = 0.037), *CCNB1* (*P* = 0.022), *CCNE1* (*P* = 0.037), *CDC25C* (*P* = 0.014), *E2F2* (*P* = 0.018), *E2F8* (*P* = 0.011), *MYBL2* (*P* = 0.037), and *TTK* (*P* = 0.018) (Fig. 3I). Additionally, male fetal rumen weight and the length, width, and surface area of rumen papilla were positively correlated with the expression levels of genes related to JAK3/STAT3 signaling pathway and cell cycle (Fig. 3J). KOG classification revealed that lipid, carbohydrate, and amino acid transport and metabolism, as well as energy production and conversion, were notably enriched by DEGs between the CON and UN groups (Fig. 3K). DEGs associated with fatty acid and cholesterol synthesis as well as energy, carbohydrate, and amino acid metabolism were all downregulated (Fig. 3L). RT-qPCR results confirmed the transcriptome findings, showing downregulation of genes related to fatty acid synthesis (*ACACA*, *ACACB*, *ELVOL6*, *FASN*, *SCD*, and *FADS1*), cholesterol synthesis (*HMGCR* and *HMGCS1*), ketogenesis (*HMGCS2*), and carbohydrate metabolism (*ARG1*, *DPP4*, *FGGY*, *GLUD1*, and *PDK4*) (Fig. 3M). Meanwhile, gene expression levels of *JAK3* and *STAT3* were positively correlated with expression levels of genes related to nutrient metabolism including fatty acid and cholesterol synthesis as well as carbohydrate metabolism (Fig. S1B). Thus, maternal undernutrition inhibited nutrient metabolism and cell cycle via JAK3/STAT3 signaling pathway, which suppressed male fetal rumen development (Fig. 3N).

### Maternal nutritional recovery partially restored undernutrition-disrupted metabolism but failed to alleviate male fetal rumen maldevelopment

**Fig. 4:**
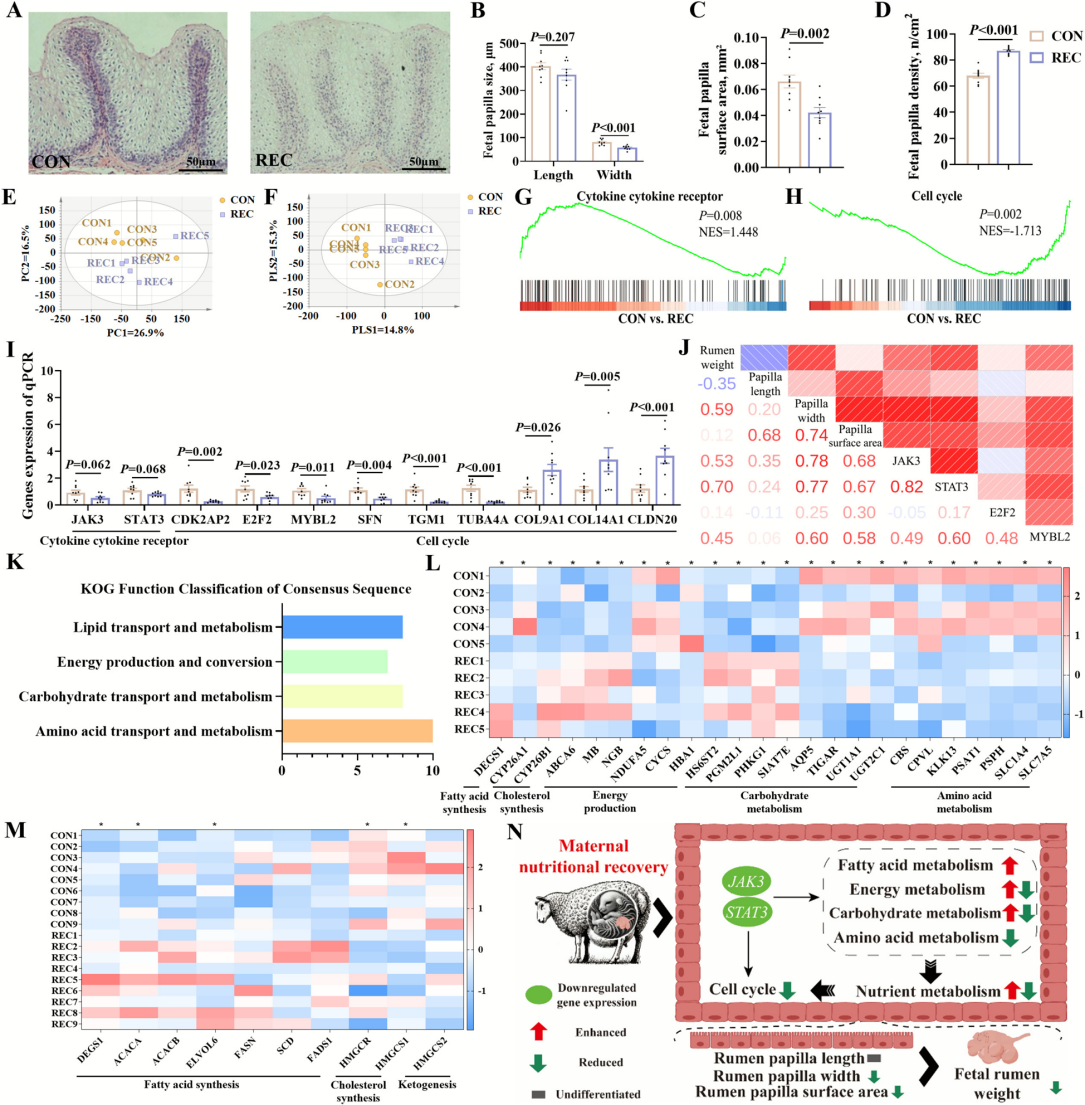
Maternal nutritional recovery partially restored metabolic inhibition but failed to alleviate male fetal rumen development. **A** Male fetal rumen hematoxylin–eosin staining sections. **B** Male fetal rumen papilla size. **C** Male fetal rumen papilla surface area. **D** Male fetal rumen papilla number. **E**–**F** PCA and PLS-DA of total gene expression in rumen of the CON and REC groups. PCA score scatter plot (predictive ability parameter (Q2) (cum) = 0.269, goodness-of-fit parameter (R2) (Y) = 0.165). PLS-DA score scatter plot (predictive ability parameter (Q2) (cum) = 0.148, goodness-of-fit parameter (R2) (Y) = 0.153). **G**–**H** GSEA maps of enriched pathways associated with cytokine-cytokine receptor and cell cycle. **I** The expression of genes related to cytokine-cytokine receptor and cell cycle by RT-qPCR. **J** Correlation analysis of rumen phenotypes and gene expression of cytokine-cytokine receptor and cell cycle. **K** KOG functional classification of DEGs in fetal rumen between the CON and REC groups. **L** The expression of DEGs related to nutrient metabolism by RNA-Seq. **M** The expression of genes related to nutrient metabolism by RT-qPCR. **N** Maternal nutritional recovery partially restored metabolic inhibition to alleviate undernutrition-induced fetal rumen maldevelopment. Rumen weight, papilla size, papilla surface area, papilla density, and RT-qPCR date were analyzed using two-tailed *t*-test (*n* = 9, ^*^P < 0.05)

Male fetal rumen papillary width (*P* < 0.001) and surface area (*P* = 0.002) in the REC group were significantly lower than the CON group while male fetal rumen papillary length was almost recovered (*P* = 0.207) (Fig. 4A–C). Interestingly, the density of fetal rumen papilla in the REC group was still significantly higher than that in the CON group (Fig. 4D). Transcriptome analysis of the male fetal rumen still revealed clear distinctions between the CON and REC groups, as shown in PCA and PLS-DA plots of total genes (Fig. 4E–F). The volcano plot analysis identified 159 upregulated genes, 82 downregulated genes, and 14,971 non-significant genes (Fig. S1C). Further, GSEA identified significant enrichment of genes involved in cytokine-cytokine receptor interaction (*P* = 0.008, NES = 1.448) and the cell cycle (*P* = 0.002, NES = −1.713) (Fig. 4G–H). RT-qPCR results demonstrated the downregulation of cell cycle-related genes including *CDK2AP2* (*P* = 0.002), *E2F2* (*P* = 0.023), *MYBL2* (*P* = 0.011), *SFN* (*P* = 0.004), *TGM1* (*P* < 0.001), and *TUBA4A* (*P* < 0.001) and the upregulation of cell cycle-related genes including *COL9A1* (*P* = 0.026), *COL14A1* (*P* = 0.005), and *CLDN20* (*P* < 0.001) in male fetal rumen of REC group. Moreover, the expression of *JAK3* (*P* = 0.062) and *STAT3* (*P* = 0.068) also showed downregulated trend (Fig. 4I). Additionally, male fetal rumen weight and the length, width, and surface area of rumen papilla were positively correlated with expression levels of key genes related to JAK3/STAT3 signaling pathway and cell cycle (Fig. 4J). KOG classification revealed that lipid, carbohydrate, and amino acid transport and metabolism and energy production and conversion, were also notably enriched by DEGs between the CON and REC groups (Fig. 4K). DEGs associated with cholesterol synthesis, energy metabolism, carbohydrate metabolism, and fatty acid synthesis were partially upregulated in male fetal rumen of REC group, while DEGs related to amino acid metabolism were still downregulated (Fig. 4L). RT-qPCR analysis revealed that maternal nutritional recovery after nutritional restriction upregulated genes associated with fatty acid synthesis (*DEGS1*,* ACACA*, and *ELVOL6*) and downregulated genes related to cholesterol synthesis (*HMGCR* and *HMGCS1*) in male fetal rumen (Fig. 4M). Meanwhile, gene expression levels of *JAK3* and *STAT3* were negatively correlated with expression level of genes related to fatty acid synthesis and positively correlated with expression level of genes related to cholesterol synthesis (Fig. S1D). Thus, maternal nutritional recovery could partially alleviate the inhibited nutrient metabolism in male fetal rumen caused by maternal undernutrition, while the suppressed cell cycle and the retarded male fetal rumen development still existed (Fig. 4N).

### Maternal undernutrition altered male fetal rumen miRNA profile and upregulated novel miRNA-736 expression

**Fig. 5:**
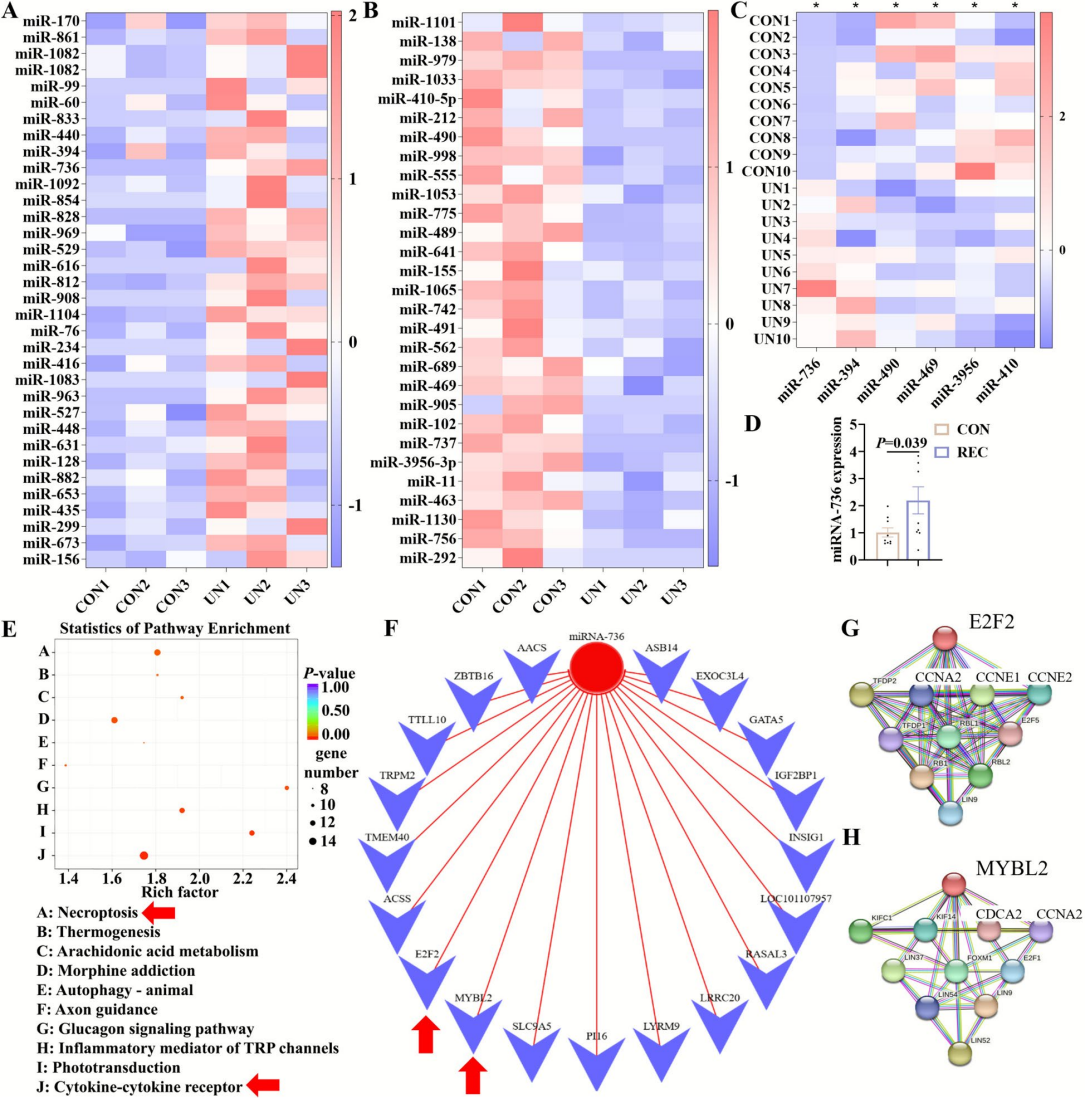
Differentially expressed miRNA analysis in male fetal rumen induced by maternal undernutrition. **A**–**B** DEMs in male fetal rumen of maternal undernutrition model (*n* = 3). **C** DEMs verification by RT-qPCR. **D** Novel miRNA-736 expression level in male fetal rumen of maternal nutrition-recovery model via RT-qPCR. **E** KEGG analysis of DEMs between the CON and UN groups. **F** Network map of novel miRNA-736 target genes. **G**–**H** Protein–protein interaction networks of E2F2 and MYBL2 constructed using STRING database. RT-qPCR data were analyzed using two-tailed *t*-test (*n* = 10, ^*^P < 0.05)

It was hypothesized that miRNA might play a role in the epigenetic regulation of male fetal rumen plasticity during pregnancy. In the analysis of miRNAs expressional profile, 1,253 miRNAs were detected including 152 known miRNAs and 1,101 novel miRNAs (Table S4). To investigate the clustering of samples, PCA of total miRNA read counts in male fetal rumen was performed. PCA plot showed clear distinction between the CON and UN groups, which indicated the changed male fetal rumen miRNA profile upon maternal undernutrition (Fig. S2A). Compared to the CON group, 34 DEMs were upregulated (Fig. 5A), while 29 DEMs were downregulated in male fetal rumen of UN group (Fig. 5B). RT-qPCR results confirmed the findings from miRNA sequencing (Fig. 5C). Excitingly, the significant upregulation of miR-736 (*P* = 0.039) was also found in male fetal rumen of ewes from the REC group compared to the gestational-age-matched controls (Fig. 5D). Novel miR-736 was previously not found in the development of ruminant animals and had unique sequence (The sequence included 5'-UGGGCCUGUGGUACCCCCCCCAUC-3'). To further explore the functions of these putative miRNA gene targets, KEGG enrichment analysis was conducted in which necroptosis and cytokine-cytokine receptor interaction were significantly enriched (Fig. 5E). Interestingly, *E2F2* and *MYBL2* were the intersection of DEGs dataset in male fetal rumen induced by maternal undernutrition, DEGs dataset in male fetal rumen induced by maternal nutritional recovery, and DEMs target genes dataset (Fig. S2B). Notably, miR-736 was found to target *E2F2* and *MYBL2* (Fig. 5F). STRING analysis indicated that *E2F2* and *MYBL2*, the potential target genes of miR-736, were closely associated with cell cycle-related genes, including *CCNA2*, *CCNE1*, *CCNE2*, and *CCNA2* (Fig. 5G–H). GO enrichment analysis further revealed that proteins interacting with *E2F2* and *MYBL2* were significantly enriched in cell cycle-related processes (Fig. S2C–D). Thus, maternal undernutrition might inhibit male fetal rumen development by upregulating miR-736, which then downregulated the expression of its target genes, *E2F2* and *MYBL2*, ultimately leading to the suppression of cell cycle-related gene expression.

### Novel miRNA-736 targeted *E2F2* and *MYBL2* and regulated rumen epithelial cell development via cell cycle and apoptosis

**Fig. 6:**
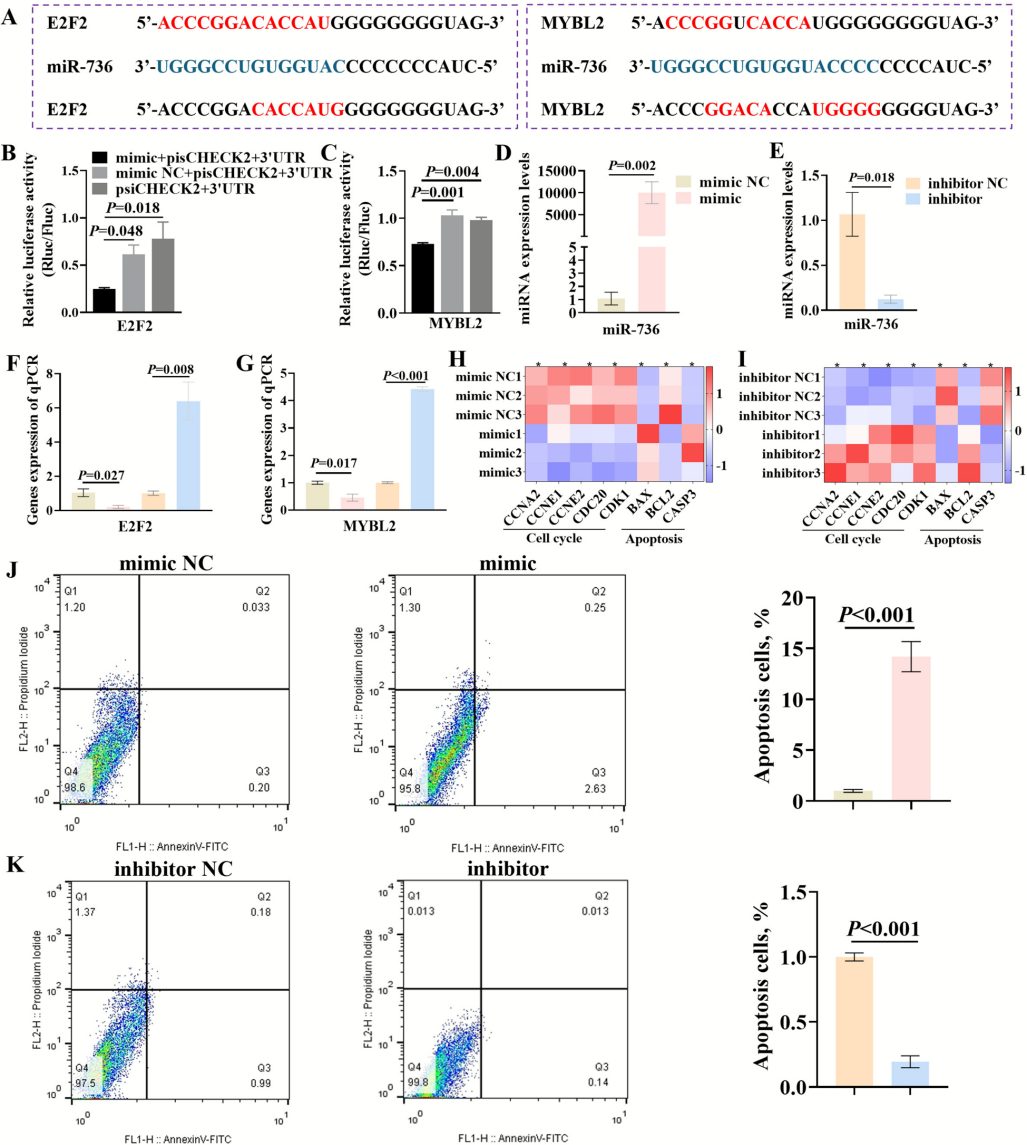
Novel miRNA-736 regulated rumen epithelial cell cycle and apoptosis by targeting *E2F2* and *MYBL2*. **A** Novel miRNA-736 target sites prediction for *E2F2* and *MYBL2*. **B**–**C** Verification of the presence of novel miRNA-736 binding site in the *E2F2* and *MYBL2* 3' UTR by a dual-luciferase reporter assay in 293 T cells. **D**–**E** Transfection efficiency of novel miRNA-736 mimic and inhibitor in Hu-sheep rumen epithelial cells via RT-qPCR. **F**–**G** The mRNA expression levels of *E2F2* and *MYBL2* transfected with novel miRNA-736 mimic and inhibitor. **H**–**I** The expression of genes related to cell cycle and apoptosis transfected with novel miRNA-736 mimic and inhibitor. **J**–**K** Effects of novel miRNA-736 mimic and inhibitor transfection on rumen epithelial cell apoptosis. mimic NC or inhibitor NC, negative control; mimic, overexpression of novel miRNA-736; inhibitor, inhibition of novel miRNA-736. pisCHECK2 + 3' UTR = dual-luciferase reporter recombinant vectors *E2F2* or *MYBL2* -wt-3' UTR. Rluc/Fluc = Renilla luciferase/firefly luciferase. The data were analyzed using two-tailed *t*-test for comparisons between two groups (*n* = 3) or one-way ANOVA for comparisons among three groups (*n* = 3), ^*^P < 0.05

The binding sites of novel miRNA-736 to the 3' UTR regions of *E2F2* and *MYBL2* were predicted using miRanda software (Fig. 6A). To further validate *E2F2* and *MYBL2* were target genes of miR-736, the 3' UTRs of *E2F2* and *MYBL2* were cloned into the psiCHECK2 vector, respectively. The inserted fragment lengths of these two recombinant vectors were verified using *XhoI*-*ApaI* double enzyme digestion and gel electrophoresis and the inserted fragments sequences were confirmed by DNA sequencing (Fig. S3A–B). The psiCHECK2 + 3' UTR constructs and miR-736 mimics (or negative control mimic NC) were co-transfected into HEK-293 cells, and luciferase activity was measured using a dual luciferase reporter assay. The results showed that the luciferase activity in the co-transfected psiCHECK2 + 3' UTR (*E2F2* or *MYBL2*) and miR-736 mimic group was significantly downregulated compared to the negative control groups, confirming that novel miRNA-736 targeted both *E2F2* and *MYBL2* (Fig. 6B–C). To explore the regulatory function of miR-736 in rumen epithelial cells, overexpression and silencing experiments were performed using miR-736 mimics and inhibitors, respectively. RT-qPCR results demonstrated that miR-736 mimics significantly increased the expression of mature miR-736 in rumen epithelial cells (*P* = 0.002) (Fig. 6D), while miR-736 inhibitors significantly decreased its expression level (*P* = 0.018) (Fig. 6E). Overexpression of miR-736 resulted in a significant downregulation of *E2F2* (*P* = 0.027) and *MYBL2* (*P* = 0.017) gene expression in rumen epithelial cells, while inhibition of miR-736 caused a significant upregulation of *E2F2* (*P* = 0.008) and *MYBL2* (*P* < 0.001) gene expression (Fig. 6F–G). Additionally, overexpression of miR-736 significantly reduced the expression of cell cycle-related genes (*CCNA2*, *CCNE1*, *CCNE2*, and *CDC20*) and the anti-apoptosis-related gene (*BCL2*), while upregulating apoptosis-related genes (*BAX* and *CASP3*) in rumen epithelial cells (Fig. 6H). Conversely, inhibition of miR-736 produced the opposite effects on the expression of genes associated with cell cycle and apoptosis (Fig. 6I). Flow cytometry analysis revealed that overexpression of miR-736 significantly increased the apoptosis level of rumen epithelial cells (*P* < 0.001) compared to the mimic NC group, while inhibition of miR-736 significantly decreased the apoptosis level (*P* < 0.001) (Fig. 6J–K). Thus, novel miRNA-736 targeted *E2F2* and *MYBL2* and acted as a negative regulator of male fetal rumen development by inhibiting cell cycle progression and promoting apoptosis.

### Silencing of *E2F2* and *MYBL2* inhibited cell cycle and promoted apoptosis of rumen epithelial cells

**Fig. 7:**
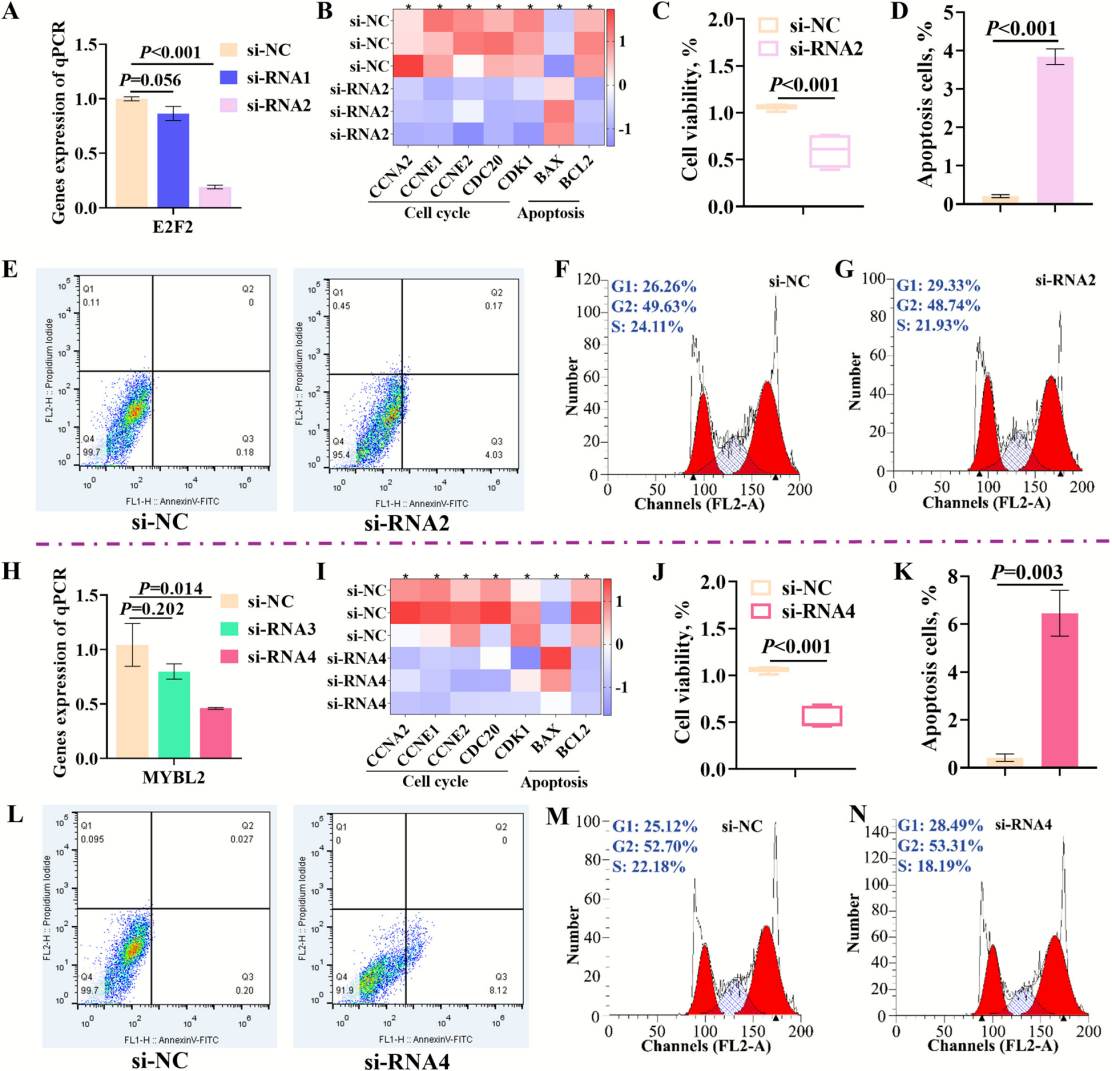
*E2F2* and *MYBL2* regulated rumen epithelial cell cycle and apoptosis. **A** *E2F2* silencing efficiency assay. **B** The expression of genes related to cell cycle and apoptosis transfected with siRNA-*E2F2*. **C** Detection of rumen epithelial cell viability transfected with siRNA-*E2F2*. **D**–**E** Detection of rumen epithelial cell apoptosis rate transfected with siRNA-*E2F2*. **F**–**G** Cell cycle detection of rumen epithelial cells transfected with siRNA-*E2F2*. **H** MYBL2 silencing efficiency assay. **I** The expression of genes related to cell cycle and apoptosis transfected with siRNA-*MYBL2*. **J** Detection of rumen epithelial cell viability transfected with siRNA-*MYBL2*. **K**–**L** Detection of rumen epithelial cell apoptosis rate transfected with siRNA-*MYBL2*. **M**–**N** Cell cycle detection of rumen epithelial cells transfected with siRNA-*MYBL2.* The data were analyzed using two-tailed *t*-test for comparisons between two groups (*n* = 3) or one-way ANOVA for comparisons among three groups (*n* = 3), ^*^P < 0.05

To further confirm that miR-736 regulated the development of rumen epithelial cells by targeting the inhibition of *E2F2* and *MYBL2*, *E2F2* and *MYBL2* were silenced in rumen epithelial cells to examine their effects on the cell cycle and apoptosis. The results showed that the transfection of siRNA2 resulted in an 80.85% reduction in *E2F2* gene expression in rumen epithelial cells (Fig. 7A), so siRNA2 was used for the following assays. Silencing of *E2F2* significantly reduced the mRNA expression of cell cycle-related genes (*CCNA2*, *CCNE1*, *CCNE2*, and *CDC20*) and the anti-apoptosis-related gene *BCL2*, while upregulated the apoptosis-related gene *BAX* in rumen epithelial cells (Fig. 7B). Further analysis revealed that silencing *E2F2* expression significantly reduced the cell viability of rumen epithelial cells (Fig. 7C). Flow cytometry analysis showed that silencing *E2F2* significantly increased the apoptosis level (*P* < 0.001) of rumen epithelial cells (Fig. 7D–E), which was consistent with the results observed following miR-736 overexpression. Silencing *E2F2* significantly inhibited cell proliferation in rumen epithelial cells and negatively regulated the entry into the S-phase of the cell cycle (Fig. 7F–G). Additionally, silencing *MYBL2* by transfecting siRNA4 resulted in a 53.97% reduction in *MYBL2* gene expression in rumen epithelial cells (Fig. 7H). Silencing *MYBL2* also significantly inhibited the mRNA expression of cell cycle-related genes (*CCNA2*, *CCNE1*, *CCNE2*, and *CDC20*) and the anti-apoptosis-related gene *BCL2*, while upregulated the apoptosis-related gene *BAX* in rumen epithelial cells (Fig. 7I). Moreover, silencing *MYBL2* significantly reduced the cell viability of rumen epithelial cells (Fig. 7J). Flow cytometry analysis showed that silencing *MYBL2* also significantly increased the apoptosis level (*P* < 0.001) (Fig. 7K–L) and negatively regulated entry into the S-phase of the cell cycle (Fig. 7M–N). Thus, maternal undernutrition induced the overexpression of novel miRNA-736 in male fetal rumen, which targeted *E2F2* and *MYBL2*, leading to enhanced apoptosis and the negative regulation of S-phase entry in rumen epithelial cells, ultimately inhibiting male fetal rumen development.

## Discussion

Rumen development during early life critically determines lifelong nutrient utilization in ruminants [23]. Maternal nutritional status, especially for multiple-fetus characteristics induced undernutrition in Hu-sheep during late gestation, profoundly influences fetal development [24, 25]. However, the effect of maternal undernutrition on the metabolic homeostasis and development of fetal rumen is poorly understood. Dissecting the phenotypes and molecular regulatory mechanisms of maternal undernutrition-affected fetal rumen will enrich the mother-child nutrition theory and contribute to finding novel strategies to promote fetal development in utero and improve productivity after birth.

Previous studies showed that fetal small intestinal and total gastrointestinal tract mass [26] and liver mass [27] were decreased by mid-gestation feed restriction in ewes. In the current study, maternal undernutrition significantly reduced male fetal rumen weight which was consistent with a previous report about the effects of maternal nutritional restriction on fetal rumen development in cows during pregnancy [28]. Furthermore, maternal undernutrition reduced the length, width, and surface area of ruminal papilla in male fetal rumen. Interestingly, nutritional recovery partially restored male fetal rumen development reduced by previous maternal undernutrition; however, male fetal rumen weight and the width and surface area of rumen papilla were still lower than the normal levels. In addition, the number of rumen papillae in male fetuses of undernourished ewes was higher than that in the fetuses of nutritional recovery ewes, indicating that a higher density of fetal rumen papilla was likely a strategy to confront the lack of nutrient supply.

Ewe undernutrition during late gestation affected lipid, carbohydrate, amino acid, and energy metabolism in the rumen [12], jejunum and ileum [22], cecum [29], pituitary [30], fat [31], and liver [2]. In this study, maternal undernutrition extremely reduced nutrient metabolism including fatty acid synthesis, cholesterol synthesis, carbohydrate metabolism, amino acid metabolism, and energy production in male fetal rumen. It was worthy to note that lipid metabolism and transport were closely linked to cell survival and growth, since the inhibition of lipid metabolism induced a suppression of cell proliferation [32, 33]. In addition, carbohydrate, amino acid, and energy metabolism played important roles in rumen development [34, 35]. Systemic inhibition of JAK/STAT signaling pathway induced multi-organ metabolic dysregulation [36]. Hepatic *STAT5* deficiency exacerbated hyperglycemia and dyslipidemia [37] and liver-specific *STAT6* deletion directly mediated hepatic steatosis and insulin resistance [38]. Adipose *STAT3*/*STAT5* ablation promoted obesity and suppresses lipolytic capacity [39, 40]. Skeletal muscle *STAT5* depletion reduced lean mass and impaired glucose tolerance [41]. In this study, *JAK3* and *STAT3* expression levels were significantly correlated with the expression level of genes related to nutrient metabolism and energy production. Therefore, maternal undernutrition suppressed nutrient metabolism and energy production via JAK3/STAT3 signaling pathway and further inhibited male fetal rumen development, while maternal nutritional recovery only partially restored metabolic homeostasis to alleviate undernutrition-induced male fetal rumen maldevelopment.

Metabolic disorders and energy production barriers could affect cell cycle and proliferation. Notably, *CCNB1* played a crucial role in cell division and had been shown to accelerate the cell cycle and then promote rumen growth in goats [42]. The enhancement of rumen cell proliferation was associated with the increased expression of *CCNB1* and *CCNE1*, which contributed to the development of rumen epithelium [42]. In addition, BUB1 played an active role in promoting the proliferation of Human endometrial epithelial cells and endometrial cancer Ishikawa cells [43]. Interestingly, E2F family proteins were transcriptional activators of the genes encoding cell cycle-regulatory proteins including CCNA, CCNE, CDK2, and CDC25 [44]. In this study, it was found maternal undernutrition downregulated these genes related to cell cycle and cell proliferation in male fetal rumen and inhibited male fetal rumen development.

As a critical post-transcriptional regulatory mechanism, miRNAs play a much larger role in gene expression regulation than previously understood [45, 46]. Although miRNAs had been widely identified in various ruminant tissues, their roles in regulating gastrointestinal development and function remain underexplored [47]. In calves, miR-143 was the most highly expressed miRNA in rumen, and its expression pattern changed over the first 6 weeks after birth [48]. The targeted genes of miR-143 were associated with immune response and developmental processes [48]. In this study, we identified a total of 1,253 miRNAs in male fetal rumen, including 1,101 newly predicted miRNAs and 152 known miRNAs. Moreover, we found the newly identified miR-736 targeted *E2F2* and *MYBL2*. In mouse lung cancer cells, miR-637 targeted *E2F2* and reduced cell proliferation [49]. However, to date, no *E2F2*-targeting miRNAs had been reported in the rumen. In addition, *MYBL2* expression was downregulated during the maturation of colon epithelial cells, in part through miR-365 [50]. In this study, it was confirmed for the first time that the newly identified novel miRNA-736 inhibited *E2F2* and *MYBL2*, thereby promoted apoptosis and negatively regulated entry into the S-phase of the cell cycle for rumen epithelial cells. Therefore, maternal undernutrition induced the overexpression of novel miRNA-736 in male fetal rumen, which targeted *E2F2* and *MYBL2*, promoted rumen epithelial cell apoptosis, and inhibited cell cycle and cell proliferation.

The oncogenic effects of E2F family genes (*E2F1* and *E2F7*) in gastric cancer were at least partially mediated through transcriptional activation of *MYBL2* [51]. *E2F1*, *E2F2*, and *E2F3* were transcriptional activators while *E2F7* and *E2F8* acted as atypical transcriptional suppressors [52]. *E2F2* was closely involved in several critical cellular processes including cell cycle, apoptosis, differentiation, and stress response [53]. Silencing *E2F2* had been shown to impair cell growth, invasion, and migration in gastric cancer cells [54]. In this study, silencing *E2F2* significantly inhibited the proliferation of rumen epithelial cells and promoted apoptosis. In addition, knockdown of *MYBL2* significantly inhibited the proliferation and migration of ovarian cancer cells [55]. In our study, silencing *MYBL2* also significantly inhibited cell proliferation and promoted apoptosis of rumen epithelial cells. Therefore, maternal undernutrition downregulated *E2F2* and *MYBL2* in male fetal rumen, which further suppressed cell cycle and cell proliferation, promoted cell apoptosis, and inhibited male fetal rumen development.

## Conclusions

**Fig. 8:**
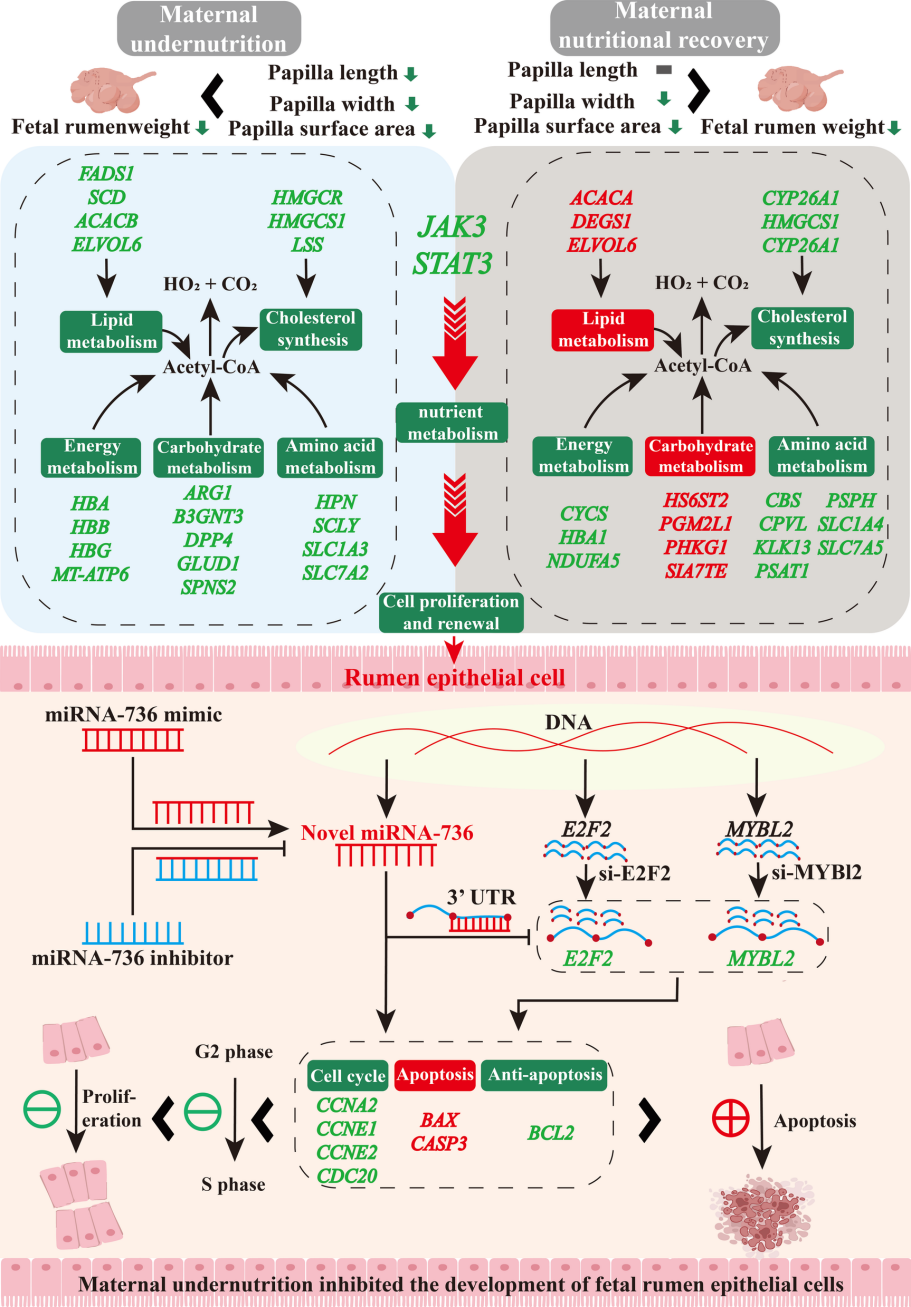
Mechanism summary diagram of maternal undernutrition induced fetal rumen nutrient metabolism disorders and development retardation. Red font and background indicated enhancement, green font and background indicated decrease

In conclusion, maternal undernutrition suppressed nutrient metabolism and energy production via JAK3/STAT3 signaling pathway and further inhibited male fetal rumen development, while maternal nutritional recovery partially restored metabolic homeostasis and energy production to alleviate undernutrition-induced male fetal rumen maldevelopment (Fig. 8). Interestingly, maternal undernutrition induced the overexpression of novel miRNA-736 in male fetal rumen, which inhibited the expression of its target genes *E2F2* and *MYBL2*, thereby disrupted cell cycle, promoted apoptosis, and ultimately inhibited male fetal rumen development (Fig. 8). However, this study had certain limitations. It was still unknown why maternal undernutrition and nutritional recovery didn’t affect the rumen development of female fetuses. Therefore, these findings provided valuable insights into the molecular mechanisms underlying the impact of maternal nutrition on fetal growth and development and contributed to developing potential nutritional strategies to improve fetal health outcomes.

## Supplementary Information

Additional file 1: Table S1. si-NC and si-RNA primer sequence. Table S2. 3 'UTR terminal primer sequence. Table S3. Primer sequence of RT-qPCR. Table S4. The sequences of all miRNAs in the rumen of male fetuses under maternal undernutrition

Additional file 2: Fig. S1. The volcano plots and correlation analysis in undernourished and nutrition-recovery models. Fig. S2. Functional analysis of miRNA and its target genes in fetal rumen. Fig. S3. Construction of 3' UTR vectors for E2F2 and MYBL2

## Data Availability

The raw sequencing data supporting this study have been deposited in the Gene Expression Omnibus (GEO) under controlled access, with rumen transcriptome data available through accession number GSE284288 (security token: qdqbggywvpehjif; direct link: https://www.ncbi.nlm.nih.gov/geo/query/acc.cgi?acc=GSE284288&token=qdqbggywvpehjif) and rumen miRNA sequencing data accessible via accession number GSE285445 (security token: wlqxqkmwbtoxtml; direct link: https://www.ncbi.nlm.nih.gov/geo/query/acc.cgi?acc=GSE285445&token=wlqxqkmwbtoxtml).

## Abbreviations

BHBA: *β*-Hydroxybutyrate
DEGs: Differentially expressed genes
DEMs: Differentially expressed miRNAs
E2F2: E2F transcription factor 2
FC: Fold change
MYBL2: MYB proto-oncogene like 2
NC: Negative control
NEFAs: Non-esterified fatty acids
PCA: Principal component analysis
PI: Propidium iodide
PLS-DA: Partial least squares discriminant analysis
PS: Phosphatidylserine
TC: Total cholesterol
TG: Triglyceride
