# Supplementary material for: Maternal undernutrition inhibits fetal rumen development: novel miRNA-736-mediated dual targeting of E2F2 and MYBL2 in sheep
Source: J Anim Sci Biotechnol. 2026 Jan 6;17:2. doi: 10.1186/s40104-025-01321-7 (PMC12771908; doi:10.1186/s40104-025-01321-7)
Supplement: Supplementary file 1 — Additional file 1: Table S1. si-NC and si-RNA primer sequence. Table S2. 3 'UTR terminal primer sequence. Table S3. Primer sequence of RT-qPCR. Table S4. The sequences of all miRNAs in the rumen of male fetuses under maternal undernutrition [file 40104_2025_1321_MOESM1_ESM.docx]

**Table S1.** si-NC and si-RNA primer sequence

| Name | Primer sequence (5 '→3') |
| --- | --- |
| si-NC-Sense | UUCUCCGAACGUGUCACGUTT |
| si-NC-Antisense | ACGUGACACGUUCGGAGAATT |
| si-RNA1 (si-E2F2) -Sense | GCAAGAAGGCCAAGAACAATT |
| si-RNA1 (si-E2F2) -Antisense | UUGUUCUUGGCCUUCUUGCTT |
| si-RNA2 (si-E2F2) -Sense | UGACAGAGGACAAGGCCAATT |
| si-RNA2 (si-E2F2) -Antisense | UUGGCCUUGUCCUCUGUCATT |
| si-RNA3 (si-MYBL2) -Sense | CCGAGAAGCAGAAGAGGAATT |
| si-RNA3 (si- MYBL2) -Antisense | UUCCUCUUCUGCUUCUCGGTT |
| si-RNA4 (si-MYBL2) -Sense | CCUCGGAAGGCCAGGGAAATT |
| si-RNA4 (si- MYBL2) -Antisense | UUUCCCUGGCCUUCCGAGGTT |

**Table S2.** 3 'UTR terminal primer sequence

| Name | Primer sequence (5 '→3') |
| --- | --- |
| *MYBL2*-WT | F: *ccg*TTACAGGGGGCTGGGGGCCAGTTCT  R: *attt*AGGTCCCCTGTCCTAAACAGGGGT |
| *E2F2*-WT | F: *ccg*TGGTGTGGCTTAAAGCATTTGA  R: *attt*CAAGGTTTGTCATTTAATGCCCA |

**Table S3.** Primer sequence of RT-qPCR

| Name | Primer sequence |
| --- | --- |
| miR-736 | F: GGGCCTGTGGTACCCCC |
|  | R: AGTGCAGGGTCCGAGGTATT |
| miR-490 | F: TGCTGCACCGGGGTGT |
|  | R: AGTGCAGGGTCCGAGGTATT |
| miR-469 | F: AACAAGGAGGGTTTGGGTTTG |
|  | R: GTCGTATCCAGTGCAGGGT |
| miR-394 | F: GCGGTGGACTTCCCTGGTAG |
|  | R: AGTGCAGGGTCCGAGGTATT |
| miR-410 | F: AACAATCGCCACTTTGGGTAC |
|  | R: GTCGTATCCAGTGCAGGGT |
| miR-3956 | F: AACAAGTCTGACCTTCCTGCA |
|  | R: GTCGTATCCAGTGCAGGGT |
| *JAK3* | F: AGTGACAGCAGCTCAGGAAG |
|  | R: GTCTCCTCACTTGGAGGTGC |
| *STAT3* | F: CTGAAGTTCAAAGCAGGCGG |
|  | R: GCTGGAATCAGGGGTCACAA |
| *BUB1* | F: ACGCCTCACTGAAACCCATT |
|  | R: ACGCCTCACTGAAACCCATT |
| *CCNB1* | F: CAAAGCACATGACTATCAAGAACAA |
|  | R: TCCTCAAGTGGAAAGTAGCCA |
| *CCNE1* | F: GAGAAGCCAGTGTGGCAGTC |
|  | R: AGTTCAGTACAGGCAGTGGC |
| *CDC25C* | F: TGGAGCTCCCTGTATCGCTC |
|  | R: AGTAAGGCAGCCACCTTGGAG |
| *CDK2AP2* | F: CGCTGACGTACAAAGCAACG |
|  | R: ACCTGTCTGCTTCGGATTGG |
| *E2F2* | F: AAGTCCCCGCCTCATTCTTG |
|  | R: GGTAAGTGACCCGCATGGAA |
| *E2F8* | F: CGCTGCAAGACATTGACACA |
|  | R: ACTCGGCTTTCTAGCGCTTC |
| *SFN* | F: TACAAGAACGTGGTGGGTGG |
|  | R: CGATGATGCGCTTCTTGTCG |
| *MYBL2* | F: AAAAGTGTTTCTGTGAGCGCCG |
|  | R: CTCTGCTCTGGCACATCTGAA |
| *TGM1* | F: TCACTGGCATGGATCTGCTG |
|  | R: CAGGGTGACATGATCGGAGG |
| *TTK* | F: CAGAGAGTCCTGCCTTGGTG |
|  | R: TGCCACTTAACTCCTCAGCC |
| *TUBA4A* | F: CCCGGACTCACCATGCG |
|  | R: TGGGCCATTTCGGATCTCAT |
| *ACACA* | F: ATGTTTCGGCAGTCCCTGAT |
|  | R: TGTGGACCAGCTGACCTTGA |
| *ACACB* | F: GAAGCAGGCACACATCAAGA |
|  | R: CCTGAGCAGTCTCCAAGGAC |
| *ELOVL6* | F: GCTAAGCAAAGCACCCGAA |
|  | R: CATAAACCAACCACCCCCA |
| *FASN* | F: GACCTTTCCAACAACCACCC |
|  | R: TGTTCGCTTGAGAGGCTGTA |
| *SCD* | F: GAGTACCGCTGGCACATCAA |
|  | R: CTAAGACGGCAGCCTTGGAT |
| *FADS1* | F: CTGCTGTACCTGCTGCACAT |
|  | R: GACGGACAGGTGTCCAAAGT |
| *HMGCR* | F: CCAATGGCAACAACAGAAGG |
|  | R: AGTCACAAGCACGGGGAAG |
| *HMGCS1* | F: AACATGAAGCTCCGAGAGGA |
|  | R: CTTGGAATATGCTCGGCTGT |
| *HMGCS2* | F: CCCACCCCAGTTTTTCTACA |
|  | R: ATCCTCCTGGACTGAGCAGA |
| *ARG1* | F: GGCGGAAGTCAAGAAGACTG |
|  | R: AGGTTGTCCATGCAAGTTCC |
| *DPP4* | F: GCCCGAACATTAATGCAACT |
|  | R: CAACCCAGCCAGTGGTACTT |
| *FGGY* | F: TACACCAAATCCGAGGGCTT |
|  | R: GTGCTTCGTCTGGTTGATCC |
| *GLUD1* | F: GGACGGATCTCTGCTACTGG |
|  | R: CTTCCAGTTCCTTTGGGTCA |
| *PDK4* | F: TTGCATTTTTGCGACAAGAG |
|  | R: CCCTGTGCCATTGTAGGAAT |
| *DEGS1* | F: TGTTGGCAGCCTCCTTACTT |
|  | R: GATGAGAACACAGGCTGCAA |
| *COL9A1* | F: GGAGCCACAGAGCAATGGAT |
|  | R: GTTTATTGGCACAGTGGCCG |
| *COL14A1* | F: TTCCAGTTTTCCGAGGCGAG |
|  | R: TCCCTCTTGGAGCCTTCCAT |
| *CLDN20* | F: CACTAAAGCACGCTGAAGCC |
|  | R: CCTTCAGTGTCTCGGCTTGT |

**Table S4.** The sequences of all miRNAs in the rumen of male fetuses under maternal undernutrition

| #ID | miRNA sequences | Length |
| --- | --- | --- |
| novel_miR_533 | AAAAACCCAGAUGAACUUUUU | 21 |
| novel_miR_1093 | GGGGCCUGCACACGAGCAUCAGC | 23 |
| novel_miR_74 | AAAACCAGAACGAACUUUUUG | 21 |
| novel_miR_558 | AAUUGAGGUGUAGUUAGCAAACA | 23 |
| novel_miR_184 | AUGAGGAUUUUGCUUGUUUCA | 21 |
| novel_miR_954 | UGCGAAGAAGAGGCCUGACGUGU | 23 |
| oar-miR-329a-5p | GAGGUUUUCUAGGUCUCCGUUU | 22 |
| novel_miR_129 | AAAUCCGAACGAACUUUUU | 19 |
| novel_miR_934 | UUGGGACACAGCUUCCUUGUGU | 22 |
| novel_miR_795 | CUGGUUGUACAGAUGCUUGUCU | 22 |
| novel_miR_76 | UUCCCUUUGUCAUCCUAUGCCU | 22 |
| novel_miR_660 | UUAAACAGGACUAAGGUGUUAG | 22 |
| novel_miR_652 | UAAAAGUUUGGUUGGGUUUUUC | 22 |
| novel_miR_861 | AAAAACUCAAAUGAACUUUUUG | 22 |
| novel_miR_765 | AAUUCUUUUGUGUAUUGUGGGCA | 23 |
| oar-miR-148a | UCAGUGCACUACAGAACUUUGU | 22 |
| novel_miR_668 | CAAAGAGUCGGACACAACUGAUA | 23 |
| novel_miR_1054 | CGGCUCCGUGACUCGUCCGUGG | 22 |
| novel_miR_683 | UAGGAUUUUGAAAGAUUGCUUU | 22 |
| novel_miR_372 | UCUCAACCAGACUGAUUUUGC | 21 |
| novel_miR_773 | CCUCAGUCAGCCUUGUGGAUGU | 22 |
| novel_miR_34 | UGGAGAGAACAGGUGGCUUUC | 21 |
| novel_miR_747 | AAAUCUGAACAAACUCUUUGGC | 22 |
| oar-miR-17-5p | CAAAGUGCUUACAGUGCAGGUA | 22 |
| novel_miR_112 | AAAAGUUCGUUCGGGUUUUUC | 21 |
| oar-miR-381-5p | AGCGAGGUUGCCCUUUGUAU | 20 |
| novel_miR_6 | UCUGCGUGUCCUCUGCUUUUCCG | 23 |
| novel_miR_609 | UGGGUCUUUGCGGGCGAGAUGA | 22 |
| novel_miR_44 | GCCCCUGGGCCUAUCCUAGAAC | 22 |
| novel_miR_477 | GAAAAACCUGAAUGAACUUUU | 21 |
| novel_miR_737 | GAAAACCUGAAGGAACUUUUGG | 22 |
| novel_miR_902 | UGGAAUCCUGGGAGCCAGGCUGA | 23 |
| novel_miR_874 | AAACUUGAAUGAAUGUUUUGGCC | 23 |
| novel_miR_820 | AAACCUGAAUGAACUUUUUGG | 21 |
| novel_miR_662 | CCGCAGGAAAGUGACAGGAGUU | 22 |
| novel_miR_212 | UCACAAAGAGCUGGACAUGAC | 21 |
| novel_miR_944 | AGGAACAUGGACUCUGGGCCCAGC | 24 |
| novel_miR_398 | AAACCCGAAUGAACUUUUUG | 20 |
| novel_miR_387 | AAAACCCAAGUGAACUUUUUGG | 22 |
| novel_miR_1057 | UGAUAUGUUUGAUAUUGGGUUG | 22 |
| novel_miR_1037 | UCUGGAUCAGUCUAUGCUGACUUU | 24 |
| novel_miR_462 | CUUGGCACCUAGUAAGUACUCA | 22 |
| novel_miR_960 | UCCUGUACUGAGCUGCCCCGAGG | 23 |
| novel_miR_948 | AAAACCUGAAUGAACUUUUUGG | 22 |
| novel_miR_604 | AAAAGUUCGUUCGGGUUUUUC | 21 |
| novel_miR_852 | AAAACCUGAAUGAACUUUUUGG | 22 |
| novel_miR_215 | AAAAAGUUCCUUUGGGUUUU | 20 |
| novel_miR_12 | UUACAGUUGUUCAACCAGUUACU | 23 |
| novel_miR_791 | UGACCGAUUUCUCCUGGUGUU | 21 |
| novel_miR_283 | UUGCAUAGUCACAAAAGUGAUC | 22 |
| novel_miR_783 | CAAAGUGCUCACAGUGCAGGUAGU | 24 |
| novel_miR_115 | AAAACCUGAAUGAACUUUUUGG | 22 |
| novel_miR_1006 | UAUGAAGUCCUUAAGGGGAGGGU | 23 |
| novel_miR_596 | ACGCCCUUCCCCCCCUUCUUCA | 22 |
| novel_miR_942 | AAAAUCAGAACAAACUUUUUGG | 22 |
| novel_miR_432 | AAAAACCUGAAUGAACUUUUUG | 22 |
| novel_miR_982 | UUCUUGACUGCUCACCUGGGC | 21 |
| novel_miR_222 | AAAACACGAACAAAUUUUUGGG | 22 |
| novel_miR_761 | GCGGCCCUAGGCUCUGCCAUC | 21 |
| novel_miR_111 | AAAAACCUGAAGGAACUUUUG | 21 |
| novel_miR_131 | ACCACAGGGUAGAACCACGGAC | 22 |
| novel_miR_252 | GAAAACCUGAACAAACUUUUGG | 22 |
| novel_miR_868 | UGGACGGAGAACUGAUAAGGGU | 22 |
| novel_miR_906 | UCCCGGGCUGGAGGAGUCUGCAGG | 24 |
| novel_miR_197 | CCUCUCUCGGUUAGCUCCAUAG | 22 |
| novel_miR_519 | UGGAGUGUGACAAUGGUGUUU | 21 |
| oar-miR-1193-3p | UAGGUCACCCGUUUGACUAUCC | 22 |
| oar-miR-191 | CAACGGAAUCCCAAAAGCAGCU | 22 |
| novel_miR_679 | ACCCGAGUCUGCAGACGCGUGUG | 23 |
| novel_miR_10 | AACAUUCAUUGCUGUCGGUGGGU | 23 |
| oar-miR-194 | UGUAACAGCAACUCCAUGUGGA | 22 |
| novel_miR_79 | UAUAUAUAUAUAUGUACGUAUG | 22 |
| novel_miR_981 | AGAGGACAGCUGACGCCUGUUGG | 23 |
| novel_miR_422 | UAAGGCACGCGGUGAAUGCC | 20 |
| novel_miR_22 | AAAAACCCAAAUGAACUUUUUG | 22 |
| novel_miR_991 | GCCUCCGGAGCAGCAACCCUG | 21 |
| novel_miR_686 | UCAAGGUCCGCUGUGAACACG | 21 |
| oar-miR-1185-3p | AUAUACAGAGGGAGACUCUUAU | 22 |
| oar-miR-329a-3p | AACACACCUGGUUAACCUUUUU | 22 |
| oar-miR-21 | UAGCUUAUCAGACUGAUGUUGAC | 23 |
| oar-miR-154a-3p | AAUCAUACACGGUUCACCUAUU | 22 |
| oar-miR-218a | UUGUGCUUGAUCUAACCAUGU | 21 |
| oar-miR-133 | UUGGUCCCCUUCAACCAGCUGU | 22 |
| novel_miR_471 | AAAUCUGAACAAACUUUUUGGC | 22 |
| novel_miR_650 | UGACAUCGAGGGUCCUCCGCCAGG | 24 |
| novel_miR_382 | ACUUCCGGUCUGUGAGGCAAGGC | 23 |
| novel_miR_642 | CUGCGUCUGUAGGAGCCACGA | 21 |
| novel_miR_804 | AUUGUCACCUUCUUGAGUGGA | 21 |
| novel_miR_117 | CCGCCGGGCACAGCGGCUCGCC | 22 |
| novel_miR_347 | AAAAAACCGAGUGAACUUUUUG | 22 |
| novel_miR_802 | UAGAUACUAGGAUAGAGACAA | 21 |
| novel_miR_1107 | UGGAGAGAAAGGCAGUUCCUGA | 22 |
| novel_miR_316 | UAAGAGUUCUGUAAUAACUAAC | 22 |
| novel_miR_938 | UGACUUCCCCCUGUCCACUCAGG | 23 |
| novel_miR_532 | CAAAAAGUUCGUUUGGGUUUU | 21 |
| novel_miR_377 | UUGCUUAGUCAUGUCCCACUCU | 22 |
| novel_miR_670 | AUGUGGGAUCUGGUUCCUUGA | 21 |
| novel_miR_423 | CCACGGAUGUUUGAGCAUGUGC | 22 |
| novel_miR_1105 | AUAAGACGAGCAAAAAGCUUGU | 22 |
| novel_miR_334 | GAAACUGGAACGAACUUUUUGG | 22 |
| novel_miR_640 | CCCUGGGCUCUGCCUCCC | 18 |
| novel_miR_299 | ACCUGCACGAACAGCACUUUGGA | 23 |
| novel_miR_1120 | AAAGCCUGAAUGAACUUUUGG | 21 |
| novel_miR_1034 | CCGGGCUUUAGGGAAGAAUGGGG | 23 |
| novel_miR_345 | UAACAGUCUACAGCCAUGGUCG | 22 |
| novel_miR_918 | AAAAACUCAGAUGAACUUUUUUG | 23 |
| novel_miR_582 | AAACCUGAAUAAACUUUUUGA | 21 |
| novel_miR_967 | CUCAGUCGUGUCCAACUCUUUG | 22 |
| novel_miR_726 | CAGAGUGGCAGUGUUUAGGGGUG | 23 |
| novel_miR_1102 | UAGAAAACCCAAAUGAACUUU | 21 |
| novel_miR_1122 | AAACCUGAAUGAACUUUUUGG | 21 |
| novel_miR_534 | GCUCUGACUUUAUUGCACUACU | 22 |
| novel_miR_196 | AAAACCCAAACGAACUUUUUGA | 22 |
| oar-miR-409-5p | AGGUUACCCGAGCAACUUUGCAU | 23 |
| novel_miR_182 | ACAGGCUGCUGUAGGCUU | 18 |
| novel_miR_687 | UUUCCUGGUCUGUCCUCCACAGC | 23 |
| novel_miR_465 | AAAAACCUGAACGAACUUUUU | 21 |
| novel_miR_806 | GUGUUUAGAAUGAUCUAGUGAU | 22 |
| novel_miR_911 | AAAACCCAGACGAACUUUUUG | 21 |
| novel_miR_202 | UGAAAAGUUCGUUUGGGUA | 19 |
| novel_miR_242 | UUUUGCGAUGUGUUCCUAAU | 20 |
| novel_miR_1001 | AAAACCUGAACAAACUUUUUGG | 22 |
| novel_miR_330 | UUCACUUGCUCAUCGUGUCUGACU | 24 |
| novel_miR_69 | AAAAACUUGAAUGAACUUUUUGU | 23 |
| novel_miR_786 | UAGAGCCUGGGAGCCUCAGCU | 21 |
| novel_miR_155 | CAGUCCGGUCCCGCGGUGUCUCCG | 24 |
| novel_miR_644 | AAAAAUUUGUUCGGGUUUUUCU | 22 |
| novel_miR_1085 | UUCAGCCGUGUCCUCUUUGCGA | 22 |
| novel_miR_512 | UCACUUCAGUCGUGUCCGACUCU | 23 |
| novel_miR_135 | UGAUGUUUAUCUGGAUCCUCAGA | 23 |
| novel_miR_160 | GGGCUCAGUUCAGCAGGAGA | 20 |
| novel_miR_1013 | UAUUGCACUUGUCCCGGCCUGU | 22 |
| novel_miR_38 | AAAAACCUGAACGAACUUUUUG | 22 |
| novel_miR_701 | GUGACUUCAGGCUCUAGC | 18 |
| novel_miR_1094 | UAGGCGAAAAGUUCAUUUGGAGU | 23 |
| novel_miR_457 | AAUGUACUUGUGGAGUUGGAGA | 22 |
| novel_miR_729 | AAAAACCCAAGUGAACUUUUUG | 22 |
| novel_miR_702 | AAACCUGAAUAAACUUUUUGA | 21 |
| oar-miR-106a | AAAAGUGCUUACAGUGCAGGU | 21 |
| oar-miR-654-3p | UAUGUCUGCUGACCAUCACCUUUA | 24 |
| novel_miR_651 | UACUGGCUACUCUGCAUGACC | 21 |
| oar-miR-154b-3p | AAUCAUACAUGGUUGACCUUUUU | 23 |
| novel_miR_528 | UUCAACGGGUAUUUAUUGAGC | 21 |
| novel_miR_1065 | UAUGUGUGUAUGUGUAUAUAUG | 22 |
| novel_miR_91 | AAACCUGAAUGAACUUUUUGG | 21 |
| novel_miR_748 | AAUUUUGGUUAUGUGUUUUGCC | 22 |
| novel_miR_607 | UGAUUGUCCAAACGCAAUUCUCG | 23 |
| novel_miR_1010 | AAAAAUCUGAAUGAACUUUU | 20 |
| novel_miR_58 | UGUCUGCAGGUUCUGCAUCUGU | 22 |
| novel_miR_732 | CUCAAAUCAGUGGACCUGGUAG | 22 |
| oar-miR-3958-5p | AGGUUGUCCGUGAUGUAUUUGC | 22 |
| novel_miR_896 | UGGCACCAGCACUGGCGGUG | 20 |
| novel_miR_784 | UUAUGGAAUAAGAGUUUGUAAC | 22 |
| novel_miR_349 | UCUCUUUCUAUGGUCUGUUGGCC | 23 |
| novel_miR_698 | AAAACUUGAAUGAACUUUCUGG | 22 |
| oar-miR-544-5p | UCUUGUUAAAAGGCAGAUUCU | 21 |
| novel_miR_300 | ACCCUGACGGGCGUGGAUUGUGG | 23 |
| novel_miR_1077 | UCACUGGGGGUGAAGGACC | 19 |
| novel_miR_429 | UCACUCCUCUCCUCCCGUCUUCU | 23 |
| novel_miR_153 | AAAACCUGAGCAAACUUUUUGU | 22 |
| novel_miR_62 | CAAGUCACUAGUGGUUCCGUUU | 22 |
| novel_miR_626 | AAAAAGUUCGUUUGGGUUUUUC | 22 |
| novel_miR_572 | UACAGUACUGUGAUAACUGAAG | 22 |
| novel_miR_329 | ACUGCCCCAGGUGCUGCUGGG | 21 |
| novel_miR_16 | AAAACCUGAAUGAACUUUU | 19 |
| novel_miR_309 | AGAAAAACCUGAAUGAACUUUU | 22 |
| oar-miR-409-3p | CGAAUGUUGCUCGGUGAACCCCU | 23 |
| novel_miR_691 | AAUACUGAGUUGGCCAAAGAGU | 22 |
| novel_miR_257 | CACAGGCUUAGUUGCUCCACAGC | 23 |
| novel_miR_389 | CAGAGACCUCUCAUCAUUCUUA | 22 |
| oar-miR-154b-5p | AGAGGUCUUCCAUGGUGCAUUCG | 23 |
| oar-miR-377-3p | AUCACACAAAGGCAACUUUCGU | 22 |
| novel_miR_704 | GUGUUCAUUGCAGCACUGUCUGC | 23 |
| novel_miR_438 | AAAAGUUCGUUCGGGUUUUUC | 21 |
| novel_miR_174 | UGUUGUACUUUUUUUUUUGUUC | 22 |
| novel_miR_85 | UGGGUCUUAGGCUCUGAAGGCCA | 23 |
| novel_miR_1050 | UCUCAACCAGACUGAUUUUGC | 21 |
| novel_miR_630 | ACCAUCGACCGUUGAGUGGACC | 22 |
| novel_miR_955 | UCACAGUGAACCGGUCUCUUU | 21 |
| novel_miR_265 | UGUGGGGACCUCUCAUGUCUGU | 22 |
| novel_miR_963 | AAAACCCACAUGAACUUUUG | 20 |
| novel_miR_696 | UGGAAUGUAAAGAAGUAUGUAU | 22 |
| novel_miR_1053 | CCUUAUUUCCCUGUCUCCCAGG | 22 |
| novel_miR_379 | UCCAGUCUUCUCAGAGCUCAGA | 22 |
| novel_miR_633 | CGCGACUGUCCGAAUCUUCGGG | 22 |
| novel_miR_677 | ACUGCUUCUGGAACAAAAGACU | 22 |
| novel_miR_768 | UCAGAGCUGUGGUUCAAAAGCU | 22 |
| novel_miR_927 | GAAAAGUUCGUUGGGGCUUUUC | 22 |
| novel_miR_569 | AAAAGUUCGUUCGGGUUUUU | 20 |
| novel_miR_179 | UGUGACUGCUAGAACCGCUCCUGC | 24 |
| novel_miR_470 | UCCAAUCGAACUUUUUGGCCAGC | 23 |
| novel_miR_273 | CUGGCUGACUCUGUUGCUCCUCU | 23 |
| novel_miR_565 | GAAAACCUGAACAAACUUUG | 20 |
| novel_miR_999 | AAAAGUUCGUUUGGGUUUUUCC | 22 |
| novel_miR_529 | AUGACCUAUGAAUUGACAGACA | 22 |
| novel_miR_1075 | UACCCAUUGCAUAUCGGAGCUGU | 23 |
| novel_miR_851 | UUCGGUCCUGCCUUUCCAUCCAGA | 24 |
| novel_miR_579 | AAAGCCCAAAUGAACUUUUUGA | 22 |
| novel_miR_503 | UAGAGCCUGGGAGCCUCAGCU | 21 |
| novel_miR_13 | UUCUCCCCUUCCUCCCUGCCCAGG | 24 |
| novel_miR_841 | AAAGACCCAAAUGAACUUUUUG | 22 |
| novel_miR_317 | UGGCUGUACAACUGGUUCAUUGAC | 24 |
| novel_miR_517 | AAGACCCAAACGAACUUUUUGG | 22 |
| novel_miR_492 | AAAACUGAAAUGAACUUUUUGG | 22 |
| novel_miR_865 | CAAGGAGGCUAUAGCAACAGG | 21 |
| novel_miR_646 | UGGAACCUGAAGAUAUGGAG | 20 |
| novel_miR_186 | CUGUGAGCUGUCUGUCCACCA | 21 |
| novel_miR_929 | UGACCGAUUUCUCCUGGUGUU | 21 |
| novel_miR_1115 | UUGCAUAGUCACAAAAGUGAUC | 22 |
| novel_miR_461 | UUAUUGCUUAAGAAUACGCGUAG | 23 |
| novel_miR_676 | UUUUGCGAUGUGUUCCUAAU | 20 |
| novel_miR_360 | UCCUUCAUUCCACCGGAGUCUGU | 23 |
| novel_miR_763 | AAGGUAGAUAGAACAGGUCUUG | 22 |
| novel_miR_166 | CCUCCCCUUCCUCCCUCCCUCCC | 23 |
| novel_miR_825 | AGAAGUUCGUUUGGGUUUUCUC | 22 |
| novel_miR_886 | AAACCCGAAUGAACUUUUUG | 20 |
| novel_miR_413 | UGGGCCUUUCAUCAUGGCAGCUU | 23 |
| novel_miR_827 | UUCUCAGCGUGUCCCUCCUACCCC | 24 |
| novel_miR_433 | GAAAACCCAAAUGAACUUUUUG | 22 |
| novel_miR_904 | GAAACUUGAACGAACUUUUUGG | 22 |
| novel_miR_990 | AAAAAUCUGAAUGAACUUUUUG | 22 |
| novel_miR_134 | AAAAAGUUCGUUUGGGUUUUUC | 22 |
| oar-miR-432 | UCUUGGAGUAGGUCAUUGGGUGG | 23 |
| novel_miR_469 | GAGGGUUUGGGUUUGGUCGUGGGA | 24 |
| novel_miR_418 | UAGCAGCACGUAAAUAUUGGAGU | 23 |
| novel_miR_415 | AAAACCUGAAUGAACUUUUUGG | 22 |
| novel_miR_1100 | CUUUUUGCGGUCUGGGCUUGC | 21 |
| oar-miR-374a | UUAUAAUACAACCUGAUAAGUG | 22 |
| novel_miR_207 | AAAACUGGAAUGAACUUUUUGU | 22 |
| oar-miR-496-3p | UGAGUAUUACAUGGCCAAUCU | 21 |
| novel_miR_416 | AAAAACUCAAAUGAACUUUUUG | 22 |
| novel_miR_998 | CCCAGGGACUGGGAGUAAGGUA | 22 |
| novel_miR_796 | UGUAUGCAACUCAUUGGAUGA | 21 |
| novel_miR_241 | AAACCUGAAUGAACUUUUUGG | 21 |
| novel_miR_143 | AAAAGUUCGUUCGGGUUUUUC | 21 |
| oar-miR-221 | AGCUACAUUGUCUGCUGGGUUU | 22 |
| novel_miR_678 | UGGCCACUUGCUCAGCCCGGGC | 22 |
| novel_miR_331 | UAAAGUGCUUAUAGUGCAGGUAG | 23 |
| novel_miR_879 | CUGUGCGUGUGACAGCGGCUGAU | 23 |
| novel_miR_322 | UCAGGGGCUUCAGGAGCUG | 19 |
| novel_miR_992 | UUUCCGGUUUCCAGAAGUGC | 20 |
| novel_miR_208 | AAAAACUCAAAUGAACUUUU | 20 |
| novel_miR_846 | UUAACCACUGGACUGCCCUGG | 21 |
| oar-let-7g | UGAGGUAGUAGUUUGUACAGU | 21 |
| novel_miR_493 | CAGGCGAUCUCCCACUUCCAGC | 22 |
| oar-miR-485-3p | GUCAUACACGGCUCUCCUCUCU | 22 |
| novel_miR_1020 | UUGUUGUUCACUUGCUAGGUCU | 22 |
| novel_miR_857 | GUGUGCGGAAAUGCUUCUGCUA | 22 |
| oar-miR-411b-5p | UGGUCGACCAUAAAACGUACGU | 22 |
| novel_miR_826 | AAAAACCCGAAUGAACUUUUGG | 22 |
| novel_miR_473 | UCUCUUUCUAUGGUCUGUUGGCC | 23 |
| novel_miR_476 | UACAAACCAAAAACAAAGCAGC | 22 |
| novel_miR_855 | AAAAGUUCGUUUGGGUUUUUCC | 22 |
| novel_miR_536 | CCAGGCCUAGAUGAGAAACUUU | 22 |
| novel_miR_819 | ACUUGCUGGAUGACCUCCCUGCC | 23 |
| novel_miR_978 | UCUGGCUCCGUGUCUUCACUCCC | 23 |
| oar-miR-374b | AUAUAAUACAACCUGCUAAGU | 21 |
| novel_miR_200 | AAACCUGAAUGAACUUUUUGG | 21 |
| novel_miR_356 | AAAAGUUGGUUUGAGUUUUUCU | 22 |
| oar-miR-487b-3p | AAUCGUACAGGGUCAUCCACUU | 22 |
| novel_miR_895 | AUGGAGAUGGUGCCUACUGCAU | 22 |
| oar-miR-29a | UAGCACCAUCUGAAAUCGGUU | 21 |
| novel_miR_108 | UAGAAAGUUUCUUUGGGGUUUU | 22 |
| novel_miR_723 | GCACUGAGCCUCUGGCCCCCAG | 22 |
| novel_miR_659 | AAAACCCGCAUGAACUUUUUGG | 22 |
| novel_miR_483 | GAGUGCGGGUCGGUUCUGUGC | 21 |
| novel_miR_526 | AGGGACUGAGGCGUGAGCCU | 20 |
| novel_miR_336 | UUCUGUACCACAGCCAGACUGCA | 23 |
| novel_miR_482 | AAAAACUUGAAUGAACUUUUUGU | 23 |
| novel_miR_169 | AAAGCAGGAUUUAGACUACAAU | 22 |
| novel_miR_261 | UGGCUCAGUUCAGCAGGAAC | 20 |
| novel_miR_468 | AAAACCCGGAUGAACUUUUUGG | 22 |
| novel_miR_404 | AAAACCUGAAUGAACUUAUCGG | 22 |
| novel_miR_603 | AAGGCCGAGAGGACUGAGCCCUGC | 24 |
| novel_miR_608 | UGGGGCUGGCCUGGGCCUGCG | 21 |
| novel_miR_925 | CGGCUCUGGGUCUGUGGGGAGC | 22 |
| novel_miR_464 | ACAAGUCAGGCUCUUGGGACC | 21 |
| novel_miR_913 | ACUGCUUUAAAACCUUUGACA | 21 |
| novel_miR_872 | UUGGGCAAAAAGUUCAUUUGGG | 22 |
| novel_miR_775 | AGCUGGACACGACUGAAGCGA | 21 |
| novel_miR_192 | UUUUGCAAUAUGUUCCUGAAU | 21 |
| novel_miR_1058 | UUGAAAGGGAAUGACAGCAGGGU | 23 |
| novel_miR_386 | AAAAACCCAAAUGAACUUUUUG | 22 |
| oar-miR-382-3p | AAUCAUUCACGGACAACACUU | 21 |
| novel_miR_277 | UAAGUUCGUUUGGGUUUUUCC | 21 |
| novel_miR_1123 | AAACCCGAACGAACUUUUGGGC | 22 |
| oar-miR-369-3p | AAUAAUACAUGGUUGAUCUUU | 21 |
| oar-miR-27a | UUCACAGUGGCUAAGUUCCGC | 21 |
| novel_miR_394 | GUGGACUUCCCUGGUAGCUCAGC | 23 |
| oar-miR-143 | UGAGAUGAAGCACUGUAGCUC | 21 |
| novel_miR_40 | UAGGUAGUUUCAUGUUGUUGGGA | 23 |
| novel_miR_35 | AAAACCUGAAUGAACCUUUUGU | 22 |
| novel_miR_319 | UCCGAGCCUGGGUCUCCCUCU | 21 |
| novel_miR_1042 | UUGGAAAGGCACAGGACAGG | 20 |
| novel_miR_616 | ACCAGCUGGACUGGGGACUAGGA | 23 |
| novel_miR_567 | UUCACCACCUUCUCCACCCAGC | 22 |
| oar-miR-668-3p | AUGUCACUCGGCCCACUACCC | 21 |
| novel_miR_995 | AAAAUCCAAAUGAACCUUUUGG | 22 |
| novel_miR_276 | UAUGGCUUUUUAUUCCUAUGUG | 22 |
| novel_miR_367 | AAAAAGUUCGUUUGGGUUUUUC | 22 |
| oar-miR-3957-3p | ACGCACAGCACCUCACUGAGCU | 22 |
| novel_miR_740 | AAGACCCAAACGAACUUUUU | 20 |
| novel_miR_157 | UUCAGCCGUGUCCUCUUUGCGA | 22 |
| oar-miR-369-5p | AGAUCGACCGUGUUAUAUUCG | 21 |
| novel_miR_759 | AGGGUUGGGCGGAGGCUUUCC | 21 |
| novel_miR_936 | AAACCCGAAUGAACUUUUUG | 20 |
| novel_miR_824 | CACUAGAUUGAGAGCUCCUGGA | 22 |
| novel_miR_145 | AAAACCUGAACGAACUUUUUGU | 22 |
| novel_miR_794 | AAAGGCCUGAAUGAACUUUUUG | 22 |
| novel_miR_41 | GAGAAACAUGAAUGAACUUUUGG | 23 |
| novel_miR_727 | AAAAACCUGAACGAACUUUUUG | 22 |
| novel_miR_1090 | ACACAGGUUUUGCUUCCAUCACU | 23 |
| novel_miR_863 | CUUUCAGUCGGAUGUUUACAGC | 22 |
| novel_miR_1062 | CUGUGGCCCUGCCGGUGCCUGAGC | 24 |
| novel_miR_342 | AAUCCCAUGGACAGAGAAGCC | 21 |
| novel_miR_648 | AAAACCUGAGUGAACUUUUUGG | 22 |
| novel_miR_113 | CAGGGUGAGAAUUUUGCUGGGC | 22 |
| novel_miR_436 | UCAACACUUGCUGGUUUCCUCU | 22 |
| novel_miR_634 | AAAAAUCUGAAUGAACUUUUU | 21 |
| novel_miR_279 | AUAAGUUCGUUUGGGUUUUUCC | 22 |
| novel_miR_809 | AAGCCUGAAUGAACUUUUUGG | 21 |
| novel_miR_716 | CUUCAGUCGUAUCUGACUCUGA | 22 |
| novel_miR_188 | UCUACAGUGCACGUGUCUCCAGU | 23 |
| novel_miR_843 | AAAAACCCAGAUGAACUUUUU | 21 |
| novel_miR_570 | ACAAAUCUGAAUGAAUUUUCUG | 22 |
| novel_miR_374 | AGUUGUAGACUGAAUUUCAAGU | 22 |
| novel_miR_632 | UCUUGGGCCCCACCCCUGGAGA | 22 |
| novel_miR_1111 | AGGUUGGAGUAGGGGCAUAGUGA | 23 |
| novel_miR_1033 | UCACUCAGUCGUGUCUGACUCU | 22 |
| oar-miR-323a-5p | AGGUGGUCCGUGGCGCGUUCG | 21 |
| oar-miR-412-5p | CUUCACCUGGUCCACUAGCU | 20 |
| novel_miR_553 | UACAUGGAGGUGGAUGAACUGCC | 23 |
| novel_miR_326 | UGAGACCUCCGGGUUCUGAGCU | 22 |
| novel_miR_425 | CAAAAUCCGAGCGAACUUUUU | 21 |
| novel_miR_424 | GCAUCAGCGAUCGGCGUG | 18 |
| novel_miR_1121 | AAAGCCCAAACGAACUUUUUGG | 22 |
| novel_miR_518 | ACAACACGGACUGUAGCCCACC | 22 |
| novel_miR_923 | UGUGACAGAUUGAUAACUGA | 20 |
| novel_miR_343 | AAAACUCUGAAUGAACUUUAUG | 22 |
| novel_miR_586 | UUUGCUGCUGUUUUCCUUGGAGC | 23 |
| novel_miR_51 | GAGCUACAGUGCUUCAUCUC | 20 |
| oar-miR-495-3p | AAACAAACAUGGUGCACUUCUU | 22 |
| novel_miR_204 | AAACUUGAAUGAAUGUUUUGGCC | 23 |
| novel_miR_821 | UGGAAUGUAAGGAAGUGUGUGG | 22 |
| novel_miR_897 | UCCCCGCCCGCCGGAUCUGUGGA | 23 |
| oar-miR-668-5p | GUAAGUGCGUCUCGGGUGAGC | 21 |
| novel_miR_1064 | AGGAAGCCCUGGAGGGGCUGGAGG | 24 |
| novel_miR_973 | AAAAACUAGAAUGAACUUUUU | 21 |
| novel_miR_924 | AACUUUCAGCUUAUCAUUGUGG | 22 |
| novel_miR_472 | UGUCUGCCCGCAUGCCUGCCUCU | 23 |
| novel_miR_474 | AAUAGCUCAGAAUGUCACUUCUG | 23 |
| novel_miR_1131 | GAGAGAUCAGAGGCGCAGAGU | 21 |
| novel_miR_597 | UCACUUCAGUCGUGUCCGACUCU | 23 |
| novel_miR_881 | UCACUUCAGUCGUGUCCGACU | 21 |
| novel_miR_840 | CUGAAGGCUGUCCCUGUCUCC | 21 |
| novel_miR_151 | AAAAACCCAAAUGAACUUUUUG | 22 |
| novel_miR_353 | UGCUGUGCUGGGACUUCCCUGGUC | 24 |
| oar-miR-106b | UAAAGUGCUGACAGUGCAGAU | 21 |
| novel_miR_420 | UGGCAGUGUAUUGUUAGCUGGU | 22 |
| novel_miR_808 | ACUCUCAAAGCCUUUCCAGAU | 21 |
| novel_miR_335 | CUAUGUAUGUAUUUGCUUGUUU | 22 |
| novel_miR_744 | AUGCACCUGGGCAAGGAUUCUGA | 23 |
| novel_miR_922 | GCUGGAGGCUGGGCAGGCACUGG | 23 |
| novel_miR_728 | ACAGCAGGCACAGACAGGCAGU | 22 |
| novel_miR_410 | CAUGCCUUGAGUGUAGGACCGU | 22 |
| oar-miR-376d | AUCAUAGAGGAAAAUCCACAU | 21 |
| novel_miR_943 | AACAAUAUCCUGGUGCUGAGU | 21 |
| novel_miR_637 | CAGCCUUUGUUCUCCCUGCAGG | 22 |
| oar-miR-200a | AACACUGUCUGGUAACGAU | 19 |
| novel_miR_1002 | AAACCCUGAAUGAACUUUUUGG | 22 |
| novel_miR_73 | AAACUGGAAUGAACUUUUUGGU | 22 |
| novel_miR_1086 | CAUCUAGACUGUGAGCUUCUAG | 22 |
| novel_miR_478 | AUGAACUUUUGGGCCAACCCAAU | 23 |
| novel_miR_65 | CCCAGUGUUUAGACUAUCUGUUC | 23 |
| novel_miR_11 | AAAUCUCUGCAGGCAAAUGUG | 21 |
| novel_miR_340 | AAAAAGUUUGUCUGGGUUUUUCU | 23 |
| novel_miR_848 | CUUGGGCUGGCUCCUCAGAGC | 21 |
| novel_miR_937 | ACUCCCGGUAGGCAACGCGCC | 21 |
| novel_miR_452 | UUUGGCAAUGGUAGAACUCACACU | 24 |
| novel_miR_446 | AAGAACCUGAACGAACCUUUUG | 22 |
| novel_miR_636 | GCGACCCAUACUUGGUUUCAGA | 22 |
| novel_miR_976 | UGUAAAUGUUUCUCUCUGCAGG | 22 |
| novel_miR_1041 | AAAACCUGAAUGAACUUUUUGG | 22 |
| novel_miR_46 | UAGCAGCACAUAAUGGUUUGU | 21 |
| novel_miR_17 | CAGUUACCGCUUCCGCUACCGC | 22 |
| novel_miR_969 | AGAAAAACCUGAAUGAACUUUU | 22 |
| novel_miR_19 | AGCUACAUCUGGCUACUGGGUCUCU | 25 |
| novel_miR_501 | UGAAUGAACUUUUUGGCCAAUU | 22 |
| novel_miR_164 | AGGCAGUGUAGUUAGCUGAUUGC | 23 |
| novel_miR_392 | UAAAGUUCAUUUGGGUUUUUCU | 22 |
| novel_miR_376 | AGGCCAAAGCAAACGACUGUGC | 22 |
| oar-miR-150 | UCUCCCAACCCUUGUACCAGUG | 22 |
| oar-miR-107 | AGCAGCAUUGUACAGGGCUAUC | 22 |
| novel_miR_495 | UUGUGCUUGAUCUAACCAUGU | 21 |
| novel_miR_940 | ACUGGACCUGGAGUCAGAU | 19 |
| novel_miR_562 | UGAUAUGUUUGAUAUAUUAGGUU | 23 |
| novel_miR_672 | AACAUUCAUUGCUGUCGGUGGGU | 23 |
| novel_miR_790 | UAUGUGCUUUGUCUUCUUAGG | 21 |
| novel_miR_86 | ACUGGACACAGAUUUUGAGGG | 21 |
| novel_miR_836 | AUCUGAACAAACUUUUUGGCC | 21 |
| novel_miR_90 | AAACCCGAACGAACUUUUGGGC | 22 |
| novel_miR_497 | AAAAACCUGAACGAACUCUUU | 21 |
| novel_miR_296 | AAAAACCUGAAUGAACUUUCUAG | 23 |
| novel_miR_613 | UAGUGCAAUAUUGCUUAUAGGGU | 23 |
| novel_miR_409 | UCAAAGGCAGGAGCUUAGAUUG | 22 |
| novel_miR_962 | UAAUACUGCCUGGUAAUGAUGAC | 23 |
| novel_miR_692 | AAAGACCCAAAUGAACUUUUU | 21 |
| novel_miR_885 | CUCCUGUCAGCCCUGGGUCUG | 21 |
| novel_miR_697 | AAAACCCCAAAUGAACUUUUUGA | 23 |
| novel_miR_251 | UGAGAACUGAAUUCCAUAGGUU | 22 |
| novel_miR_602 | UUGUGUCAAUAUGCGAUGAUGU | 22 |
| novel_miR_667 | AUGCACCUGGACAAGGAUUCA | 21 |
| novel_miR_581 | AAAACCUGAAUGAACUUUUUGG | 22 |
| novel_miR_92 | UGAUGUGCCUCUCUCCACCCCCAGG | 25 |
| novel_miR_941 | UUUUGUUCCAGAUUUCCAGCUG | 22 |
| novel_miR_1066 | AGAUCAGAAGGUGAUUGUGGCU | 22 |
| novel_miR_412 | CAGUGCAAUGUUAAAAGGGC | 20 |
| novel_miR_78 | CCUCCUCGUAGAGUUCAGACG | 21 |
| oar-miR-377-5p | AGAGGUUGCCUUUGGUGAAUUC | 22 |
| novel_miR_54 | UCUGGUGGGAAGGAAGGGAC | 20 |
| novel_miR_31 | AAAGUUCGUUCAGGCUUUUCUG | 22 |
| novel_miR_399 | AAACCCGAAUGAACUUUUUG | 20 |
| novel_miR_756 | UUGGCCAAAAAGUUCAUUUGA | 21 |
| oar-miR-411b-3p | UAUGUCACAUGGUCCACUAAU | 21 |
| novel_miR_778 | AUGCUGUGCCCAUGUGUCAC | 20 |
| novel_miR_217 | UACUGUGCCACAGCUGGGUAGA | 22 |
| novel_miR_971 | AAGAACCUGAAUGAACUUUUUG | 22 |
| novel_miR_1133 | AAUAUUAUACAGUCAACCUCU | 21 |
| oar-miR-181a | AACAUUCAACGCUGUCGGUGAGU | 23 |
| novel_miR_185 | UGGAGUGACUGUCAGAUGCAGCC | 23 |
| novel_miR_619 | AAAAAGUUCGUUUGGGUUUUUC | 22 |
| novel_miR_63 | AAAACCAGAACGAACUUUUUG | 21 |
| novel_miR_816 | UUUGGGGGUUGCUGUGGCAGAGG | 23 |
| novel_miR_789 | UUAAUUUUUGCAAGGCUUUUCC | 22 |
| oar-miR-3956-5p | GUACGUGGAUGCUGAAGGUCAGA | 23 |
| oar-miR-412-3p | UGGUCGACCAGUUGGAAAGUAAU | 23 |
| oar-miR-376e-5p | GGUGGAUAUUCCUUCUAUGUUU | 22 |
| novel_miR_766 | UGCGGGAUCUUUAGUUGUGGCG | 22 |
| novel_miR_122 | AAAAUCUGAACAAACUUUUUGG | 22 |
| novel_miR_979 | UAACCUUGUAUAAUCUCUUGCCC | 23 |
| novel_miR_81 | AUGAACUUUUGGGCCAACCCAA | 22 |
| novel_miR_734 | AGUGGGCUGGAAGAUGAGCUGGU | 23 |
| novel_miR_1024 | AAAAUCUGAAUGAACUUUUU | 20 |
| novel_miR_839 | UCAGAUACGACUGAAGUGACU | 21 |
| novel_miR_894 | UUUCCAACUGUCCUGCAAGGA | 21 |
| novel_miR_361 | UGAGCAAGACUGUUUUUCUGU | 21 |
| novel_miR_1014 | UGGAAGACUAGUGAUUUUGUUGUU | 24 |
| novel_miR_1073 | UCUGGAUGUAGUUGUGCUGCAGC | 23 |
| novel_miR_210 | UGUGCAGAAGCCCUGAGUCCAUG | 23 |
| novel_miR_147 | AAAGGCCUGAAUGAACUUUUUG | 22 |
| novel_miR_249 | CUUCUGUCCGUCUUGCUACCA | 21 |
| novel_miR_983 | CAAAGAGUCAGACACUACUGA | 21 |
| novel_miR_488 | AAACCCGAAUGAACUUUUUG | 20 |
| novel_miR_945 | UGGAAAAACCCAAACGAACUCU | 22 |
| novel_miR_830 | CACUGCCCCUGUGCCUGCCUCC | 22 |
| novel_miR_368 | AAACCCGAACGAACUUUUGGGC | 22 |
| novel_miR_1125 | AAAACCUGAAUGAACUUUUUGG | 22 |
| novel_miR_717 | UACUUGUUAGAUGUUGAAUA | 20 |
| novel_miR_180 | UGAACAGUGCCUGCUUCUGAUGC | 23 |
| novel_miR_853 | AAAACCCGAAUGAACUUUUG | 20 |
| novel_miR_104 | AGAAAAACCUGAAUGAACUUUU | 22 |
| novel_miR_710 | UGUGCUGUGUCUUCCUGUCAGA | 22 |
| novel_miR_970 | UGGAGCCGGAGCUGGUUAGC | 20 |
| oar-miR-154a-5p | UAGGUUAUCCGUGUAGCCUUCG | 22 |
| novel_miR_575 | UCUCGAGAUUCUUUACUGUCUGA | 23 |
| novel_miR_684 | GUGGCUCAGCUGGUAAAGAAUCUGC | 25 |
| novel_miR_1068 | CGCGGUCACACUGAGGGCGG | 20 |
| novel_miR_639 | UCCGUCAACCAUCCAGCUGUUU | 22 |
| novel_miR_354 | GAGCUCUCUCUCUGUCCCCAGA | 22 |
| novel_miR_712 | UCAGUGCAUCACAGAACUUUGU | 22 |
| novel_miR_665 | GAAAACCCAAAUGAACUUUUUG | 22 |
| novel_miR_980 | UGGGGGGCGGUGAUGGGACUGC | 22 |
| novel_miR_814 | AAUGGGGCAGGUGGAUGGAGUCC | 23 |
| novel_miR_268 | UCUGGGAGGACAGGGCUCAGGGA | 23 |
| novel_miR_812 | UGGCUGUUGUGGUGUGCAAAAC | 22 |
| novel_miR_685 | AAAAACCCAAGUGAACUUUUUG | 22 |
| oar-miR-30d | UGUAAACAUCCCCGACUGG | 19 |
| novel_miR_957 | UAAGGGGCUGAGACCUACUCUGUU | 24 |
| novel_miR_450 | AAAAGUUCGUUUGGGUUUUUCC | 22 |
| novel_miR_206 | AGUAAGGAAAAGGCUUGUUAGGA | 23 |
| novel_miR_238 | UGCUGCAAGGAUGGCUGC | 18 |
| oar-miR-299-5p | UGGUUUACCGUCCCACAUACAU | 22 |
| oar-miR-665-5p | AGGGGUCUUGGCCUCUGCCCAG | 22 |
| novel_miR_110 | UCACUCAGUCGUGUCUGACUCU | 22 |
| novel_miR_1021 | CAAAAAGUUCGUUUGGGUUUU | 21 |
| novel_miR_363 | UCAUGUGUGAUCUGUCCCCAGG | 22 |
| novel_miR_823 | UACCUGGUUGAUCCUGCC | 18 |
| novel_miR_1052 | AAAAUCCGAACGAACUUUU | 19 |
| novel_miR_1119 | AAAACCCAGACGAACUUUUUG | 21 |
| oar-miR-655-5p | AGAGGUUAUCCGUGUUAUGUUC | 22 |
| oar-miR-655-3p | AUAAUACAUGGUUAACCUCUCU | 22 |
| novel_miR_1124 | UUGGCAUUACUGAGCAUCUAGU | 22 |
| oar-miR-99a | AACCCGUAGAUCCGAUCUUG | 20 |
| novel_miR_771 | UGAGAACUGAAUUCCAUAGGCUG | 23 |
| novel_miR_887 | GGAGAUCAGUCCUGGGUGU | 19 |
| novel_miR_142 | GUGCAUUGUAGUUGCAUUGC | 20 |
| oar-miR-134-3p | UCUGGGCUGCCUCGUCACCAACC | 23 |
| novel_miR_1108 | CUUUCACUGUAGAGGAUGCAAG | 22 |
| novel_miR_1038 | AGUGUGUGUGUGUGAGUGUGUGU | 23 |
| novel_miR_499 | UAUUGCACUUGUCCCGGCCUGU | 22 |
| novel_miR_456 | UACUGAGUCCCAGCUGCUGAGC | 22 |
| novel_miR_121 | AAAAUCUGAAUGAACUUUUUGG | 22 |
| novel_miR_513 | AGAUGCAGCUCAUGGGCUCUAG | 22 |
| novel_miR_1104 | UUCUUCCCACGCGUGUCCGCAGG | 23 |
| oar-miR-543-5p | ACCUGUGGUGCUUAAGGAG | 19 |
| novel_miR_882 | AGUAGUGCCUCUCUGAGUGUAG | 22 |
| oar-miR-3957-5p | CUCGGAGAGUGGAGCUGUGGGUGU | 24 |
| novel_miR_98 | AAAAGAUUUGUUUGGGUUUUUC | 22 |
| novel_miR_899 | UUAGGAACAGUAGCAUUUGGGUG | 23 |
| novel_miR_986 | UUGUCUGCAGAUAGUGGGCUCUGG | 24 |
| novel_miR_555 | AAAAACCUGAACAAACUUUUGG | 22 |
| novel_miR_1079 | UGACCGAUUUCUCCUGGUGUU | 21 |
| novel_miR_1092 | AACCCGUAGAUCCGAACUUGUG | 22 |
| novel_miR_909 | AGAGACCUAGGGCCACUCUGA | 21 |
| novel_miR_974 | CCUCCCCUCCAUCUCCCGCCCCAGG | 25 |
| novel_miR_3 | UACAGCUCUCUGGACUUCAG | 20 |
| novel_miR_709 | AUCCGUUGUCUGUAAAGUAGA | 21 |
| oar-miR-299-3p | UAUGUGGGACGGUAAACCACAA | 22 |
| novel_miR_965 | UGAAAAGUUCGUUCGGGUUUUU | 22 |
| novel_miR_506 | ACUGCCCUUCUGCCCCUGCCAGG | 23 |
| novel_miR_758 | UCAAACUCGUGUCUGAUUCGUU | 22 |
| novel_miR_1118 | AAAACCGGAAUGAACUUUUUGA | 22 |
| novel_miR_888 | AAUAGCUCAGAAUGUCACUUCUG | 23 |
| novel_miR_699 | UGGUGCUCCCUGGAGCUGAGC | 21 |
| novel_miR_1005 | AAAAACCUGAAUGAACUCUUUG | 22 |
| novel_miR_1026 | ACUUUCCCGGGACUUGGAGCGC | 22 |
| novel_miR_384 | AAAAACCCGAAUGAACUUUUGG | 22 |
| novel_miR_800 | AAAACCUGAAUGAACUUUUUGG | 22 |
| novel_miR_315 | UCACGGCCUCUCUGCCCCCAGG | 22 |
| novel_miR_688 | UACUUGUUAGAUGUUGAAUA | 20 |
| novel_miR_977 | UCACUUCAGUCGUGUCCGACUCU | 23 |
| novel_miR_25 | UAUGUGCCUUUGGACUACAUCGU | 23 |
| novel_miR_209 | UCGCUCAUUCAUGUCUGACU | 20 |
| novel_miR_199 | AAAAGGUUCGUUCGGGUUUUUC | 22 |
| novel_miR_875 | AAAGCCUGAAUGAACUCUUUGGC | 23 |
| novel_miR_311 | CUGUGGGGUCCCUGGGUCCUGAC | 23 |
| novel_miR_1126 | GUGGCUCAGCUGGUAAAGAAUCUGC | 25 |
| novel_miR_312 | AAAACCCGAACGAGCUCUUUGG | 22 |
| novel_miR_620 | UUUGGACUUGAUAUUGCAUGUUU | 23 |
| oar-miR-30b | UGUAAACAUCCUACACUCAGC | 21 |
| novel_miR_298 | UGUGGGGACCUCUCAUGUCUGU | 22 |
| novel_miR_383 | AAAAACCCGAAUGAACUUUUGG | 22 |
| novel_miR_205 | UACGGGAUCUUUAGUUGUGGCU | 22 |
| novel_miR_1055 | UUCAGAACGGACUGCUGAGUGC | 22 |
| novel_miR_364 | GCAAACGAACUUUUUGGCCAAC | 22 |
| novel_miR_1015 | UAUUGCACUCGUCCCGGCCUCC | 22 |
| novel_miR_910 | CAUGUGGGCUAUAGUUCUCUAA | 22 |
| novel_miR_600 | GUGGUGCCAGCUCUGAGCAGA | 21 |
| novel_miR_75 | CACUGAGGGCAUGGUGUCUGUCA | 23 |
| novel_miR_864 | UUGAACUGUUAAGAACCACUGG | 22 |
| novel_miR_352 | AAACUGGAGUGAACUUUUUGGU | 22 |
| novel_miR_55 | CAAAGUGCUGUUCGUGCAGGUAG | 23 |
| oar-miR-10a | UACCCUGUAGAUCCGAAUUUG | 21 |
| novel_miR_599 | AAAACCUGAAUGAACUUUAUGGU | 23 |
| novel_miR_338 | AAGAACCUGAAUGAACUUUUUG | 22 |
| novel_miR_408 | GUAUGUGGGACGGUAAACCAU | 21 |
| novel_miR_985 | UUUGGCACUAGCACAUUUUUGCU | 23 |
| novel_miR_625 | AAUCACUAGUUCCACUGCCAUC | 22 |
| novel_miR_987 | UAGCCUGGAGAAUCCCGUG | 19 |
| novel_miR_191 | UGAGGGGCAGAGAGCGAGACUUU | 23 |
| novel_miR_162 | GUGGGGCCCGGGGGGGUUGC | 20 |
| oar-miR-539-5p | AGAAAUUAUCCUUGGUGUGUUC | 22 |
| oar-miR-376c-5p | GUGGAUAUUCCGUCUAUGUUU | 21 |
| novel_miR_284 | AAGAGUUGGACACGACUGAGCA | 22 |
| oar-miR-487a-3p | AUCAUACAGGGACAUCCAGUUU | 22 |
| novel_miR_18 | UCCAGACGGUGCUGGCUCUCUGA | 23 |
| novel_miR_43 | UUCUCCAGGCAAGAACACUGG | 21 |
| novel_miR_750 | AAAGGUUCAUUCAGGUUUUUUU | 22 |
| novel_miR_502 | AAAUCUGAACAAGCUUUUUGGC | 22 |
| oar-miR-410-5p | CGCCACUUUGGGUACUUGAGGAG | 23 |
| novel_miR_304 | AAGGGACCCGAACGAACUUUUU | 22 |
| novel_miR_48 | AAAAACUCAGAUGAACUUUUUG | 22 |
| novel_miR_219 | AACCGGACGUGGACCGCAGGGUUG | 24 |
| novel_miR_281 | UCCCUGUCUUCAAUCCUGUAGU | 22 |
| novel_miR_59 | AAAAACUCAGAUGAACUUUUUG | 22 |
| novel_miR_130 | AAAAUCCAAACGAACUUUUUGA | 22 |
| novel_miR_1067 | AAGGGCCGCUGGCAGCAGCUGUGG | 24 |
| novel_miR_876 | GAUGCCGCGCUGGACCUUUGCCC | 23 |
| novel_miR_328 | AAACCCUGAAUGAACUUUUUGG | 22 |
| novel_miR_722 | GGGGCUGGCCAUAGCUCAGCGGU | 23 |
| novel_miR_444 | AAUGGAUUACUUCUGAUUGCUG | 22 |
| novel_miR_1114 | UCUCACACAGAAAUCGCACCCAUC | 24 |
| novel_miR_892 | UAUUGCACAUUACUAAGUUGCA | 22 |
| novel_miR_1046 | GAAAAACCUGAAUGAACUUUU | 21 |
| novel_miR_531 | UGACUCCGGGCUCCUCCCUCAG | 22 |
| novel_miR_615 | UCCUGUGAUGUGUCUCUGAAC | 21 |
| novel_miR_291 | UUGCUGCGGGUGUCAGGAAGACA | 23 |
| oar-miR-665-3p | ACCAGUAGGCCGAGGCCCCUCA | 22 |
| oar-miR-127 | AUCGGAUCCGUCUGAGCUUGGCU | 23 |
| novel_miR_884 | UAACACUGUCUGGUAAAGAUGGC | 23 |
| oar-miR-487a-5p | GUGGCUAUCCCUGCUGUGUUCG | 22 |
| novel_miR_577 | UAGGUAGUUUCCUGUUGUUGGG | 22 |
| novel_miR_781 | GGAGGACUGAGGUUUGGUCUGG | 22 |
| novel_miR_256 | UUAUCAGAAUCUCCAGGGGUAC | 22 |
| novel_miR_680 | CUUUUUGCGGUCUGGGCUUGC | 21 |
| novel_miR_900 | CCAAUAUUGGCUGUGCUGCUCC | 22 |
| novel_miR_141 | AAAAUCUGAACAAACUUUUU | 20 |
| oar-miR-136 | ACUCCAUUUGUUUUGAUGAUGGA | 23 |
| novel_miR_549 | UGAGGUAGGAGGUUGUAUAGUUG | 23 |
| novel_miR_148 | AAAGCCCAAACGAACUUUUUGG | 22 |
| novel_miR_1078 | UGACUGUCUGGUGUCUUCCCAG | 22 |
| novel_miR_39 | GCGGGCUUCCCUGGUGGCUCAGC | 23 |
| novel_miR_1098 | GAAAACCUAAAUGAACCUUUUG | 22 |
| novel_miR_772 | AAAACCUGAAUGAACUUAUUGG | 22 |
| novel_miR_760 | CUGGCCCUCUCUGCCCUUCCGU | 22 |
| novel_miR_419 | AAAAAGUUUGUUCGGGUUUU | 20 |
| novel_miR_538 | GGGGCUUCCCUGGUGGCUCAGA | 22 |
| novel_miR_961 | AUGCUGACAUAUUUACUAGAGG | 22 |
| novel_miR_484 | UUCACAGUGGCUAAGUUCUGCA | 22 |
| novel_miR_921 | GAGGAAGUCAGUCUGCAGAGA | 21 |
| novel_miR_984 | AAAAUCCGAACGAACUUUUUGG | 22 |
| novel_miR_1088 | AAAACCCAGACGAACUUUUUG | 21 |
| novel_miR_757 | CAAAGAAUUCUCCUUUUGGGCU | 22 |
| novel_miR_663 | UUUUCCUGAUCUGUUUUCUAAU | 22 |
| novel_miR_1059 | UGUGGGGGGGGGGAGGCGGG | 20 |
| novel_miR_893 | GAAAAUCCGAAUGAACUUUUUUG | 23 |
| oar-miR-29b | UAGCACCAUUUGAAAUCAGUGU | 22 |
| novel_miR_1043 | AAAACCUGAAUGAACUUAUUGG | 22 |
| novel_miR_57 | AAAAUCUGAACAAACUUUUU | 20 |
| novel_miR_124 | AAAACCUGAAUGAACUUUUUGG | 22 |
| novel_miR_915 | UAGCAGCGGGAACAGUACUGCAG | 23 |
| novel_miR_216 | UGUGGAGAGGACGGAAACCGCGC | 23 |
| novel_miR_158 | ACCGGUUCUGAUCCCCCUG | 19 |
| novel_miR_225 | AAAAGUUCGUUCGGGUUUUU | 20 |
| novel_miR_860 | AAAGUUCGUUCAGGCUUUUCUG | 22 |
| novel_miR_449 | UGACUGCUCUCUCUCCUUCCCAGU | 24 |
| novel_miR_714 | UCGGUCCCCUGCCCCCUCUGC | 21 |
| novel_miR_749 | UGAGUGUGUGUGUGUGAGUGA | 21 |
| novel_miR_262 | AAAAAUCUGAAUGAACUUU | 19 |
| novel_miR_690 | AAAAACCUGAACAAACUUUUUG | 22 |
| novel_miR_289 | AGGCCAAAGCAAACGACUGUGC | 22 |
| oar-miR-362 | AAUCCUUGGAACCUAGGUGUGAGU | 24 |
| novel_miR_1004 | UAGGUAGUUUUAUGUUGUUGGG | 22 |
| novel_miR_949 | UAAGGCACGCGGUGAAUGCC | 20 |
| novel_miR_451 | AAAAUCCGAGCGAACUUUUUGGU | 23 |
| novel_miR_1083 | AGGGUCUCAGAAGCACUGGUUG | 22 |
| novel_miR_455 | CGCAGAGGCGGGAGGUAUGACCU | 23 |
| novel_miR_515 | AAUUGCACGGUAUCCAUCUGCG | 22 |
| novel_miR_638 | AGAAGAUCUGUGGUGGUUCCC | 21 |
| novel_miR_905 | AAAAUCUGAACAAACUUUCUGG | 22 |
| oar-miR-433-5p | UACGGUGAGCCUGUCAUUGUUCA | 23 |
| novel_miR_643 | UGGCCCCGGCGUCCCUGCUGCA | 22 |
| oar-miR-19b | UGUGCAAAUCCAUGCAAAACUGA | 23 |
| novel_miR_556 | AAAAACUCAAAUGAACUUUUG | 21 |
| novel_miR_221 | GAAAACCUGAACAAACUUUGUG | 22 |
| novel_miR_810 | CUCCCUCGGAACCCGGCUGGGACCU | 25 |
| novel_miR_1074 | UUAGAGAUAAGAGUGUAAGAAGG | 23 |
| novel_miR_1095 | UGUGUAUUUGACAAGCUGAGUUG | 23 |
| novel_miR_427 | AUAAACCUGAACGAACUUUUUGG | 23 |
| novel_miR_584 | AAAAUCUGAAUGAACUUUUGGC | 22 |
| novel_miR_173 | AGCCCCUGCCUGGGCCCGGAGCCU | 24 |
| novel_miR_527 | AUCAACAAACAUUUAUUGUGUGC | 23 |
| novel_miR_7 | AGGCAAGAUGCUGGCAUAGCUGU | 23 |
| novel_miR_71 | AGGUUCUGUGAUACACUCCGACU | 23 |
| novel_miR_42 | AAUCCCAUGGACAGAGAAGCC | 21 |
| oar-miR-3955-5p | UUUGAUGGCUGAUCCUCUCACU | 22 |
| novel_miR_767 | AAAAACUCAAAUGAACUUUUG | 21 |
| novel_miR_421 | AAACCCGAAUGAACUUUUUG | 20 |
| novel_miR_993 | UCGUACCGUGAGUAAUAAUGCG | 22 |
| oar-miR-381-3p | AUAUACAAGGGCAAGCUCUCU | 21 |
| novel_miR_505 | UGAAAAGACUCAGACAAUUGCU | 22 |
| novel_miR_259 | UUUUGUGUUUCUCGAACUACCC | 22 |
| novel_miR_930 | GAAACCUGAAUGAACUUUUUGA | 22 |
| novel_miR_563 | GAAAACCCAAAUGAACUUUUUG | 22 |
| novel_miR_1019 | AAAGCUGAACGAACUUUUUGGC | 22 |
| novel_miR_170 | UGAGGUAUUUUGUGCUGUU | 19 |
| novel_miR_782 | UUGGACACUUCAGUACUGCUACA | 23 |
| novel_miR_849 | AACUGGCCCACAAAGUCCCGCU | 22 |
| novel_miR_20 | AAAACCUGAAUGAACUUUUU | 20 |
| novel_miR_14 | AAAAAGUUUGUUUGGGUUUUUCU | 23 |
| novel_miR_743 | GAAACCUGAAUGAACUUUUUGA | 22 |
| oar-miR-1197-5p | CCCUUCCUGGUAUUUGAAGACG | 22 |
| oar-miR-539-3p | AAUCAUACAAGGACAAUUUCUUU | 23 |
| novel_miR_996 | CUUGAGGGUGACCAGGGAGACA | 22 |
| novel_miR_88 | AAAAUCUGAAUGAACUUUUGGC | 22 |
| novel_miR_975 | GAAAAGUUCAUUCGGGUUUUUCU | 23 |
| novel_miR_150 | UGGAUGCUUGUCUUUUGGGC | 20 |
| novel_miR_878 | UGGAAGACUAGUGAUUUUGUUGUU | 24 |
| novel_miR_797 | AGGAAAACCCAAAUGAACUUU | 21 |
| oar-miR-431 | UGUCUUGCAGGCCGUCAUGCAGG | 23 |
| novel_miR_719 | CGGCUCAGUGGGACCCCCUCACU | 23 |
| novel_miR_411 | CCUGUCCUGGCCUGUGGA | 18 |
| novel_miR_510 | AAAACCUGAGUGAACUUUUUGG | 22 |
| novel_miR_325 | AUGAACUUUUGGGCCAACCCAA | 22 |
| oar-miR-10b | ACCCUGUAGAACCGAAUUUGUG | 22 |
| novel_miR_118 | AAAACCUGAAUGAACUUAUUGG | 22 |
| novel_miR_968 | AAAACCCGCAUGAACUUUUUGG | 22 |
| novel_miR_807 | AAAACCUGAAUGAACUUUUUGG | 22 |
| novel_miR_647 | CCUCCGCCCUUGCCCCUCAGG | 21 |
| novel_miR_266 | AAUAAGUUCGUUUGGGUUUUU | 21 |
| novel_miR_959 | UAUAUAUAUAUAUGUACGUAU | 21 |
| novel_miR_211 | CCGGCGGUCUUCGGUAUACG | 20 |
| novel_miR_713 | AAAAUCUGAACAAACUUUUUGG | 22 |
| novel_miR_195 | GAAACCUGAGUGAACUUUUUGG | 22 |
| novel_miR_37 | CAUCCUGGAUCCUCUUAACC | 20 |
| novel_miR_952 | UACAGUGACCAGGUGACGACGG | 22 |
| oar-miR-541-5p | AAAGGAUUCUGCUGUCGGUCCCACU | 25 |
| novel_miR_598 | AAAACCUGAGUGAACUUUUUGG | 22 |
| oar-miR-370-5p | CAGGUCACGUCUCUGCAGUUAC | 22 |
| novel_miR_903 | AAACUUGAAUGAAUGUUUUGGCC | 23 |
| novel_miR_752 | AAAAAGUUCGUUUGGGUUUUUC | 22 |
| novel_miR_504 | AAAGGUUCAUUUGUGUUUUUCU | 22 |
| novel_miR_293 | AAUGUUCUGUAUCUUGUUGUAU | 22 |
| oar-miR-125b | UCCCUGAGACCCUAACUUGUG | 21 |
| novel_miR_351 | AAAAACCCAAAUGAACUUUUUG | 22 |
| novel_miR_171 | AAAAGUUCGUUCGGGUUUUUC | 21 |
| novel_miR_574 | UGGCAGUGUCUUAGCUGGUUGUU | 23 |
| novel_miR_539 | UGGAAGGCCUGGCUUUGCAGCGU | 23 |
| novel_miR_833 | CACUGGAGUUUUGUUUCAACAUU | 23 |
| oar-miR-1193-5p | GGGAUGGUAGACCGGUGACGUGC | 23 |
| oar-miR-3955-3p | UGGGAUUUUAGUCCAUCAUAG | 21 |
| novel_miR_914 | AGACCCUGGUCUGCACUCUGUC | 22 |
| novel_miR_764 | UGCCUCUCCGCCACCUCCACC | 21 |
| novel_miR_286 | UGUACUCUGGGACUCGGGUGUCA | 23 |
| oar-miR-379-3p | UAUGUAACAUGGUCCACUAACU | 22 |
| oar-miR-3959-3p | UGUAUGUCAACUGAUCCACAGU | 22 |
| novel_miR_889 | UUGGGGAAGCGCAGGAAACAG | 21 |
| novel_miR_84 | AUCCUGGACUUGCGGACAGAGAGC | 24 |
| novel_miR_396 | AAAACCUGAAUGAACUUUUUGG | 22 |
| novel_miR_430 | GCAAAGCACACGGCCUGCAGAGA | 23 |
| novel_miR_537 | CUGGGGUCUCUGGCGUCUGAGG | 22 |
| novel_miR_628 | AAAACCUGAACAAACUUUUGGG | 22 |
| novel_miR_953 | AAACCCGAAUGAACUUUUUG | 20 |
| novel_miR_1028 | AUUGGGCAUGAUUGAAGCGACU | 22 |
| novel_miR_460 | UUGGACAGGACUGAGUGACUAA | 22 |
| oar-miR-23a | AUCACAUUGCCAGGGAUUUCCA | 22 |
| novel_miR_803 | AAAACCCGAAGGAACAUUUUGG | 22 |
| novel_miR_587 | AAACCUGAAUAAACUUUUUGA | 21 |
| novel_miR_407 | CUGGAGCUGAGAGGCAGCUGUGC | 23 |
| novel_miR_82 | AGAAUACCCAAACCACACACCA | 22 |
| novel_miR_664 | AAAACCUGAAUGAACUUUUUGG | 22 |
| novel_miR_1110 | AAAGCCCAAACGAACUUUUUGG | 22 |
| novel_miR_487 | UGCCAAGCCCACGUUCAAAGG | 21 |
| novel_miR_1069 | AAACAAGAUCACGCCUCUCAGA | 22 |
| novel_miR_592 | GACUUACUGUCUCUGAGCUUUA | 22 |
| novel_miR_831 | UCACUCAGUCGUGCCCGAAUUACU | 24 |
| novel_miR_341 | UUCAAGUAAUCCAGGAUAGGCU | 22 |
| novel_miR_431 | UCACUGGGCAUCCUCUGCUUUA | 22 |
| novel_miR_290 | AAAGCCCAAACGAACUUUUUGG | 22 |
| oar-miR-494-3p | UGAAACAUACACGGGAAACCUCU | 23 |
| novel_miR_847 | CAGACGUGACUGCGCCCUGAGAAG | 24 |
| novel_miR_240 | UAGUGUGCUAGAGUCCUCGAAGA | 23 |
| novel_miR_23 | AAAAACUGGCAGCUUCAUGUAAU | 23 |
| novel_miR_255 | AAAGUCUGAAUGAACUUUUUGGU | 23 |
| novel_miR_250 | AGCAGAGCGAGCAGGACAGUGC | 22 |
| novel_miR_213 | AAAAAUCUGCAUGAACUUUGUGG | 23 |
| novel_miR_1129 | UUCUCUGUUCAUGUGGACGUGU | 22 |
| oar-let-7a | UGAGGUAGUAGGUUGUAUAGUU | 22 |
| oar-miR-433-3p | AUCAUGAUGGGCUCCUCGGUGU | 22 |
| novel_miR_1132 | AUUCUCCGGGCAGGAAUGCUGG | 22 |
| novel_miR_590 | AAAAUCCGAAUGAACUUUUUGGU | 23 |
| novel_miR_1113 | UACUGUGCCACGGAUGGGUAGC | 22 |
| novel_miR_140 | UGAAAAGUUUGUUCGGGUUUUU | 22 |
| novel_miR_657 | UGCCUCUCCGCCACCUCCACCUC | 23 |
| novel_miR_168 | UGGAAAAACCCAAACGAACUUU | 22 |
| novel_miR_4 | UGAAUGAACUUUUUGGCCAAC | 21 |
| novel_miR_774 | UUGCUCCCUCCACACUUCCAGA | 22 |
| novel_miR_1056 | ACUGGACUUGGAGUCAGA | 18 |
| novel_miR_434 | CUGAGGAAAGUGUGGGCCCACA | 22 |
| novel_miR_489 | CUUGUUCUCCAACCUGGCUCUUU | 23 |
| novel_miR_776 | UCUGGUGCUUAGACUCUGUGCU | 22 |
| novel_miR_964 | AUGAACUUUUGGGCCAACCCAA | 22 |
| oar-miR-1185-5p | AGAGGAUACCCUUUGUAUGUUC | 22 |
| novel_miR_618 | UGCGCCCCCGCGCCCCCCGCGC | 22 |
| novel_miR_194 | UUCUUUUCGGGUAGUUUAUGA | 21 |
| novel_miR_362 | AGAUCUGUCCUGAAACCAGCAU | 22 |
| novel_miR_1027 | AAAAUCUGAGUGAACUUUUUGG | 22 |
| oar-miR-493-3p | UGAAGGUCUACUGUGUGCCAGG | 22 |
| novel_miR_270 | ACUCAAACUGUGGGGGCACUUC | 22 |
| novel_miR_348 | AGCAGCAUUGUACAGGGCUAUGAAA | 25 |
| novel_miR_301 | GAAAACCUGAACAAACUUUUGG | 22 |
| novel_miR_595 | AAUGGUGCUUUUUUGUGAAGA | 21 |
| oar-miR-376c-3p | AACAUAGAGGAAAUUCCACGU | 21 |
| novel_miR_1018 | UGACACAACUGAGCGACUGAGC | 22 |
| novel_miR_137 | UUCUGAGACAGGAGGGCAGGUGG | 23 |
| novel_miR_593 | AACUGUUUGCAGAGGAAACUGA | 22 |
| novel_miR_1099 | AGCUGGUGUUGUGAAUCAGGCCGU | 24 |
| novel_miR_1025 | GAUGAGGCUCAGCGAGCCCUGGG | 23 |
| novel_miR_522 | UUAGGGCCCUGGCUCCAUCUCC | 22 |
| novel_miR_133 | AAAAAGUUGGUUCAGGUUUUUC | 22 |
| novel_miR_214 | GCGGUCACACUGAGGGCGGGGC | 22 |
| novel_miR_49 | CAACCUGGAGGACUCCAUGCUG | 22 |
| novel_miR_674 | AAAAACCCAAAUGAACUUUUUG | 22 |
| novel_miR_631 | AAAAACCUGAAUGAACUUUUGG | 22 |
| novel_miR_733 | UGACAGCCCUCACCCUUCACAGA | 23 |
| novel_miR_649 | CCCCUGGCUGAGCGGUCUCUCC | 22 |
| novel_miR_1130 | UACUGCAUCAGGAACUGAUUGGA | 23 |
| novel_miR_388 | AAACCCGAACGAACUUUUGGGC | 22 |
| novel_miR_64 | AAAAACCCAGAUGAACUUUUUU | 22 |
| novel_miR_146 | AUGAACUUUUGGGCCAACCCAUU | 23 |
| novel_miR_491 | CACAGAGCUCUUCCUGCUGAAGC | 23 |
| novel_miR_481 | AGAAAAACCUGAAUGAACUUUU | 22 |
| novel_miR_50 | UACCGGGCCAGCUGGAAGGA | 20 |
| novel_miR_264 | AGUGGGGAACCCUUCCAUGAGGA | 23 |
| novel_miR_705 | AAAAUCUGAAUGAACUUUUU | 20 |
| novel_miR_907 | UCAAGCACCACAGCUCCUGAGC | 22 |
| novel_miR_779 | UAUGGCUUUUUAUUCCUAUGUG | 22 |
| novel_miR_144 | CAAAUGAACUUUCUGGCCAACC | 22 |
| novel_miR_1048 | CACUGUAUAGUUACCCUCUUU | 21 |
| novel_miR_901 | CCCAGGGAUGUAGCUCCUAGUGC | 23 |
| novel_miR_95 | UUCUCAUUGGCCUCACGUCCUGUC | 24 |
| novel_miR_287 | AAAAAUCUGAAUGAACUUUUUG | 22 |
| novel_miR_303 | AAAAAUCUGAAUGAACUUUUU | 21 |
| oar-miR-541-3p | UGGUGGGCACAGAAUCCGGCCUCU | 24 |
| novel_miR_314 | AAACCGGCAUGAACUUUUUGGC | 22 |
| novel_miR_1091 | ACGAAUCAGACACGAGUUUG | 20 |
| oar-miR-200c | UAAUACUGCCGGGUAAUGAUGG | 22 |
| novel_miR_357 | CCCUGAACUAGGGGUCUGGAGG | 22 |
| novel_miR_459 | ACUGUUGGGCGUGUCUGACUGU | 22 |
| oar-miR-134-5p | UGUGACUGGUUGACCAGAGGG | 21 |
| novel_miR_475 | AAAAUGUUCGCUUGGGCUUUUUC | 23 |
| novel_miR_655 | UAUGGCACUGGUAGAAUUCACU | 22 |
| novel_miR_656 | CUGCCCUGGCCCGAGGGACCGAC | 23 |
| novel_miR_253 | CCCGGUACUGAGCUGACCCGAG | 22 |
| novel_miR_788 | UGGCUCAGUUCAGCAGGAAC | 20 |
| novel_miR_548 | CAUGGGCUGAGUUGCUCCACAGC | 23 |
| novel_miR_835 | UAUUCCCAGCUUGCAUUCCACU | 22 |
| novel_miR_829 | AAAAGUUCGUUUGGGUUUUUC | 21 |
| novel_miR_547 | UCUUUGGUUAUCUAGCUGUAUGA | 23 |
| novel_miR_346 | AUGAACUUUUGGGCCAACCCAAU | 23 |
| novel_miR_873 | GAUGAGGCUCAGCGAGCCCUGGU | 23 |
| novel_miR_350 | CUGCGUUCGCGCUUUCCCCUG | 21 |
| novel_miR_573 | GAGGAAGUCAGUCUGCAGAGA | 21 |
| novel_miR_805 | AAAAACCAGAAUGAACUUUUC | 21 |
| novel_miR_870 | CCCAGGUUCUAGUGGCCUCCAGC | 23 |
| novel_miR_588 | CCGGACCGAGUCCCCUUCCCCUC | 23 |
| novel_miR_551 | AAAACCUGAAUGAACUUUUUGG | 22 |
| novel_miR_552 | AAUGGCGCCACUAGGGUUGUGC | 22 |
| novel_miR_854 | AAAAACCUGAACGAACUUUUGG | 22 |
| novel_miR_445 | AAAACUUGAAUGAACUUUCUGG | 22 |
| novel_miR_508 | AAAAAUCUGAAUGAACUUU | 19 |
| novel_miR_1081 | AAAAACUCAGAUGAACUUUUUG | 22 |
| oar-miR-758-3p | UUUGUGACCUGGUCCACUAA | 20 |
| novel_miR_661 | CCGGGGAAAGCAGGAGUGAGG | 21 |
| novel_miR_359 | UCAUGGACAAGCUGUGUGGCA | 21 |
| novel_miR_731 | UACUGAGUCCCAGCUGCUGAGC | 22 |
| novel_miR_629 | CAUCCCUUGCAUGGUGGAGGG | 21 |
| novel_miR_406 | AAACCUGAAUAAACUUUUUGA | 21 |
| novel_miR_229 | CACGCUCAUGCACACACCCACA | 22 |
| novel_miR_542 | AAAAACCUGAACGAACUUUUUG | 22 |
| novel_miR_543 | UCAGUUGUGUCCGACUCUGCAA | 22 |
| novel_miR_448 | AAAAACUCAAAUGAACUUUUUG | 22 |
| oar-miR-544-3p | AUUCUGCAUUUUUAGCAAGUU | 21 |
| novel_miR_706 | AGAAACCUGAACGAACUUUUGG | 22 |
| novel_miR_544 | UUGAUGUAUGUAUUCUUGCAGG | 22 |
| novel_miR_323 | AAAACCCGCAUGAACUUUUUGG | 22 |
| novel_miR_869 | AAAAACUCAGAUGAACUUUUUG | 22 |
| novel_miR_617 | AAUAGCUCAGAAUGUCACUUCUG | 23 |
| novel_miR_381 | AAAACCCAAACGAACUUUUUGA | 22 |
| novel_miR_689 | CCCUGUGACAGUGAGCGGC | 19 |
| oar-let-7i | UGAGGUAGUAGUUUGUGCUGUU | 22 |
| novel_miR_310 | AAAACCUGAAUGAACUUUUUGG | 22 |
| novel_miR_154 | UUGUUGUCAGGAGUCACUGCUC | 22 |
| novel_miR_762 | UGAAAAUGACGUGACGGACUUCU | 23 |
| novel_miR_125 | UCCUCACUGUCUCUGCUCUGCAG | 23 |
| oar-miR-329b-3p | AACACACCUGGUUAACCUCUUU | 22 |
| novel_miR_798 | CUGGGAGGAGGCUGUUUACUCU | 22 |
| novel_miR_72 | UCGGAGUGUAUCACAGAACCUGG | 23 |
| novel_miR_1012 | AAAACCUGAACGAACUUUUUGG | 22 |
| novel_miR_540 | AUAAGUUCGUUUGGGUUUUUCC | 22 |
| novel_miR_724 | CAAAGAGUCAGACACUACUGA | 21 |
| novel_miR_958 | AAGGGCGGGACGCCAGAAAUUCU | 23 |
| novel_miR_509 | AAAAGUUCGUUUGGGUUUUUCC | 22 |
| oar-miR-3958-3p | AGAUAUUGCACGGUUGAUCUCU | 22 |
| novel_miR_1089 | GUGGACUUCCCUGGUAGC | 18 |
| novel_miR_308 | AUGAACUUUUGGGCCAACCCAAU | 23 |
| novel_miR_1063 | AAAAUCUGAACAAACUUUUG | 20 |
| novel_miR_856 | CUAGCGGGCGAACCUACUGUGCG | 23 |
| novel_miR_201 | GUGUAGAGGCAGAGAGUUGUAG | 22 |
| novel_miR_1051 | AAAAACCUGAAUGAUGCUUUUG | 22 |
| novel_miR_226 | UGUGGUCUCUCCAGCAAGGUGG | 22 |
| novel_miR_467 | UCAGCACCAUCCUUCCCUGGC | 21 |
| novel_miR_777 | UAUAUAUAUAUAUGUACGUAUG | 22 |
| oar-miR-323b | CCCAAUACACGGUCGAUCUCU | 21 |
| novel_miR_877 | CUCACACACACCCUCCCCCUGCAGU | 25 |
| novel_miR_161 | UCCCUGAGACCCUUUAACCUGUG | 23 |
| novel_miR_811 | AAAAAGUUCGUUUGGGUUUUU | 21 |
| novel_miR_33 | GUGAAAUGUUUAGGACCACUAG | 22 |
| novel_miR_193 | GAAAAACCUGAAUGAACUUUU | 21 |
| novel_miR_711 | UCUUUGGUUAUCUAGCUGUAUGA | 23 |
| novel_miR_163 | UCAUGUGUGAUCUGUCCCCAGG | 22 |
| oar-miR-382-5p | GAAGUUGUUCGUGGUGGAUUCG | 22 |
| novel_miR_591 | AUGCCUUCCCCAGCCUCCGAGC | 22 |
| novel_miR_466 | UGACAGUGGCUAAGUUCU | 18 |
| novel_miR_15 | AAAAGUUCGUUCGGGUUUUUC | 21 |
| oar-miR-23b | AUCACAUUGCCAGGGAUU | 18 |
| oar-miR-376a-3p | AUCAUAGAGGAAAAUCCACGU | 21 |
| novel_miR_94 | UGAAAAUGACGUGACGGACUUCU | 23 |
| oar-let-7d | AGAGGUAGUAGGUUGCAUAG | 20 |
| novel_miR_243 | AAACUUGAGCAAACUUUUUGGC | 22 |
| novel_miR_285 | CCAGGGCCUCUGCCGUCACGCG | 22 |
| novel_miR_753 | UCUGACCACCUGUCUCUCCAUAC | 23 |
| novel_miR_218 | UUGGCCGUGGUCCGGGAGCGG | 21 |
| novel_miR_224 | AGAGUGACCUGUGUGGCUGCCAC | 23 |
| oar-miR-496-5p | GAGUCGGGUACUCGAAUGGA | 20 |
| novel_miR_234 | UUCAUUCGGCUGUCCAGAUGUA | 22 |
| novel_miR_176 | AAAACCUGAAUGAACUUUUUGG | 22 |
| novel_miR_321 | AAAACCUGAAUGAACUUGUUGGU | 23 |
| oar-let-7f | UGAGGUAGUAGAUUGUAUAGU | 21 |
| novel_miR_837 | CCUCAGCCACACCCCUCACACA | 22 |
| oar-miR-26b | UUCAAGUAAUUCAGGAUAGGU | 21 |
| novel_miR_254 | UAAUGCCCCUAAAAAUCCUUAU | 22 |
| novel_miR_189 | CGGGGCUCGGGGCACCGAAGAC | 22 |
| novel_miR_695 | AAAACCCAAACGAACUUUUUGGA | 23 |
| novel_miR_1039 | UUUCCUCUCUGCCCCAUAGGGU | 22 |
| novel_miR_822 | AUGAAGGGUCUGAAGAUGAAG | 21 |
| novel_miR_1072 | AGGGACCUAGACUCCACGCCC | 21 |
| novel_miR_116 | AAAACUCAGAUGAACUUUUUGG | 22 |
| novel_miR_524 | AUCACAUUGCCAGGGAUUACCACG | 24 |
| novel_miR_1011 | AAAACCCGCAUGAACUUUUUGG | 22 |
| novel_miR_793 | UUAAAGUACUGCUGAACUCUUC | 22 |
| novel_miR_1031 | AAAACCUGAAUGAACUUUUUGG | 22 |
| oar-miR-380-5p | AUGGUUGACCACAGAACAUGCG | 22 |
| novel_miR_550 | UACUGUGCCACAGCUGGGUAGA | 22 |
| novel_miR_1071 | AAAACCUGAACUAACUUUUUGA | 22 |
| oar-miR-494-5p | AGGUUAUCCGUGUUGUCUUCUCU | 23 |
| novel_miR_624 | UCAGGCGUUGGCAUUUCAGGUU | 22 |
| novel_miR_232 | GACUGUUCGGAGGAGAAAGAA | 21 |
| novel_miR_426 | AAAACUUGAAUGAACUUUCUGG | 22 |
| oar-miR-493-5p | UUGUACAUGGUAGGCUUUCAUU | 22 |
| novel_miR_635 | GUUGGCCUGAAAGUUUGAGUU | 21 |
| novel_miR_371 | AAAACCAGAACGAACUUUUUG | 21 |
| novel_miR_101 | AAAAACCUGAACAAACUUUUUG | 22 |
| novel_miR_751 | AAAACCUGAGUGAACUUUUUGG | 22 |
| novel_miR_228 | AGAAACCGAACAAACUUUUUGGC | 23 |
| novel_miR_99 | AAAAACCUGAACGAACUUUUC | 21 |
| novel_miR_66 | UCUCCUGGCCCCUCCUGCCCAGG | 23 |
| oar-miR-370-3p | GCCUGCUGGGGUGGAACCUGGUCU | 24 |
| oar-miR-200b | UAAUACUGCCUGGUAAUGAUG | 21 |
| novel_miR_916 | GAAAACCUGAAGGAACUUUUU | 21 |
| novel_miR_606 | UAAGGUGCAUCUAGUGCAGUUAG | 23 |
| oar-miR-3959-5p | GGUUGAUCAGAGAACAUACAUU | 22 |
| novel_miR_1030 | AAAACCAGAACGAACUUUUUGA | 22 |
| novel_miR_53 | UUGGGCAAAAAGUUCAUUUGGG | 22 |
| novel_miR_400 | AUUGCGACCCCAUGGACUGCAGC | 23 |
| novel_miR_1103 | UUUCCCACCUCUUCUCUUGCAGG | 23 |
| oar-miR-758-5p | UGGUUGACCAGAGAGCACACG | 21 |
| novel_miR_490 | UGCUGCACCGGGGUGUCUGGAC | 22 |
| oar-miR-376b-5p | GGUGGAUAUUCCUUCUAUGUUU | 22 |
| novel_miR_30 | AAAAUCUGAGUGAACUUUUUGG | 22 |
| novel_miR_167 | CUAUGGCUCUUAGAAAGUGGUGG | 23 |
| novel_miR_45 | UUAUCAGAAUCUCCAGGGGUAC | 22 |
| novel_miR_1044 | AAAAACCUGAAUGAACCUUUU | 21 |
| novel_miR_177 | AGAGGUAAAAAAUUGAUUUGACU | 23 |
| novel_miR_390 | AACAUCUGGGCGAGAGUUCUCC | 22 |
| novel_miR_523 | AAAACCUGAACAAACUUUUUGG | 22 |
| novel_miR_93 | UGGAAAAACCCAAAUGAACUUC | 22 |
| novel_miR_324 | UCAGAUACGACUGAAGUGACU | 21 |
| novel_miR_271 | GCAAGGGAAAUUAGCUGACUGA | 22 |
| novel_miR_564 | AGUGUUCAAAAAAGGUAGCUGU | 22 |
| novel_miR_818 | UCCCAGGACUGACUCCGUGCCCUU | 24 |
| novel_miR_344 | ACUCUUUCCCUGUUGCACUACU | 22 |
| novel_miR_871 | AGAAAAGUUCGUUCAGGUUUUU | 22 |
| novel_miR_32 | AAAACCUGAAUGAACUUUUUGG | 22 |
| novel_miR_175 | UGGAAAAACCCAAAUGAACUUC | 22 |
| novel_miR_258 | UACAGUACUGUGAUAACUGAAG | 22 |
| novel_miR_29 | UUUUGACUGGGAUCUACCACUGU | 23 |
| novel_miR_248 | UUCUCCUCUCUGUUCUCUAG | 20 |
| novel_miR_770 | AAAAGAUUUGUUUGGGUUUUUC | 22 |
| novel_miR_736 | UGGGCCUGUGGUACCCCCCCCAUC | 24 |
| novel_miR_1106 | AAAAUCUGAACAAACUUUUUGG | 22 |
| novel_miR_370 | AGAACUUAAAUGAACUUUUUG | 21 |
| novel_miR_454 | AGAUCUGUCCUGAAACCAGCAU | 22 |
| novel_miR_1008 | CAAAAAGUUUGUUUGGGUUUUU | 22 |
| oar-miR-30c | UGUAAACAUCCUACACUCUCA | 21 |
| novel_miR_245 | CUCUCUGGACCGAAGUCUGUCCAG | 24 |
| novel_miR_561 | AGAAAAACCUGAAUGAACUUUU | 22 |
| novel_miR_845 | UAUGUGGGACGGUAAACCGCUU | 22 |
| novel_miR_594 | UGGAAGACUAGUGAUUUUGUUGUU | 24 |
| novel_miR_605 | AAAAUCCGAAUGAACUUUUUGG | 22 |
| oar-miR-380-3p | UAUGUAAUGUGGUCCACGUCU | 21 |
| novel_miR_769 | AAUGACACGAUCACUCCCGUUGAGU | 25 |
| novel_miR_989 | UGGCCAAAAGGUUUGUUCAGAU | 22 |
| novel_miR_715 | AAAAAGUUCGUUUGGGUUUUUC | 22 |
| novel_miR_395 | GUCCAGUUUUCCCAGGAAUCCCU | 23 |
| oar-miR-485-5p | AGAGGCUGGCCGUGAUGAAUUCG | 23 |
| novel_miR_707 | AAAACCUGAAUGAACUUUUU | 20 |
| novel_miR_428 | UGAGGUAGUAGGUUGUAUAGUUUA | 24 |
| novel_miR_67 | AAACCCGAAUGAACUUUUUGGA | 22 |
| novel_miR_866 | UAUGGCUUUUCAUUCCUAUGUGA | 23 |
| novel_miR_397 | UGCCCUCCACCUCUGCCCCCAGG | 23 |
| novel_miR_307 | UGGAAGGCCUGGCUUUGCAGCGU | 23 |
| novel_miR_935 | CCAUUUCCUCCGUGCUCGCAGG | 22 |
| novel_miR_725 | AAUCCCAUGGACUGAGGAGCC | 21 |
| novel_miR_939 | UUGGCUCUGCAAGGUCGGCUCAA | 23 |
| novel_miR_327 | UGGAAAAACCCAAACGAACUCU | 22 |
| novel_miR_1087 | UUGGAGGCAUGUCCUUCUCUGCA | 23 |
| novel_miR_220 | AAAAACUGGAAUGAAUUUUUUGG | 23 |
| novel_miR_832 | UACUGUCUGGUGUGGAAGAUGCU | 23 |
| novel_miR_128 | AAAAACUCAAAUGAACUUUUUG | 22 |
| novel_miR_375 | UGGGAACUUGGAGAUCAUCAGA | 22 |
| novel_miR_742 | UCACUCAGUCGUGUCUGACUCC | 22 |
| novel_miR_720 | UACGCCGGGAGCUCGCAGCGC | 21 |
| novel_miR_817 | UCAAAUGCUCAGACUCCUGUGG | 22 |
| novel_miR_9 | CUCACGGCUGCCCCACUCUCCAGG | 24 |
| novel_miR_612 | GUAGUGUUUCCUACUUUAUGGA | 22 |
| novel_miR_703 | UCACUCAGUCGUGUCUGACUCU | 22 |
| novel_miR_673 | UAGGGUGGGCGCUGAGCUCUCC | 22 |
| novel_miR_611 | GACAAGUUCGUUUGGGUUUUU | 21 |
| novel_miR_447 | UGUAACAGCAACUCCAUGUGGACU | 24 |
| oar-miR-26a | UUCAAGUAAUCCAGGAUAGGCU | 22 |
| novel_miR_1 | AAAGCCUAAAUGAACUUUCUGG | 22 |
| novel_miR_47 | UCAGUGCACUACAGAACUUUGU | 22 |
| novel_miR_385 | AAAACCUGAACAAACUUUUUGU | 22 |
| oar-miR-152 | UCAGUGCAUGACAGAACUUGG | 21 |
| novel_miR_917 | UGCAGGAACUUGUGAGUCU | 19 |
| novel_miR_263 | UCCCGUGCUGAUCAGUAGUGGA | 22 |
| novel_miR_267 | AAAAACUCAAAUGAACUUUU | 20 |
| novel_miR_554 | CAAACGAGACUCUCAACCAGGG | 22 |
| novel_miR_403 | GUGGCCUCCAGGGACUUCAGGC | 22 |
| novel_miR_838 | CUGGCCUCUAUUACUGUGACAUC | 23 |
| novel_miR_1009 | CGGAGGCCCGGCCUGAAGGCACC | 23 |
| novel_miR_355 | CCGUGGGAGCAGGCAUCUGCAAC | 23 |
| novel_miR_318 | CAGCCGUCGGGAGGCAGGACGUGU | 24 |
| novel_miR_890 | UUCCAGUUAAUAGCUCUGUUA | 21 |
| novel_miR_583 | UCUGCCCACUGUUUCCUUCCAGC | 23 |
| oar-miR-25 | AUUGCACUUGUCUCGGUCUGA | 21 |
| novel_miR_972 | AGAAAGUUCGUUCAGGUUUUU | 21 |
| novel_miR_132 | AAAACCCAGAUGAACUUUUUGGA | 23 |
| oar-miR-30a-3p | CUUUCAGUCGGAUGUUUGCAG | 21 |
| novel_miR_247 | AACAAUCUGCCGUUUCCCCCACAG | 24 |
| novel_miR_801 | AAAACUAGAAUGAACUUUUU | 20 |
| novel_miR_1061 | AAAACCUGAAUGAACUUUUUGG | 22 |
| novel_miR_332 | CCUGGGGCCAGUGGUGUUUCC | 21 |
| novel_miR_787 | AAAGCCCAAACGAACUUUUUGG | 22 |
| novel_miR_994 | CUGACCUUCUGCUCCCAGGACAGC | 24 |
| oar-miR-16b | UAGCAGCACGUAAAUAUUGG | 20 |
| novel_miR_149 | AAGAACCUGAACGAACCUUUUG | 22 |
| novel_miR_928 | UCCAGACGGUGCUGGCUCUCUGA | 23 |
| novel_miR_541 | UGGCCCUGCUCUGUCCUGUAGC | 22 |
| novel_miR_52 | CCUCUGGGCCCUUCCUCCAGC | 21 |
| novel_miR_746 | AAAACUUGAAUGAACUUUCUGG | 22 |
| novel_miR_87 | UCCUGUGCUCUUUCUCCUGCAGA | 23 |
| novel_miR_694 | UCUGGGCUUUCUGGCAUACAGCU | 23 |
| novel_miR_274 | AAACCCUGAACGAACUUUUUG | 21 |
| novel_miR_738 | CAGUGCAAUGAUAUUGUCAAAGC | 23 |
| novel_miR_862 | AAAAACCUGAAGGAACCUUUU | 21 |
| novel_miR_919 | AAAACCCCAAAUGAACUUUUUGG | 23 |
| novel_miR_183 | AAAAACCCAAAUGAACUUUUUG | 22 |
| novel_miR_320 | UAUGUCGGCUGUUUCUGCAUAC | 22 |
| novel_miR_97 | CGGGGAACUCGUGGGCGCAUC | 21 |
| novel_miR_675 | CCGGCAGCACAGAGCUCAGCUG | 22 |
| novel_miR_828 | AAACCUGGAUAAACUUUUUGGC | 22 |
| oar-miR-323c | CACAAUACACGGUCGGCCUCU | 21 |
| novel_miR_61 | CCUGGAUCCAGAGGGCUCUGGC | 22 |
| novel_miR_614 | UUUUGGGCCUGCAAGUCUUGG | 21 |
| novel_miR_60 | UGAUUGGUACUUCUUAGAGUGA | 22 |
| novel_miR_755 | AAAAGCUGGGUUGAGAGGGCGA | 22 |
| novel_miR_337 | AAGACCCAAACGAACUUUUU | 20 |
| novel_miR_739 | AAAACCCGGAUGAACUUUUUGG | 22 |
| novel_miR_56 | CGCCCUCUGCCCGUGUCGCCAGG | 23 |
| novel_miR_859 | AAACCCGAAUGAACUUCUUUGC | 22 |
| novel_miR_666 | ACUCGGCGUGGCGUCGGUCGUGG | 23 |
| novel_miR_246 | UCUUGUUUCCCCUCCCCGCCAGC | 23 |
| oar-miR-1197-3p | UAGGACACAUGGUCUACUUCU | 21 |
| novel_miR_813 | UGUGCAGAAGCCCUGAGUCCAUG | 23 |
| novel_miR_378 | AACAUUCAUUGUUGUCGGUGGGU | 23 |
| novel_miR_365 | GGCCAGGGGCGUGUCGGGCUCC | 22 |
| novel_miR_834 | AAAAGCCUGAACAAACUUUUUGA | 23 |
| novel_miR_89 | GAGGCCAUGAUCAUCCUUCUGUU | 23 |
| novel_miR_947 | AAAACCUGAAUGAACUUUUUGG | 22 |
| novel_miR_187 | AAACUGGAAUGAACUUUUUGGU | 22 |
| novel_miR_576 | UUGGAAAUGCAGAUUCACGGGCU | 23 |
| novel_miR_1000 | ACCGGUUCUGAUCCCCCUG | 19 |
| novel_miR_718 | CUCGUGGCUCAUGAGCUCUAGA | 22 |
| novel_miR_658 | UACUGUGCCACAGCUGGGUAGA | 22 |
| novel_miR_645 | AAAAACCAGAAGGAACUUUUU | 21 |
| novel_miR_799 | CACUGUAUAGUUACCCUCUUUC | 22 |
| novel_miR_735 | GAAAUGGAGUGCUUGAGAACU | 21 |
| novel_miR_780 | CCUUCCAUCUCGGAUCAGGGC | 21 |
| novel_miR_190 | UCUUUGGUUAUCUAGCUGUAUGA | 23 |
| novel_miR_1045 | GACGCUGGAGUCAGGCUGUGUG | 22 |
| oar-miR-543-3p | AAACAUUCGCGGUGCACUUCUUU | 23 |
| oar-miR-22-3p | AAGCUGCCAGUUGAAGAACUG | 21 |
| novel_miR_858 | AAGACCCAAACGAACUUUUUGG | 22 |
| oar-miR-376e-3p | AACAUAGAGGAAAAUCCACAUU | 22 |
| oar-let-7b | UGAGGUAGUAGGUUGUGUGGU | 21 |
| novel_miR_275 | UCUGAGGCUGCUCAGGCUGUGA | 22 |
| novel_miR_1084 | UCAGCCACUUGUAGGCUCAUCUCC | 24 |
| novel_miR_127 | AAAAAGUUCGUUUGGGUUU | 19 |
| novel_miR_693 | AGCUGGUGUUGUGAAUCAGGCCG | 23 |
| novel_miR_546 | CACAUGGAGUUGCUGUUACAAU | 22 |
| novel_miR_601 | UGCACCAUGGUUGUCUGAGC | 20 |
| oar-miR-103 | AGCAGCAUUGUACAGGGCUAUG | 22 |
| novel_miR_653 | AAAAACUCAAAUGAACUUUUUG | 22 |
| novel_miR_272 | AAAACCUGAAUGAACUUUUU | 20 |
| novel_miR_535 | AUUGGCACGUCUUGGAAUGA | 20 |
| novel_miR_282 | AUGAACUUUUGGGCCAACCCA | 21 |
| novel_miR_1117 | AAAAUCUGAACAAACUUUUU | 20 |
| novel_miR_239 | UUUUUUGCUGGAACAUUUCUGG | 22 |
| novel_miR_951 | AAAGCUGAACGAACUUUUUGGC | 22 |
| oar-miR-30a-5p | UGUAAACAUCCUCGACUGGAAGC | 23 |
| novel_miR_233 | AAAAUCUGAAAUGAACUUUUUGG | 23 |
| novel_miR_178 | UAAGGCACGCGGUGAAUGCC | 20 |
| novel_miR_28 | CCAAACCAGUUGUGCCUGUAGA | 22 |
| novel_miR_623 | UCAAGGUCCGCUGUGAACACGG | 22 |
| novel_miR_1047 | AAAACCCAGAUGAACUUUUUUGG | 23 |
| novel_miR_842 | AAAAACCUGAACAAACUUUUUG | 22 |
| novel_miR_297 | UGUGCAAAUCUAUGCAAAACUGA | 23 |
| novel_miR_68 | CAGUGCCUCGGCAGUGCAGCC | 21 |
| novel_miR_1036 | AGUGGCUGGAAGUUACUGUGC | 21 |
| novel_miR_530 | AUCCGUUGUCUGUAAAGUAGA | 21 |
| novel_miR_380 | AAAAGUUCGUUUGGGUUUUUCC | 22 |
| novel_miR_417 | CUGGGCUGUGUUAGUUCUCUU | 21 |
| novel_miR_393 | AGAAUUGUGGCUGGACAUCUGU | 22 |
| novel_miR_1049 | UGACCUCUGACCUUUGCCUCCCAG | 24 |
| novel_miR_80 | CUGCUCGCCAGGACCCUGACG | 21 |
| novel_miR_708 | UUUUUCAUUAUUGCUCCUGACC | 22 |
| novel_miR_966 | AAAAUCUGAACAAACUUUUGG | 21 |
| novel_miR_1040 | UGGAAAAACCCAAACGAACUCU | 22 |
| novel_miR_671 | AGACUGACUCAGGCUCUGUGUUG | 23 |
| novel_miR_109 | AUCUCGCCCGCAAAGACCCAGC | 22 |
| novel_miR_700 | UCCAGGUCUACGUCUGUCUUCU | 22 |
| novel_miR_2 | AGAAGAUCUGUGGUGGUUCCC | 21 |
| novel_miR_520 | UGCGGGAUCGUUAGUUGUGGCAG | 23 |
| novel_miR_1007 | CAUUAUUACUCACGGUACGAGU | 22 |
| novel_miR_850 | AAAACCUGAAUGAACUUUUUGG | 22 |
| novel_miR_136 | AGGACUGUCCAACCUGAGAAUG | 22 |
| novel_miR_926 | UAGCAGCACAUCAUGGUUUACA | 22 |
| novel_miR_165 | AAAGCUGAACGAACUUUUUGGC | 22 |
| novel_miR_260 | AAAAUCUGAAUGAACUUUUUGG | 22 |
| novel_miR_1109 | CUGACCUAUGAAUUGACAGCC | 21 |
| novel_miR_681 | UCAUUAGGAUGUUGUGAGAGGA | 22 |
| novel_miR_391 | AAACCCGAAUGAACUUUUUG | 20 |
| novel_miR_745 | AAAACCUUGAUGAACUUUUUGA | 22 |
| novel_miR_589 | UUAUAAAGCAAUGAGACUGAUU | 22 |
| oar-miR-495-5p | AGAAGUCGCCCAUGUUCUUUUCG | 23 |
| novel_miR_1016 | AAAAAGUUUGUUCGGGUUUU | 20 |
| novel_miR_933 | AGAAGUUCGUUUGGAUUUUCCC | 22 |
| novel_miR_227 | AUCAUGUAUGAUACUGCAAACA | 22 |
| novel_miR_156 | GAAAGUGUAAGGCAAGGUCUGGU | 23 |
| novel_miR_931 | UAAUGGAGGACUUCCCUGGUGG | 22 |
| novel_miR_8 | ACCCUGCAGCCAAAGAAGCUAC | 22 |
| novel_miR_333 | AAAAUCUGAACAAACUUUUU | 20 |
| novel_miR_138 | ACUGGACUUGGAGUCAGAAGGC | 22 |
| novel_miR_514 | UGGGACUGUGAUCUGGGAGAGU | 22 |
| oar-miR-376a-5p | UAGAUUCUCCUUCUAUGAGUAC | 22 |
| novel_miR_306 | AAAAACCCAAGUGAACUUUUUG | 22 |
| novel_miR_120 | UGACCGAUUUCUCCUGGUGUU | 21 |
| novel_miR_358 | UCUCGACCGUCAGAGCACCCUUC | 23 |
| novel_miR_1116 | AAAAUCUGAACAAACUUUUU | 20 |
| novel_miR_912 | AAAAACCUGAACGAACUUUUU | 21 |
| novel_miR_654 | UGGAAUGUAAAGAAGUAUGUAU | 22 |
| novel_miR_956 | UGACAUCUUGUUCUCUUCAGG | 21 |
| novel_miR_880 | UAGAGAAGCGCUGGGGGAAAGC | 22 |
| novel_miR_883 | UGAGCAAGACUGUUUUUCUGUGA | 23 |
| novel_miR_313 | AAAAAGUUCGUUUGGGUUUUUC | 22 |
| novel_miR_1029 | UGGCCUGGUGGUCUGCCCCUCA | 22 |
| novel_miR_1101 | CACCCGUCCCGUGCGUCCCCGGA | 23 |
| novel_miR_107 | CUCGAGUGCUGAAGUGACCUUCU | 23 |
| novel_miR_123 | CUAUACAAUCUAUUGCCUUCCC | 22 |
| oar-miR-376b-3p | AUCAUAGAGGAAAAUCCAUGU | 21 |
| novel_miR_521 | UUUCUUUGAGCUUCUUUCUUUC | 22 |
| novel_miR_560 | UAUAUGUGUGUAUGUGUAUAUA | 22 |
| novel_miR_442 | CAGCAGCAAUUCAUGUUUUGA | 21 |
| novel_miR_485 | UACUAGGUACUGAGGAUACAGCA | 23 |
| novel_miR_1035 | CAUAGCCCCGGUGCGGAGCCUGGC | 24 |
| novel_miR_1017 | CACAGCAAGUGUAGACAGGCA | 21 |
| novel_miR_580 | UUCAAGGCUGAAAUGUACAAC | 21 |
| novel_miR_1023 | GUGGACUUCCUUGGUAGCUC | 20 |
| novel_miR_230 | UUGGGGAAAACAGGCUCAGG | 20 |
| novel_miR_441 | AAAACCUGAAAGAACUUUCUGG | 22 |
| novel_miR_545 | UCUGAGACAGGAGGACAGGUGA | 22 |
| novel_miR_511 | GAGGGGUUUGGGCUGUGCUUCU | 22 |
| oar-miR-410-3p | AAUAUAACACAGAUGGCCUGU | 21 |
| novel_miR_507 | AUUGUCCUUGCUGUUUGGAGAU | 22 |
| novel_miR_1022 | UAGCGGAGAUUGCCAAAGUAGA | 22 |
| novel_miR_77 | AAAAGUUCGUUUGGGUUUUUCC | 22 |
| novel_miR_494 | AAAAUCUGAACGAACUUUUUGGU | 23 |
| novel_miR_280 | UGAGUGUGUGUGUGUGAGA | 19 |
| novel_miR_435 | UUCACAGUGGCUUUCUGC | 18 |
| oar-let-7c | UGAGGUAGUAGGUUGUAUGGUU | 22 |
| novel_miR_500 | AGAAAAACCUGAAUGAACUUU | 21 |
| novel_miR_557 | UGAUUGGCAUUUCUUAGAGUGA | 22 |
| novel_miR_627 | UAACACAUGUCAUAACGCGGGGC | 23 |
| novel_miR_373 | UGGAGGCCUGCAAACUGUAGGG | 22 |
| novel_miR_26 | AAACUUGAGCAAACUUUUUGGC | 22 |
| novel_miR_414 | CUGACCCCCGCCCUCCCUGCAGC | 23 |
| novel_miR_988 | UCUCAUCACGUUCCUUUCUGCCAGC | 25 |
| novel_miR_844 | AAAUCUGAACAAACUUUUUGGC | 22 |
| novel_miR_946 | AAAUCUGAACAAACUUUUUGGC | 22 |
| novel_miR_5 | AAGGCCAUGCUCCACAGUGAGC | 22 |
| novel_miR_498 | CUAACCUGGAUGAACUUUUUGG | 22 |
| novel_miR_126 | AAAAGUUCAUUAGGGUUUUUC | 21 |
| oar-miR-379-5p | UGGUAGACUAUGGAACGUAGGC | 22 |
| novel_miR_480 | UACUGUGCCACGGAUGGGUAGC | 22 |
| novel_miR_114 | AAAACCAGAACGAACUUUUUGU | 22 |
| novel_miR_578 | CUAGACUGAAGCUCCUUGAGG | 21 |
| novel_miR_1060 | UGCCUCUCCGCCACCUCCACCUC | 23 |
| novel_miR_302 | AAUGAACCUUGGAAAACUUUGGC | 23 |
| novel_miR_96 | CAGCAGCACACUGUGGUUUGUA | 22 |
| novel_miR_405 | AAAGUUCGUUCAGGCUUUUCUG | 22 |
| novel_miR_139 | UCUGCAUCCGAGGACUCAACCC | 22 |
| novel_miR_27 | AAAAUCUGAGCAAACUUUUUGG | 22 |
| novel_miR_721 | UCUCUGUUUUCUGACCCACAGA | 22 |
| novel_miR_103 | UUGCUAUAGCCUCCUUGCCUCU | 22 |
| novel_miR_1003 | UUUCAUGUUGAGCCUCAGAUCGC | 23 |
| novel_miR_1097 | UGGAGCCGGAGCUGGUUAGC | 20 |
| novel_miR_641 | UCACUCAGUCGUGUCUGACUCU | 22 |
| novel_miR_440 | AAAAACUCAAAUGAACUUUUUG | 22 |
| novel_miR_105 | AAAGGCCCAAAUGAACUUUUUG | 22 |
| novel_miR_785 | UUAAGACUUGCAGUGAUGUUU | 21 |
| novel_miR_1080 | AAUAUCUGAAUGAACUUUUUGGC | 23 |
| novel_miR_496 | AAAAUCUGAGUGAACUUUUUGG | 22 |
| novel_miR_453 | AUGUCCGCGGGUUCCCUAUCC | 21 |
| novel_miR_269 | CCAGGAGUGUCUUUGGAUUGUC | 22 |
| novel_miR_610 | ACCUUGGCUCUAGACUGCUUACU | 23 |
| oar-miR-199a-3p | ACAGUAGUCUGCACAUUGGUU | 21 |
| novel_miR_102 | UCACUUCAGUCUUUUCUUUCUAGC | 24 |
| novel_miR_119 | UCACUUCAGUCGUGUCCGACUCU | 23 |
| novel_miR_439 | CUCCGUUUGCCUGUUUUGCUGA | 22 |
| oar-miR-487b-5p | AGUGGUUAUCCCUGUCCUGUUC | 22 |
| novel_miR_479 | AAAACCUGAAUGAACUUUUUGG | 22 |
| oar-miR-411a-5p | AUAGUAGACCGUAUAGCGUACG | 22 |
| novel_miR_278 | GUGCUCGUCCCGUCCUCCAGG | 21 |
| novel_miR_792 | UCAGCCCUUAUUGCCUGAGC | 20 |
| novel_miR_36 | UCCCCCAUGUGUCUCCUCGCCCAGG | 25 |
| novel_miR_305 | UUGGAGGUUGGGAGCGGGGAGAGA | 24 |
| novel_miR_1082 | UAUGCCGGCUAACCAUAGCUU | 21 |
| novel_miR_463 | UAAUCUCAGCUGGCAACUGUG | 21 |
| novel_miR_366 | AAAAACCCAAAUGAACUUUUUG | 22 |
| novel_miR_443 | UUAAUUUUUGCAAGGCUUUUCC | 22 |
| novel_miR_244 | UGUAAGGCCAGGCUUCAUGAGC | 22 |
| novel_miR_294 | UGGUCUGCUCAGCUGGAAUGGG | 22 |
| novel_miR_369 | AAAACCUGAAUGAACUUUUUGG | 22 |
| oar-miR-411a-3p | UAUGUAACACGGUCCACUAAC | 21 |
| novel_miR_1128 | CACAUGGAGUUGCUGUUACA | 20 |
| oar-miR-329b-5p | GAGGUUUUCUGGGUUUCUGUUUC | 23 |
| novel_miR_908 | AAAAACUCAGAUGAACUUUUGG | 22 |
| novel_miR_1127 | AAAAGCUGAGUGAACUUUUUGGU | 23 |
| novel_miR_741 | ACUGGACUUGGAGUCAGA | 18 |
| novel_miR_525 | AAACCCUGAAUGAACUUUUUGG | 22 |
| novel_miR_568 | UUAUUUAUUUUGGGGCUUCUCU | 22 |
| novel_miR_932 | UCCGUCCCUUCUUGCCCACCAG | 22 |
| novel_miR_585 | AAAAAACUGAAUGAACUUUUUGU | 23 |
| novel_miR_682 | AAAAGAGCUGUUGAUGAACUAGU | 23 |
| novel_miR_203 | AAAACCUGAAUGAACUUUUU | 20 |
| novel_miR_235 | AGAGAGCGACUGCGCUUAGAAGA | 23 |
| novel_miR_1032 | AUGCAAACGAACUUUUUGGCCA | 22 |
| novel_miR_106 | UCAGUAAAUGUUUGUUGGAUA | 21 |
| novel_miR_891 | CCCGAGGAGUCUGGGCCGGUUUC | 23 |
| oar-miR-3956-3p | UCUGACCUUCCUGCAUCCCGUUC | 23 |
| novel_miR_159 | UGGUGGAACCGGGUCCUGGAU | 21 |
| novel_miR_622 | UUAAUGCUAAUCGUGAUAGGGGU | 23 |
| oar-miR-323a-3p | CACAUUACACGGUCGACCUCU | 21 |
| novel_miR_231 | UGAGGUAGUAAGUUGUAUUGUU | 22 |
| novel_miR_237 | AAAAACCUGAACAAACUUUUUG | 22 |
| novel_miR_292 | UUCUCCUAUAGAUGCCGUCACAG | 23 |
| novel_miR_621 | UUUCCUCAUAUUCAUUCAGGAGU | 23 |
| novel_miR_669 | AAAAACCCAAAUGAACUUUUUG | 22 |
| novel_miR_21 | UGCGCGCACGCUCACUCUGGCG | 22 |
| novel_miR_236 | AAAAUCCGAAUGAACUUUUUGGU | 23 |
| novel_miR_815 | GAGGGCUGCCGCCUCUUCGGC | 21 |
