# Supplementary material for: Maternal undernutrition inhibits fetal rumen development: novel miRNA-736-mediated dual targeting of E2F2 and MYBL2 in sheep
Source: J Anim Sci Biotechnol. 2026 Jan 6;17:2. doi: 10.1186/s40104-025-01321-7 (PMC12771908; doi:10.1186/s40104-025-01321-7)
Supplement: Supplementary file 2 — Additional file 2: Fig. S1. The volcano plots and correlation analysis in undernourished and nutrition-recovery models. Fig. S2. Functional analysis of miRNA and its target genes in fetal rumen. Fig. S3. Construction of 3' UTR vectors for E2F2 and MYBL2 [file 40104_2025_1321_MOESM2_ESM.docx]

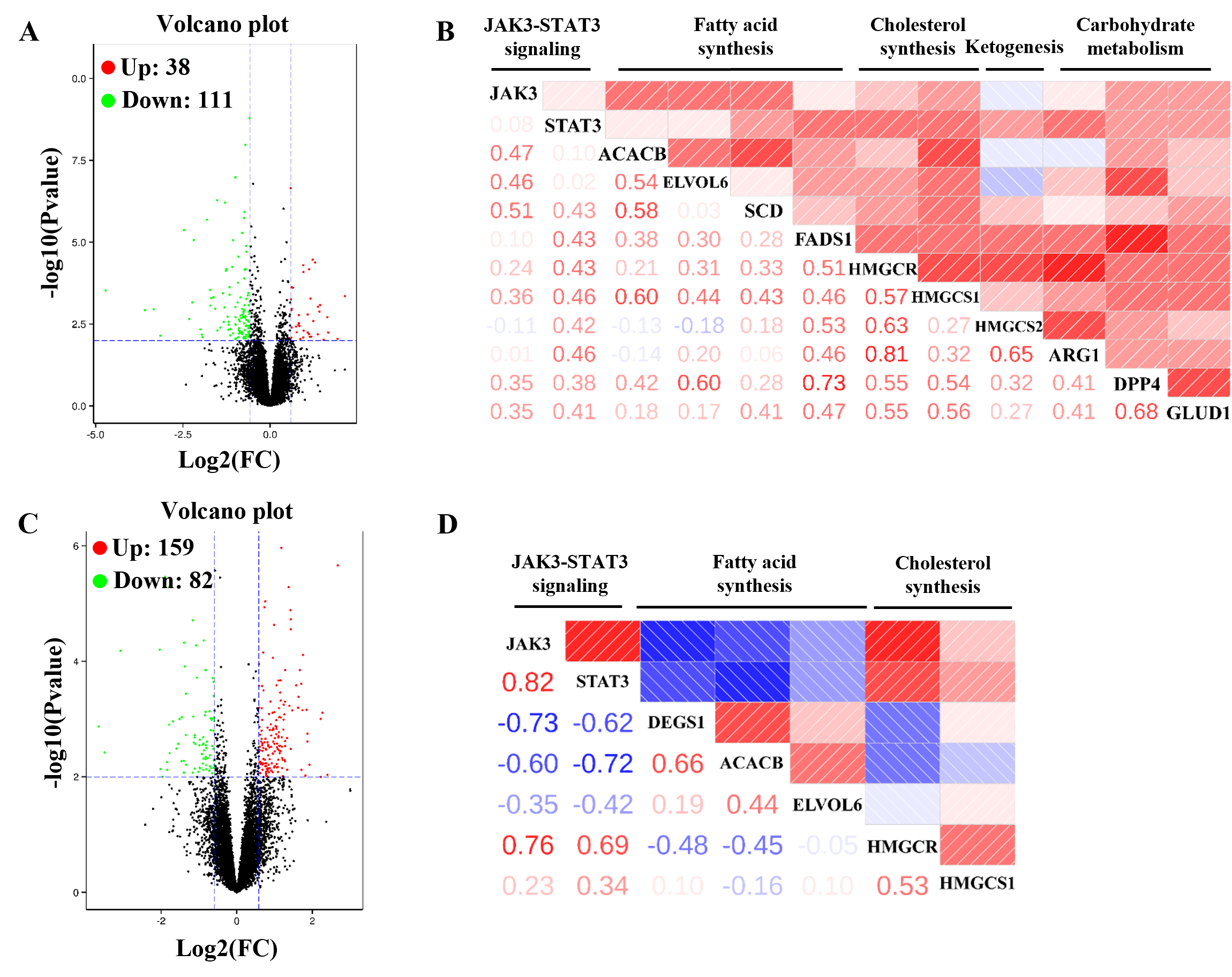


**Fig. S1.** The volcano plots and correlation analysis in undernourished and nutrition-recovery models. **A** The volcano plots of total genes in fetal rumen of undernourished ewe models. **B** Correlation analysis of gene expressional levels between *JAK3* and *STAT3* and nutrient metabolism in fetal rumen of undernourished ewe models. **C** The volcano plots of total genes in fetal rumen of nutrition-recovery ewe models. **D** Correlation analysis of gene expressional levels between *JAK3* and *STAT3* and nutrient metabolism in fetal rumen of nutrition-recovery ewe models.


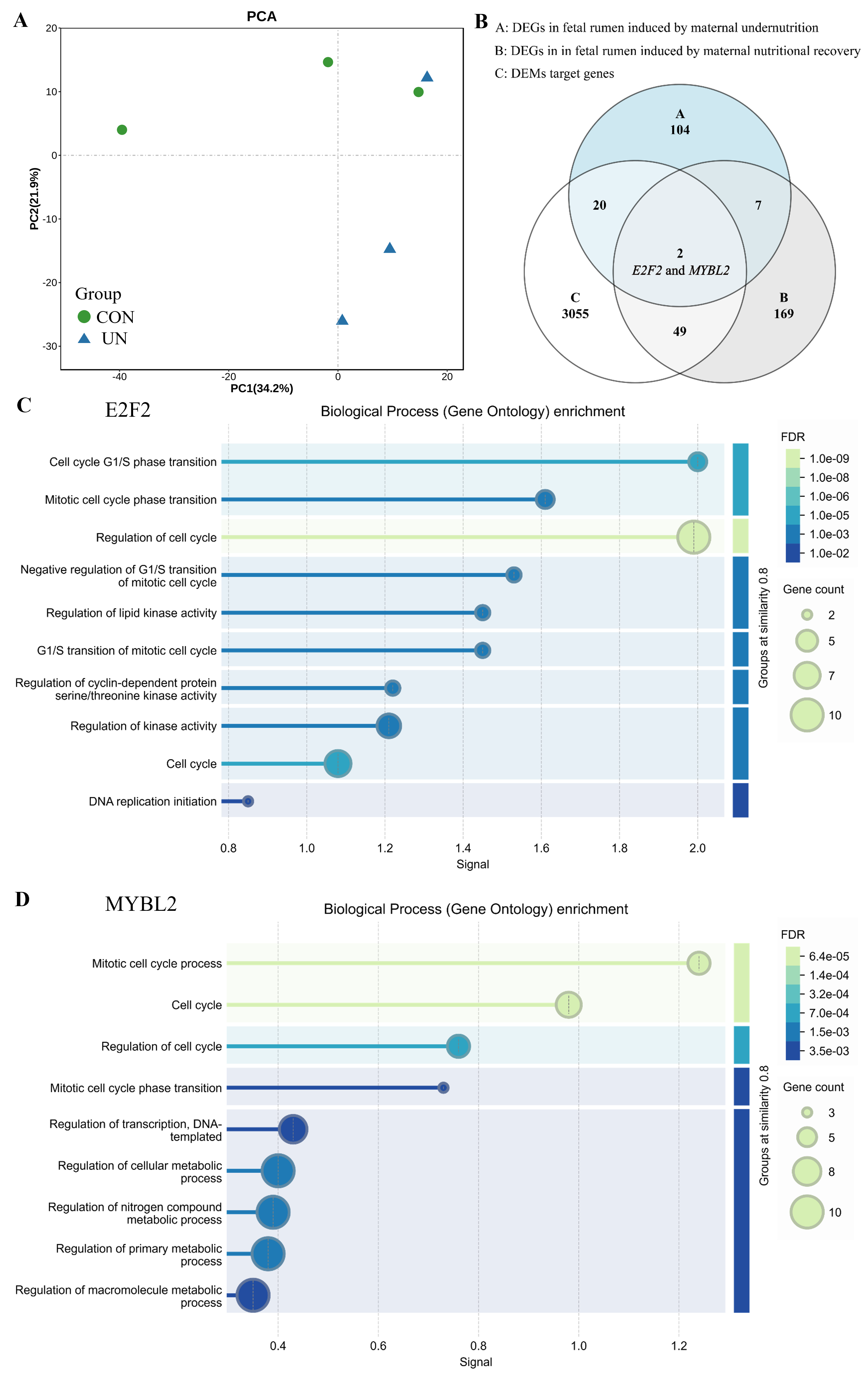


**Fig. S2.** Functional analysis of miRNA and its target genes in fetal rumen. **A** PCA of total miRNA expression in rumen of the CON and UN groups. **B** Both intersection analysis of DEGs in fetal rumen of undernourished ewes and nutritional recovery and miRNA target gene in fetal rumen of undernourished ewes. **C-D** Analysis of biological processes in protein-interacting genes with *E2F2* and *MYBL2*.


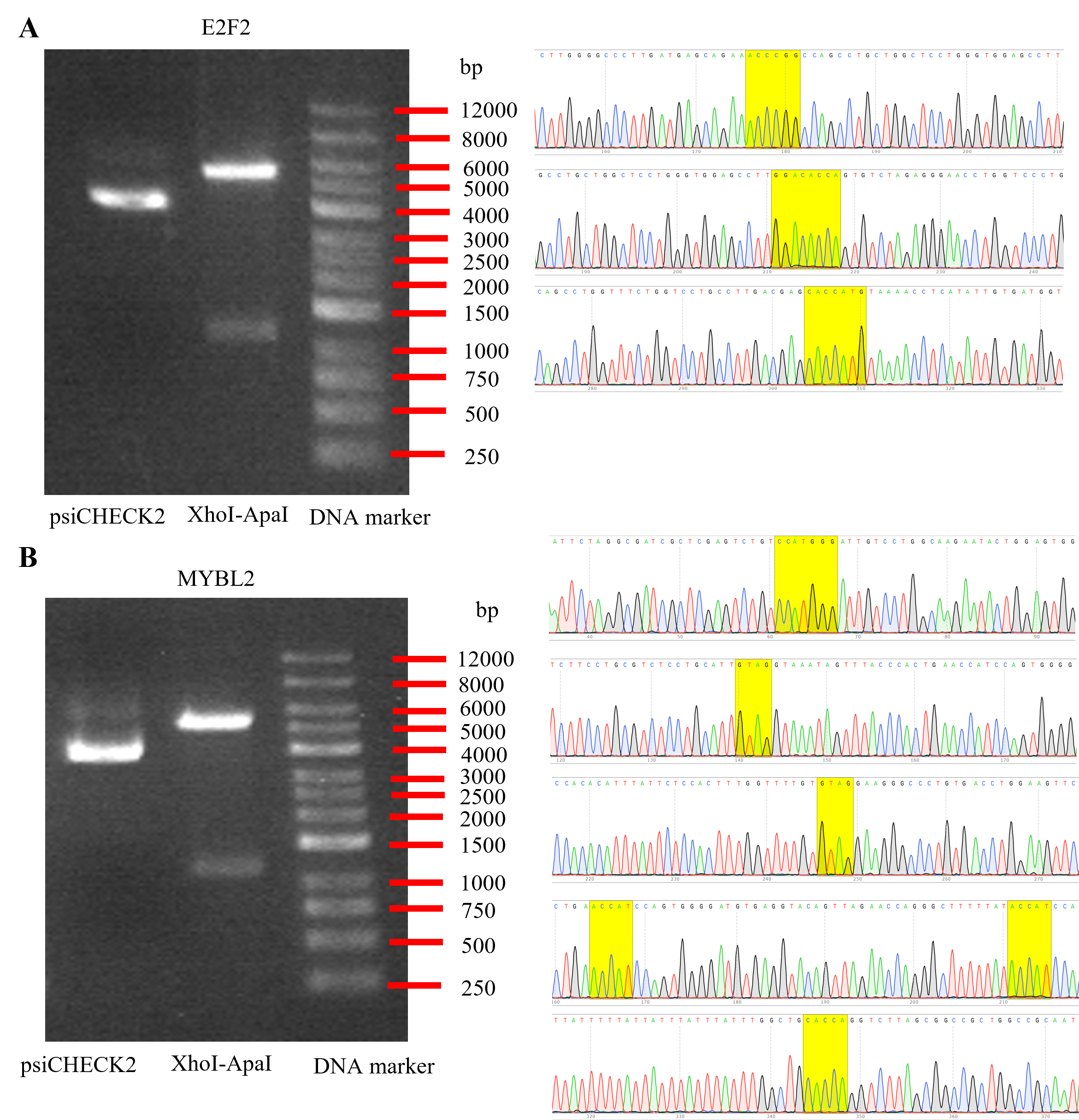


**Fig. S3.** Construction of 3 'UTR vectors for *E2F2* and *MYBL2*. **A** *E2F2* vector double enzyme digestion and target sequencing results. **B** *MYBL2* vector double enzyme digestion and target sequencing results.
